# Phylogenomic Identification of a Highly Conserved Copper-Binding RiPP Biosynthetic Gene Cluster in Marine *Microbulbifer* Bacteria

**DOI:** 10.1021/acschembio.5c00507

**Published:** 2025-09-19

**Authors:** Yifan Tang, Weimao Zhong, Longping Fu, Emmanuel Asante, Anastasiia Kostenko, F. N. U. Vidya, Paige Mandelare-Ruiz, Tamilore T. Adeogun, Gabriel P. Anderson, Benjamin E. Edmonds, Oscar Fang, Michelle Han, Alia S. Hollingsworth, Amna R. Ingham, Carlyn R. Kirby, Alice Landrum, Connor R. Mack, Nikki S. Nobari, Emma J. Oswald, Cecilia L. Polevoy, Yasmin Sharifian, Timothy J. So, Joelee R. Stokes, Reniya S. Thompson, Rishabh Vuthamaraju, Elaine C. Wang, William H. Yang, Alison E. Onstine, Valerie J. Paul, Ronghu Wu, Allegra T. Aron, Vinayak Agarwal

**Affiliations:** 1 School of Chemistry and Biochemistry, 1372 Georgia Institute of Technology, Atlanta, Georgia 30332, United States; 2 Department of Chemistry and Biochemistry, 2927 University of Denver, Denver, Colorado 80210, United States; 3 Smithsonian Marine Station, Ft Pierce, Florida 34949, United States; 4 School of Biological Sciences, Georgia Institute of Technology, Atlanta, Georgia 30332, United States

## Abstract

Conserved biosynthetic gene clusters (BGCs) are often tied to the production of natural products that perform critical functions in an organism’s physiology and ecological interactions. Here, by phylogenetic analysis across the bacterial genus, we report the obligate conservation of a BGC in genomes of cosmopolitan marine *Microbulbifer* bacteria. This genus is a common member of marine microbiomes, and this BGC was conserved in *Microbulbifer* genomes regardless of phylogenetic or geographical dispersal. The post-translationally modified peptidic product encoded by this BGC—which was accessed via heterologous production and its structure elucidated using a combination of mass spectrometry and NMR spectroscopy—was found to be a copper chelator. Similar BGCs were then found in genomes of other marine bacterial genera coinhabiting the microbiomes of sponges and corals. The phylogenomic workflows described herein were implemented in a pedagogic setting at the Georgia Institute of Technology to provide hands-on instruction to undergraduate students in bacterial phylogeny, genome mining, and natural product chemistry.

## Graphical abstract

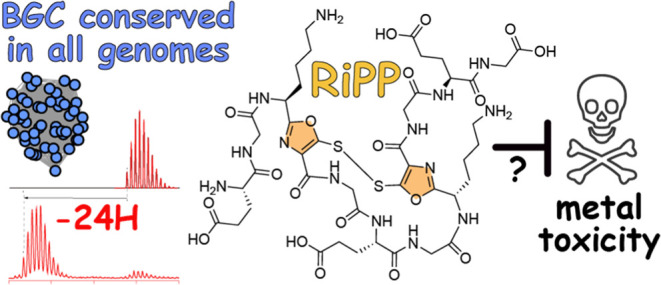

## Introduction

Marine invertebrates such as sponges and corals are ecosystem engineers; both create habitat and enrich biodiversity, and the water filtering activity of sponges is critical for nutrient circulation in the benthic environment.^1,2^ These marine invertebrates are holobionts, in that the eukaryotic host is associated with an obligatory symbiotic microbiome and a commensal microbiome. Among these, the obligatory symbiotic microbiome is well validated to be a prolific producer of bioactive small molecules that bear the moniker natural products.^3–5^ Increasing sophistication of genomic tools and bioinformatic workflows has established that the eukaryotic hosts are likewise natural product producers.^6–9^ Natural products produced by the obligatory symbiotic microbiome and by the eukaryotic host are postulated to serve ecological roles of defense and chemical communication in the benthic marine environment.^10,11^

Unlike members of the obligatory symbiotic microbiome that often resist cultivation in the laboratory, members of the loosely associated commensal microbiome can be cultivated in the laboratory. Gram-negative Pseudomonadota dominate the marine commensal microbiomes, though that could be reflective of experimental bias introduced by bacterial culturing methodologies.^12^ Natural product isolation studies on individual strains of sponge- and coral-derived commensal bacteria have established that they hold underutilized promise for bioactive natural product discovery.^13–17^ Underlying these missed opportunities for chemical discovery is the lack of robust phylogenetic inventory among several well represented marine commensal bacterial genera that could guide strain prioritization for natural product discovery.

Members of the Gram-negative *Gammaproteobacteria* genus *Microbulbifer* are commonly associated with corals and sponges, other marine invertebrates, and marine sediments.^18^ Typified by their proclivity to degrade polymeric matrices, *Microbulbifer* strains are now established to be sources of natural products and enzymatic novelty.^15–17,19,20^ Isolation of over a hundred *Microbulbifer* strains has been reported, including our large-scale culturing effort from marine sponges and corals collected in the Florida Keys.^18,21^ However, as mentioned before, a global phylogenetic inventory of the genus is missing, as is the phylogenomic collation of the natural product biosynthetic gene clusters (BGCs) encoded within *Microbulbifer* genomes. The widespread conservation of BGCs often implies important ecological and organismal roles for the encoded natural products.^22–24^ In themselves, functional relevance can bias natural products to possess pharmaceutically desirable bioactivities.^25,26^ Whether *Microbulbifer* bacteria possess conserved BGCs or not in the context of BGC diversity and natural product biosynthetic potential is presently not known.

In this study, using a library of 36 sponge-derived and 6 coral-derived strains that we have recently cultured and mining all available genomic information in public databases, we develop the first of its kind robust phylogenetic inventory and species distribution for the *Microbulbifer* genus. We demonstrate that while natural product BGC distribution is species specific and is highly directed toward producing small molecule metallophores, a BGC encoding a ribosomally synthesized and post-translationally modified peptide (RiPP) is conserved across all *Microbulbifer* strains regardless of geographical and phylogenetic distribution.^27^ The encoded RiPP is accessed using synthetic biology principles and is structurally characterized. While it is tantalizing to posit that the conservation of this BGC in *Microbulbifer* genomes is tied to an ecological and/or organismal role of the RiPP, the BGC is found to be constitutively silent under permissive and stressed growth conditions. The findings reported herein now provide the foundational genomic basis for interrogating chemical interactions among the *Microbulbifer* genus and for targeted access to novel natural products by using cultivation-based or synthetic biological workflows.

## Results and Discussion

### Phylogeny of *Microbulbifer* Strains

We have previously reported the isolation of *Microbulbifer* strains from high microbial abundance marine sponges *Smenospongia aurea*, *Aplysina fulva*, and *Aiolochroia crassa* that were collected in the Florida Keys.^21,28^ Similar well-established workflows were used for the isolation of *Microbulbifer* strains from the corals *Colpophyllia natans*, *Diploria labyrinthiformis*, and *Pseudodiploria strigosa* as well. A subset of 36 sponge-derived and 6 coral-derived strains could be reproducibly cultivated in the laboratory without loss of viability and batch-to-batch variability. Genomic DNA isolated from these 42 strains was sequenced, and draft genomes were assembled. These draft genomes were queried together with 25 genome assemblies of *Microbulbifer* strains available in GenBank, and 7 additional strains for which only the 16S rRNA gene sequences were available (Table S1). From the assembled genomes, the 16S rRNA gene sequences were retrieved using Barrnap, aligned using MAFFT, and a maximum-likelihood phylogenetic tree was constructed using IQ-TREE.^29,30^ The tree was rooted using three Proteobacterial 16S rRNA gene sequences. This workflow is illustrated in Figure S1. Next, 15 housekeeping gene sequences were retrieved from 70 draft genomes (42 *Microbulbifer* genomes sequenced in this study, 25 *Microbulbifer* genome assemblies, and 3 root genomes obtained from GenBank) (Table S2). The housekeeping gene sequences were concatenated to develop a multilocus sequence analysis (MLSA) scheme, which was used to construct another maximum-likelihood phylogenetic tree (Figure S1). Phylogenetic assignment using MLSA accounted for single gene sequence bias by evaluating multiple housekeeping genes that could have evolved differently from the 16S rRNA gene.^31^ The improved resolution of the MLSA-based maximum-likelihood tree was supported by the Shimodaira–Hasegawa approximate likelihood ratio test and ultrafast bootstrap values calculated by IQ-TREE (Figures S2 and S3).^29,32–34^ The two phylogenetic trees were highly congruent, revealing a species-level distribution within the *Microbulbifer* genus (Figure 1).

**Figure 1:**
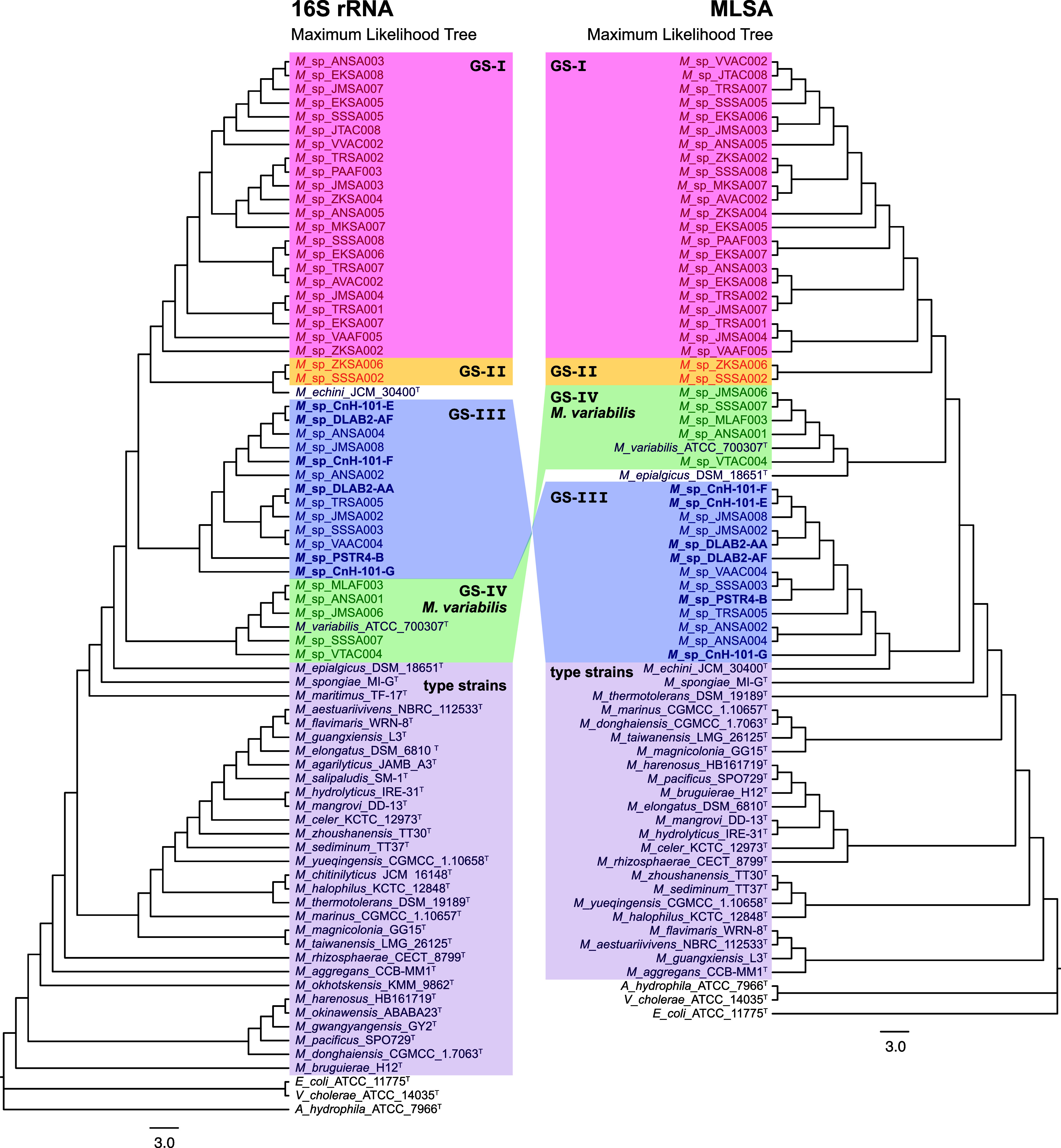
Maximum-likelihood phylogenetic trees generated using 16S rRNA gene sequences (left) and MLSA (right). Three strains—*Escherichia coli* ATCC 11775, *Vibrio cholerae* ATCC 14035, and *Aeromonas hydrophila* ATCC 7966—are selected as outgroups to root the tree. Note that strains for which 16S rRNA gene sequences are available outnumber the strains for which draft genomes are available that are used to construct the MLSA tree. The genomic species (GS) identified in this study (GS-I–IV) are denoted in different colored boxes as compared to strains described in the literature. The coral-derived strains sequenced as part of this study are shown in boldface; they all localize in the GS-III group. The GS-IV group includes the type strain *M. variabilis* ATCC 700307, while GS-I–III do not contain any known type strains. The bootstrap values are illustrated in Figures S2 and S3.

The *Microbulbifer* strains sequenced as a part of this study fell within four species that we designated as genomic species 1–4 (GS-I–IV). GS were delineated through strains sharing average nucleotide identity (ANI) scores above 95% (vide infra) and supported by the sH-ALRT and ultrafast bootstrap values of the 16S and MLSA trees (Figures S2 and S3). In the absence of species-defining phenotypic or metabolic examination of all new strains described herein, we are conservatively restricting their annotation as GS.^35^ Of these, only the GS-IV group is associated with a type strain—*Microbulbifer variabilis* ATCC 700307, which was isolated from marine algae.^36^ The other three groups—GS I–III—likely represent new *Microbulbifer* species. Interestingly, all 6 coral-derived *Microbulbifer* strains (CnH-101-E, CnH-101F, CnH-101-G, DLAB2-AA, DLAB2-AF, and PSTR4-B) were placed in the GS-III clade (Figure 1), which also contained sponge-derived strains.

The genomic loci-based phylogenetic distributions were supplemented by a whole genome ANI-based grouping of *Microbulbifer* strains to provide greater resolution and support for the species distribution (Figures 2, S4–S6, Supporting Information, Data set D1).^37–39^ For the 22 strains grouped as GS-I, two strains grouped as GS-II, and 13 strains grouped as GS-III, their closest ANI scores are to a known type strain *M. variabilis* ATCC 700307 were 86.8%, 84.3%, and 88.4%, respectively; ANI scores of less than 95% were supportive of their annotations as new *Microbulbifer* species.^40^ The three GS in themselves are conspicuous in their separation within the ANI similarity matrix (Figure 2). In contrast, the five strains grouped as GS-IV demonstrated the highest ANI score of 99% to *M. variabilis* ATCC 700307, classifying them as *M. variabilis* strains. Taken together, this is the first of its kind phylogenetic interrogation of the *Microbulbifer* genus and provides the foundation for intra- and interspecies comparisons.

**Figure 2:**
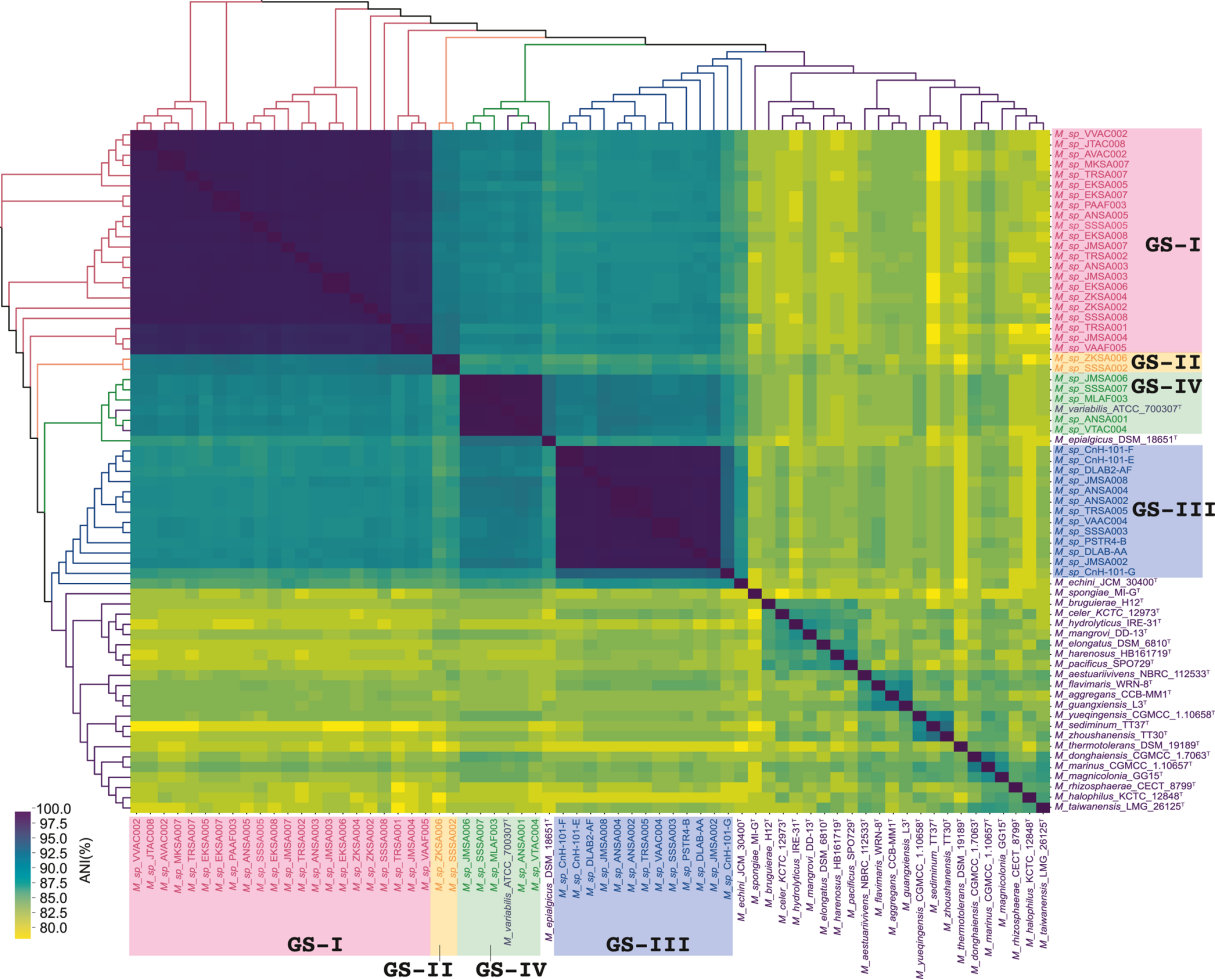
A similarity matrix illustrated as a heat map colored according to average nucleotide identity (ANI) expressed as a percentage. The phylogenetic tree on both axes were reconstructed with the neighborhood-joining method using the pairwise distances calculated from ANI similarity scores. The four GS identified in this study are indicated.

### Natural Product BGC Diversity in *Microbulbifer* Genomes

With the phylogenetic assignments of *Microbulbifer* strains in hand, we next explored their genetic potential for natural product biosynthesis. For strains sequenced in this study and the strains for which draft genomes were available, the natural product BGCs were mined using antiSMASH.^41^ Most *Microbulbifer* strains possess a modest number—less than ten—BGCs, which is comparable to the number of BGCs reported to be present in other marine Proteobacteria.^42–45^ The *Microbulbifer* BGCs were then organized into gene cluster families (GCFs) using BiG-SCAPE.^46^ These GCFs are illustrated as a binary presence/absence matrix against the *Microbulbifer* MLSA-based phylogenetic tree in Figure 3. Visualizing the correlation between the GCF distribution and strain phylogeny allowed for some key inferences to be made.

**Figure 3:**
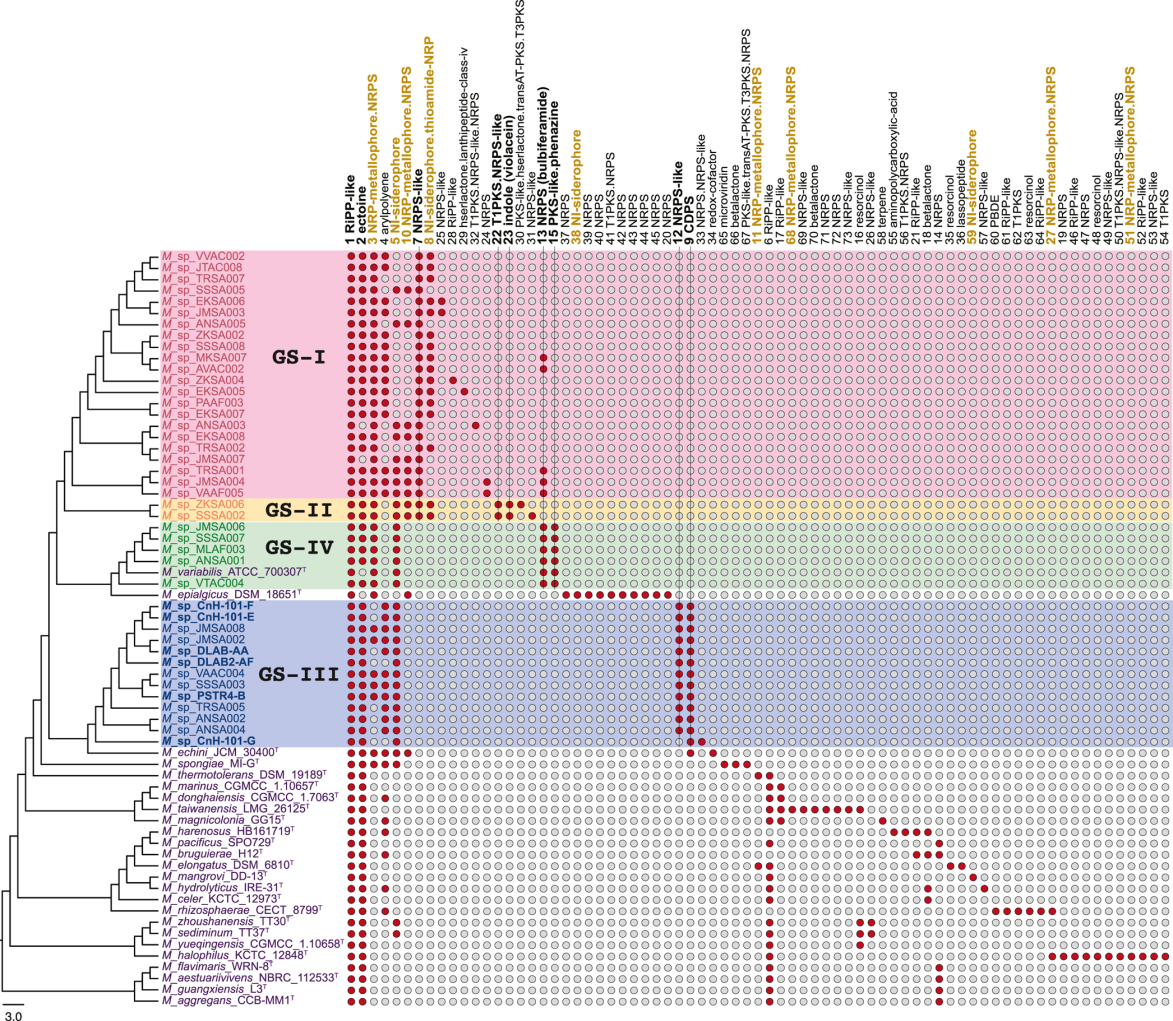
A gene cluster family (GCF) presence/absence matrix for the *Microbulbifer* genus. The phylogenetic tree and the GS grouping are reproductions of the MLSA tree illustrated in Figure 1. Some of the GCFs discussed in text are identified by vertical lines. The metallophore GCFs are highlighted in brown text.

Species-specific differences in the GCF distribution were immediately apparent. The coral and sponge-derived GS-III strains were typified by the presence of two GCFs—GCF 12 and GCF 9—that comprise BGCs encoding a single module nonribosomal peptide synthetase (NRPS) and a cyclodipeptide synthetase (CDPS), respectively. The products of both BGCs are as yet cryptic. The GS-IV strains were conspicuous in the conservation of GCF 13 that was comprised of the *bulb* BGCs encoding production of bulbiferamides.^17^ The genetic potential for bulbiferamide production was not limited to GS-IV strains and was present in some GS-I strains as well, though not all GS-I strains harbored the *bulb* BGC. Although no phenazines have been described from *Microbulbifer*, the presence of GCF 15 in GS-IV strains pointed to the possibility of their production. Though phenazine biosynthesis is broadly distributed in bacteria, in *Microbulbifer*, it is restricted to the GS-IV strains only.^47^ The GCF 23 uniquely present in GS-II strains likely encodes the production of violacein, which was supported by the purple coloration of GS-II strains *Microbulbifer* sp. ZKSA006 and *Microbulbifer* sp. SSSA002 (Figure S7). It is noteworthy that violacein has been isolated from *Microbulbifer* bacteria, and its production is widespread in marine Proteobacteria.^48,49^ The two GS-II strains also possess GCF 22 for which the products are cryptic. At present, the organismal and/or ecological implications for the presence of species-specific GCFs in *Microbulbifer* bacteria is not immediately apparent but serve to provide a genomic foundation on which targeted metabolome mining and synthetic biology efforts can be built for natural product discovery. Another observation from the GCF presence/absence matrix was the proclivity of *Microbulbifer* bacteria to synthesize siderophores.^50^ These GCFs include NRPS-dependent siderophores (such as GCFs 3 and 10), and NRPS-independent siderophores (GCFs 5 and 8). No siderophores have been isolated from *Microbulbifer* bacteria to date. The GCF 2 that encoded production of ectoine was broadly conserved in *Microbulbifer* strains; the production of ectoine in marine bacteria is well precedented as it acts as an osmoprotectant in a saline environment (Figure 3).^51^

Interestingly, independent of strain phylogeny, the holobiont source (coral or sponge), or geographical distribution, we observed the obligate conservation of GCF 1 in all strains described in this study, including the *Microbulbifer* type strains. The BGCs in GCF 1 were annotated to furnish a RiPP.^27^ The GCF 1 BGCs were different from the RiPP-encoding genomic loci in that they have been reported to be widely conserved in symbiotic microbiomes of marine sponges as well as in the sponge eukaryotic hosts.^7,52,53^ While peptidic natural products have been described from *Microbulbifer* strains, no RiPPs are yet reported.^15–17^ Thus, deciphering the chemical product encoded by GCF 1 and its possible roles in *Microbulbifer* organismal physiology or ecological interactions was of primary interest. A de novo biosynthetic construction of the natural product from the above-mentioned RiPP GCF was undertaken.

### Identifying the RiPP Product from the Conserved BGCs

The BGCs that constitute GCF 1 encoded a multinuclear nonheme iron-dependent oxidase (MNIO) together with a protein annotated as a domain of unknown function 2063 (DUF2063).^54^ The presence of the MNIO together with a Cys-rich precursor peptide (vide infra) was immediately reminiscent of methanobactin biosynthesis, leading us to annotate the GCF 1 BGCs as Microbulbifer-derived methanobactin-like RiPP BGCs (*mmr* BGCs) (Figure 4A).^55^ Pursuant to this logic, the *mmrA* open reading frame encoded the putative RiPP precursor peptide, which would be post-translationally modified by the MNIO encoded by the *mmrB* gene with the likely contribution of the DUF2063-encoding *mmrC* gene. The RiPP precursor peptides are divided into an N-terminal leader region that binds to the peptide-modifying enzymes, but it is itself not modified, and a C-terminal core that is post-translationally modified.^27^ The MmrC polypeptide possessed a RiPP recognition element domain, which was modeled to engage the MmrA leader peptide (Figure S8).^56,57^ The MmrA precursor peptides demonstrated a high degree of conservation of the N-terminal leader region, while variability was observed in the C-terminal core region, with the number of Cys residues embedded in the repeating “EGKCG” units varying from five to eight (Figure S9). Repetitive units have been observed in other RiPP precursor peptides.^58–60^

**Figure 4:**
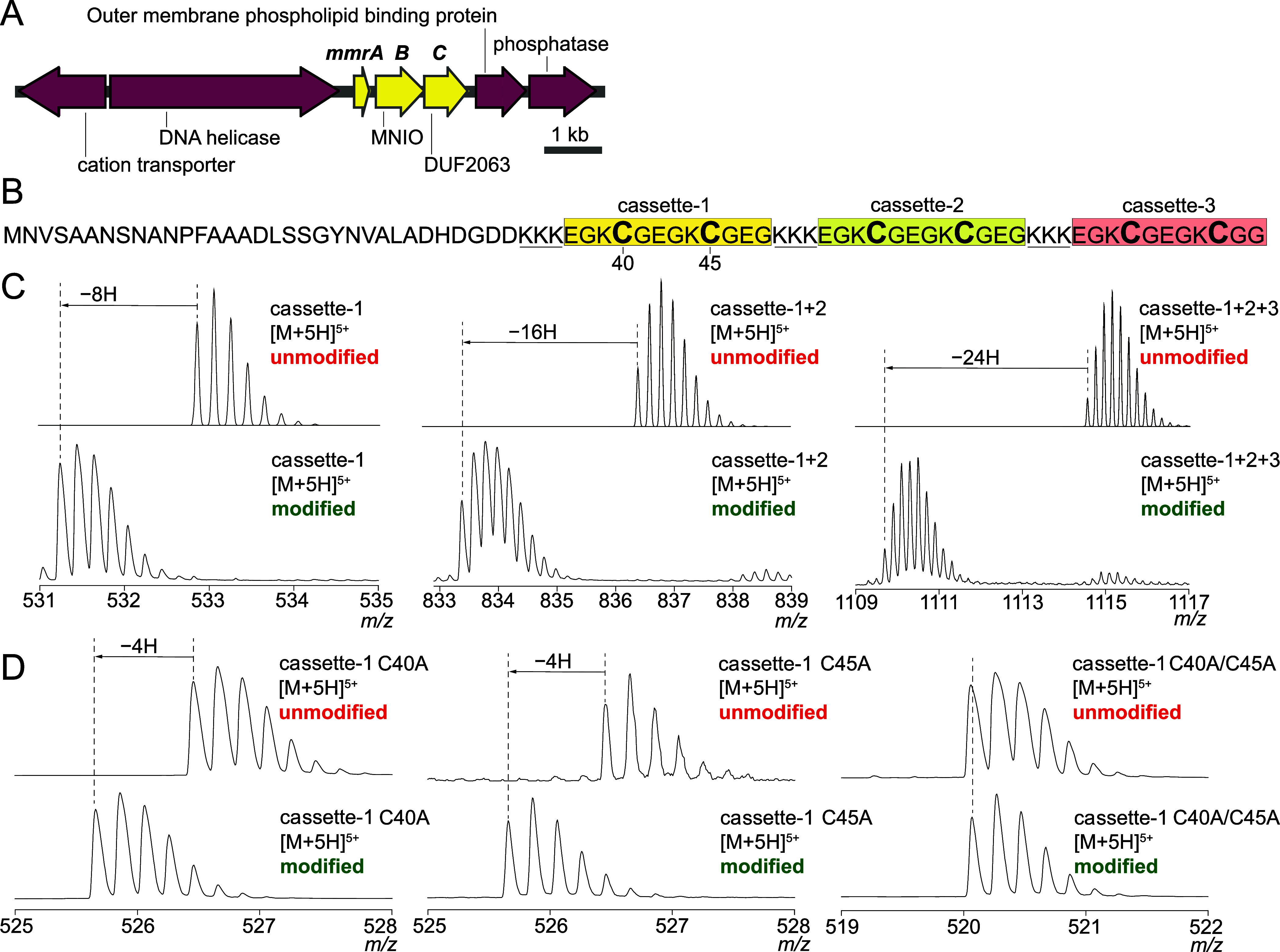
(A) The *mmr* BGC from *Microbulbifer* sp. VAAF005. The *mmrA–C* genes are colored yellow. Two ORFs at either end of the *mmrA–C* are annotated to demonstrate that they likely do not participate in RiPP biosynthesis. (B) MmrA primary sequence from *Microbulbifer* sp. VAAF005 illustrating the three-cassette architecture. Note that cassette-3 has a slight variation with one less Glu residue toward its C-terminus as compared to cassette-1 and cassette-2. This difference is also observed in other MmrA sequences (Figure S9). (C) (top) Theoretical [M + 5H]^5+^ mass spectra of unmodified MmrA peptides compared to (bottom) experimentally observed [M + 5H]^5+^ mass spectra of modified MmrA peptides demonstrating the loss of 8H, 16H, and 24H from one, two, and three cassettes, respectively. (D) (top) Experimental [M + 5H]^5+^ mass spectra of unmodified MmrA cassette-1 peptides with either, or both Cys40 and Cys45 residues mutated to Ala compared to the experimentally observed [M + 5H]^5+^ mass spectra of the respective peptides modified by MmrB/MmrC. Note that no mass loss is observed in the modified peptide when both Cys40 and Cys45 are mutated to Ala.

For further experimental interrogation, *mmr* BGC from the GS-I strain *Microbulbifer* sp. VAAF005 was chosen (Figure 4A). By sequence gazing, the *Microbulbifer* sp. VAAF005 MmrA core peptide was assessed to be organized into three cassettes containing two Cys residues each (Figure 4B). The three cassettes were flanked by a 3× Lys repeat at the N-termini. The sequence boundary between the MmrA leader and core regions was not immediately apparent, and no protease/peptide hydrolase was encoded in the vicinity of the *mmr* BGCs. At this point, it was unclear whether the MNIO/DUF2063 pair of MmrB and MmrC would modify all three MmrA cassettes. The *mmrA* gene, and genes encoding MmrA peptide variants with only one and with two cassettes were created and coexpressed with *mmrB* and *mmrC* genes in *Escherichia coli* (Table S3). When only the gene encoding MmrA cassette-1 was expressed together with *mmrB* and *mmrC* genes, as compared to the theoretical mass spectrum of the unmodified peptide, a mass decrease of 8.06 Da corresponding to 8H was observed (Table S4). When genes encoding two and all three cassettes of MmrA were expressed together with *mmrB* and *mmrC* genes, mass losses corresponding to 16H and 24H were observed, respectively (Figures 4C, S10–S12). These data suggested that all three MmrA cassettes were modified by MmrB/MmrC, and that each cassette, upon modification, suffered a loss of 8H. Within the MmrA cassette-1, when either of the two Cys residues—Cys40 and Cys45—were mutated to Ala, mass decreases of only 4H were observed upon peptide modification by MmrB/MmrC. When both Cys residues were concomitantly mutated to Ala, no change in mass was observed (Figures 4D, S13–S18, and Table S4). Taken together, these data imply that each of the six Cys residues spanning three cassettes in *Microbulbifer* sp. VAAF005 MmrA are modified by MmrB/MmrC and that each Cys modification corresponds to a 4H reduction in mass of the substrate peptide. Up to eight Cys residues are observed in MmrA precursor peptide sequences from the GS-III strain *Microbulbifer*, sp. ANSA002 and *Microbulbifer* sp. ANSA004 would conceivably be modified by the corresponding MmrB/MmrC complexes (Figure S9).

To further query the modifications installed by MmrB/MmrC, the modified MmrA cassette-1 peptide was digested with trypsin to remove the leader peptide, and a 12-mer peptide product purified with the molecular formula C_43_H_63_N_13_O_19_S_2_, as deduced by high resolution mass spectrometry, indicating 20 degrees of unsaturation. Consistent with the peptidic nature of the product, amide proton signals (δ_H_ 7.67–8.92) and amino acid Cα proton signals (δ_H_ 3.56–5.09) were observed in the 1D ^1^H NMR spectrum; amide carboxylic carbon signals (δ_C_ 165.4–174.0) and amino acid Cα carbon signals (δ_C_ 40.7–53.2) were observed in the 1D ^13^C­{^1^H}-NMR spectra (Table S5 and Figures S19 and S20). Analyses of the 2D ^1^H–^13^C HSQC, ^1^H–^13^C HMBC, ^1^H–^1^H COSY, ^1^H–^1^H TOCSY, and ^1^H–^1^H ROESY spectra allowed discerning the five Gly and three Glu residues (Figures S21–S25). The ^1^H–^13^C HSQC spectrum additionally revealed the disappearance of Cys40 and Cys45 Hα and Hβ atoms. This is in line with the above-mentioned mass spectrometry data indicating post-translational tailoring of Cys40 and Cys45 residues. Key ^1^H–^13^C HMBC correlations could be found from Hα (δ_H_ 4.04, 3.74; 3.92, 3.88) and NH (δ_H_ 8.51; 8.50) of the Gly41 and Gly46 to carbonyls (δ_C_ 159.2; 159.0) of Cys40 and Cys45, respectively. Additionally, Hα (δ_H_ 5.09; 5.00) of Lys39 and Lys44 showed clear ^1^H–^13^C HMBC correlations to their carbonyl carbons (δ_C_ 165.4; 166.4). These observations point toward the presence of either thiooxazole or oxazolone-thioamide motifs—isomeric moieties described in RiPPs that are biosynthesized by MNIO/DUF2063 complexes, among other enzymes—between Lys39/Cys40/Gly41 and Lys44/Cys45/Gly46 residues.^61–65^ Both possible structures bear proton-deficient five-membered heterocycles and theoretically match the observed HMBC correlations. Reduction by tris­(2-carboxyethyl)­phosphine (TCEP) established that one final degree of unsaturation could be satisfied by disulfide bond formation (Figure S26).

To differentiate between the thiooxazole and the oxazolone-thioamide possibilities, we compared the NMR chemical shifts of related RiPPs reported in the literature. Bufferins and SbtMa peptide are two RiPPs reported with thiooxazole motifs.^62,64^ For one of the two Cys residues that were modified to thiooxazoles in bufferins, the carbonyl carbon δ_C_ assignment has been revised to 168 ppm, which is similar to the Cys carbonyl δ_C_ assignment as 159 ppm; this shift was not reported for the SbtMa peptide (Table S6). However, for both oxazolone-thioamide-containing RiPPs oxazolin and Mov XBC peptide, the Cys carbonyl carbon δ_C_ was assigned as 165 ppm, which complicates the carbonyl/thiocarbonyl distinction.^63,65^ For methanobactin, from *Methylosinus* sp. LW4, the two Cys carbonyl carbon δ_C_ were assigned as 185 and 186 ppm; it should be noted that oxazolin and Mbn were chelated to Cu^+^.^61^ Hence, in a proton-deficient ring system with multiple quaternary carbon atoms, NMR chemical shifts alone were not sufficient for robust chemical assignment. These observations prompted us to look for other lines of evidence for clarification.

We first turned our attention to UV–vis absorbance spectra for these molecules. For the RiPPs oxazolin, Mov XBC peptide, bufferin, and SbtMa peptide, the UV absorbance maxima were reported as 302–305 nm. The MmrA-derived post-translationally modified 12-residue peptide demonstrated identical absorbance maxima in its reduced form (Figure S27 and Table S6). However, methanobactins have quite different UV absorbance maxima at 340 and 394 nm.^61,66,67^ Of note, the structure of the oxazolone-thioamide-containing methanobactin from *Methlyosinus trichosporium* OB3b has been confirmed by X-ray crystallography. Furthermore, it was observed that the oxazolone-thioamide motifs in the *M. trichosporium* OB3b-derived methanobactin were susceptible to acidic methanolysis (Figure S28).^66,67^ In concert with this observation, Mbn from *Methylosinus* sp. LW4 and the Mov XBC peptide—both of which were also reported to contain the oxazolone-thioamide motifs—also demonstrated hydrolysis and decarboxylation in acidic conditions. When the MmrA-derived peptidic product was acid treated for up to 72 h, no hydrolysis or decarboxylation products were detected. Taken together, differences in the UV absorbance and chemical degradation characteristics of the MmrA-derived peptidic product from that of the methanobactins allow us to tentatively assign the MmrB/MmrC-catalyzed post-translation modifications as thiooxazoles, which is in line with bufferins that are analogously constructed using a MNIO/DUF2063 enzyme pair.^64^ Due to the complexity of the spectroscopic signatures induced by their proton-deficient nature, structural revisions in the thiooxazole/oxazolone-thioamide class of RiPPs could be likely in the future. For clarity, the 12-residue MmrA-derived peptidic product is henceforth termed bulbicupramide (Figure 5). Six thiooxazole rings in the three MmrA cassettes are installed by MmrB and MmrC with the first and sixth Cys residues that are modified separated by 34 amino acids (Figure 4B). We did not detect the production of bulbicupramide in its oxidized or reduced forms under laboratory conditions for *Microbulbifer* cultivation.

**Figure 5:**
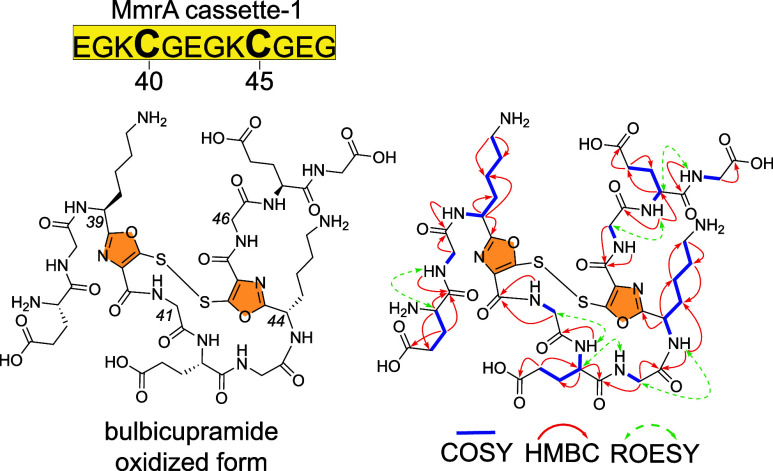
Structure and key NMR correlations for oxidized bulbicupramide. The thiooxazole rings are highlighted for clarity.

### Metal Binding by Bulbicupramide

Akin to methanobactins, thiooxazole and oxazolone-thioamide-containing RiPPs have been demonstrated to be metallophores.^64,65,68^ Hence, we investigated the metal binding characteristics of bulbicupramide in its reduced form. Using metal-infusion mass spectrometry, we discerned that reduced bulbicupramide readily bound Cu­(I). In contrast, reduced bulbicupramide did not appreciably bind Fe­(III), precluding its role as a siderophore (Figure 6A,B and S29–S31).^69^ Metal-infusion competition experiments, in which Cu­(I) was infused alongside trace elements Ni­(II), Zn­(II), and Mn­(II) revealed that bulbicupramide did not show appreciable binding to these metals either (Figures S32–S33).^69^ Taken together, these experiments allow us to annotate bulbicupramide, and by extension the post-translationally modified MmrA peptides, as chalkophore-like Cu­(I) binding RiPPs.

**Figure 6:**
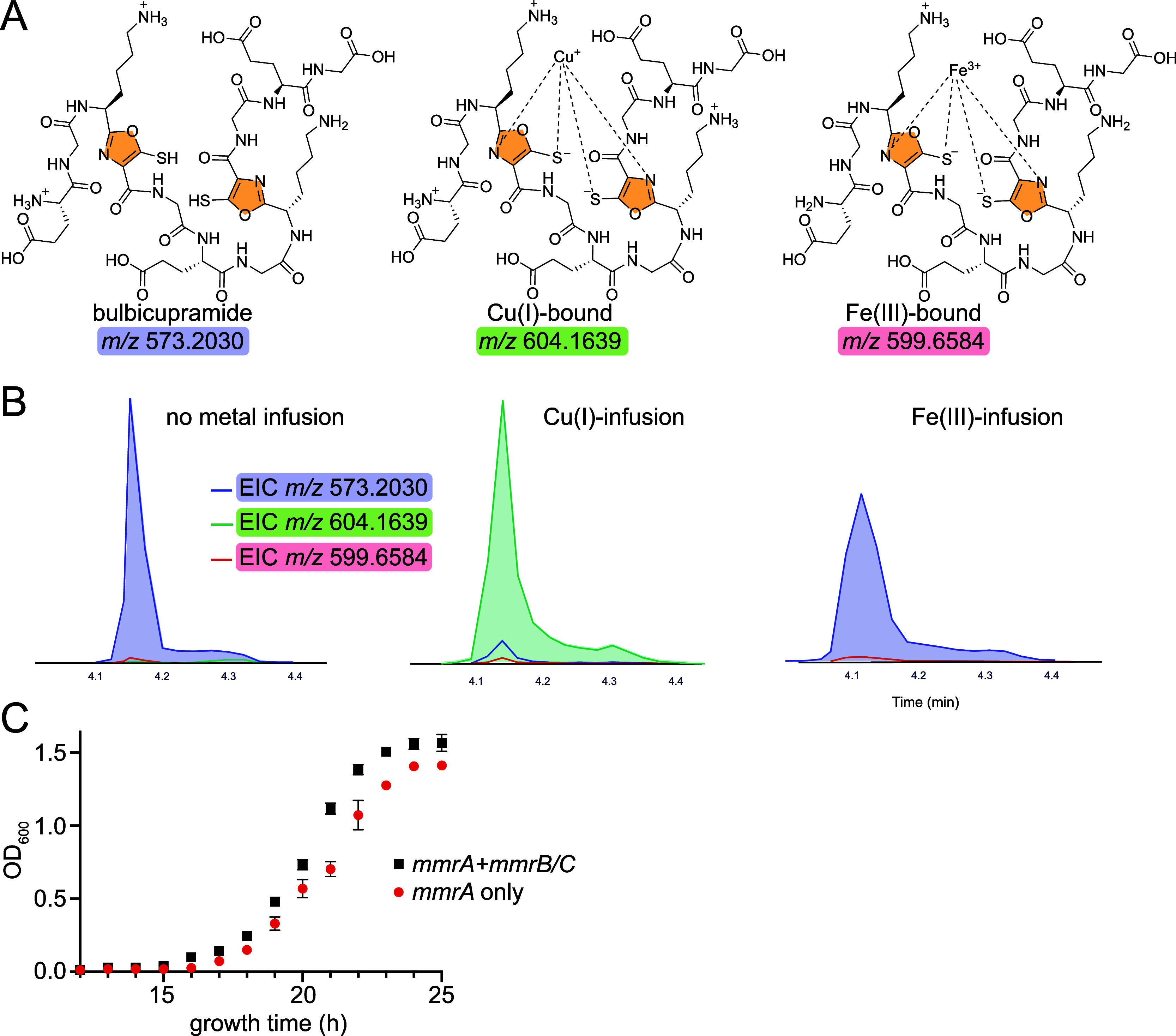
(A) Charged species of reduced bulbicupramide and in complex with Cu­(I) and Fe­(III). *m*/*z* are depicted with *z* = +2. (B) Extracted ion chromatograms (EICs) for reduced bulbicupramide and in complex with Cu­(I) and Fe­(III) when reduced bulbicupramide (left) was infused with (middle) Cu­(I) salt, and (right) with Fe­(III) salt. Note that upon infusion with Cu­(I), minimal amount of free bulbicupramide is detected in contrast to infusion with Fe­(III), wherein Fe­(III)-bound state is detected in much lower abundance as compared to the free state. EICs are plotted with a 10-ppm tolerance. (C) Coexpression of *mmrA* together with *mmrB* and *mmrC* (black data points) supports higher optical density and faster growth as compared to expression of *mmrA* alone (red data points) in liquid cultures of *E. coli* grown in the presence of 2 mM CuSO_4_. Means and standard deviations from triplicate experiments are plotted.

Unlike methanotrophic bacteria, *Microbulbifer* have no obvious nutritional requirement for acquiring high levels of copper. If *Microbulbifer* have no methanotroph-like requirement for copper acquisition, why do they conserve the *mmr* BGC in their genomes that leads to chalkophore-like Cu­(I) binding RiPP production? It is tantalizing to propose that rather than fulfilling a nutritional requirement for copper, *mmr*-encoded chalkophore RiPPs could have a protective role against copper-induced toxicity. In addition to the thiooxazole-containing bufferins, similar protective roles against metal toxicity have been proposed for cyanobacterial metallophores as well.^64,70^ There are no proteases encoded in the vicinity of the *mmr* BGC. It is thus plausible that the MmrA peptide after installation of the thiooxazoles would not be digested and that digestion products would not be secreted as small RiPPs; note that bulbicupramide or other related peptidic products are not detected in *Microbulbifer* extracts. Akin to bacterial cytosolic copper storage proteins that also confer protection against copper toxicity,^71^ the modified MmrA peptide could instead reside intracellularly or as a membrane bound protein. This assertion is in line with the recently reported maturation pathway of copper-chelating oxazolins in *Haemophilus influenzae* from a peptidic precursor that bears MmrA-like multiple di-Cys containing cassettes.^65^ A previous study had identified this peptide as a membrane bound virulence factor for pathogenic *H. influenzae* strains.^72^ Also noteworthy is the presence of N-terminal signal peptide signatures in bufferins that direct those RiPPs to the bacterial periplasm.^64^

To query this hypothesis, we examined the proteome of *Microbulbifer* sp. VAAF005 under routine laboratory growth conditions, and in conditions that would induce copper toxicity. The proteomic data sets were benchmarked by detection of the housekeeping proteins RecA, MreB, and glyceraldehyde-3-phosphate dehydrogenase (GAPDH) and used to query the expression of MmrA–C proteins. In the absence of exogenously added copper salts, we did not detect the MmrA–C peptides to be present in the bacterial proteome (Figure S34, Supporting Information, Data set D2). When culture media was supplemented with 0.1 mM and 0.2 mM CuSO_4_—conditions that induced production of bufferins^64^—we again did not detect the MmrA–C peptides in the bacterial proteomes (Supporting Information, Data set D3, and Supporting Information, Data set D4). These data demonstrate that the *mmrA–C* genes are not expressed in *Microbulbifer*, and that their expression is not responsive to copper stress. While *Microbulbifer* are commensal bacteria commonly associated with marine invertebrate holobionts, and their physiological growth conditions would thus likely be modulated by the eukaryotic host, it is unlikely that these bacteria would suffer from still higher copper stress in their native environment than what was applied here.

It is plausible that the exogenous addition of copper salts to growth media was not representative of the mechanism of copper exposure experienced by *Microbulbifer* in their native marine environment. Hence, an alternative mechanism to test the hypothesized protective activity of bulbicupramide was employed. Here, the gene *mmrA* was expressed in *E. coli*, with and without the concomitant expression of *mmrB* and *mmrC*. When MmrA was modified in vivo by MmrB and MmrC, we observed only a slight enhancement of *E. coli* growth kinetics and final liquid culture optical density in the presence of 2 mM CuSO_4_ (Figure 6C). In solid media, any observed enhancement of *E. coli* colony growth under copper stress when *mmrA* was coexpressed with *mmrB*/*mmrC* was not robust and instead highly dependent on experimental manipulations, such as inoculum dilution (Figure S35). It should also be noted that these experiments employed copper salts in millimolar concentrations, which is not physiologically relevant in the marine environment. Taken together, we are hesitant to assign a copper-toxicity protective role to the *mmr* BGC.

These observations call into question the functional relevance of conservation of the *mmr* BGC in *Microbulbifer* bacteria. Mining the genomes of other Proteobacterial strains that are isolated from commensal microbiomes of marine sponges—such as those from genera *Pseudovibrio* and *Ruegeria*—likewise reveals the presence of BGCs encoding the MNIO/DUF2063 polypeptide pairs in the vicinity of Cys-rich RiPP precursor peptides (Figure S36).^14,73^ At present, the lack of expression of the *mmr* genes in *Microbulbifer* and absence of definitive proof of an ecological role are juxtaposed against the widespread conservation of the genetic potential for the production of chalkophore-like Cu­(I) binding RiPPs in marine commensal microbiomes. Regardless, findings reported herein point toward the ubiquity of peptidic thiooxazole/oxazole-thioamide moieties in extending the capacity of ribosomally synthesized peptides and proteins as metallophores. The *Microbulbifer* genomes are otherwise replete with biosynthetic potential for siderophore production for Fe­(III) acquisition. Inventorying the siderophore encoding BGCs reveals that the GS-II strains encode the production of pyochelin-, aerobactin-, petrobactin-, and enterobactin-like siderophores (Figures S37–S41). As before, species-specific differences are apparent, with GS-III and GS-IV being restricted to only pyochelin- and aerobactin-like siderophore BGCs.

Findings from this study establish that in addition to the obligate symbiotic microbiome of sponges and the sponge eukaryotic host itself, RiPP biosynthetic loci are conserved in the commensal microbiomes as well. This conservation extends to the commensal microbiomes of other marine invertebrates such as corals.^7,52^ The RiPP chemical classes among the three holobiont constituents are different—the obligate symbiotic microbiome harbors BGCs that encode production of linear azol­(in)­e containing peptides,^52^ the sponge host furnishes proline-rich macrocyclic peptides,^7^ and we now show that the commensal microbiome encodes production of thiooxazole-containing chalkophore-like Cu­(I) binding RiPPs. The ecological roles of RiPPs are complex and often difficult to decipher.^74^ Functions of the three classes of RiPPs in marine holobiont physiology and intra- and interorganismal interactions remain to be deciphered.

The bioinformatic findings described in this study were developed as part of a pedagogic effort to introduce phylogenomics, genome mining, and natural product chemistry at the senior undergraduate level at the Georgia Institute of Technology. These efforts, in addition to other recently described initiatives that enhance literacy in chemical and biological sciences, advance microbial sciences and contribute to achieving sustainable development goals.^75,76^

## Supplementary Material











## Data Availability

The NMR data for bulbicupramide have been deposited to the Natural Product Magnetic Resonance Database (NP-MRD) with the accession no. NP0333790. The *Microbulbifer* genomes have been deposited to GenBank with the BioProject accession no. PRJNA1148949. Mass spectrometry data has been deposited to MassIVE with the accession nos. MSV000095600 and MSV000095853.

## Notes

Supplementary Dataset D1: ANI scores for *Microbulbifer* strains (XLSX)
Supplementary Dataset D2: Mass spectrometry-based proteomic abundance for *Microbulbifer* sp. VAAF005 grown in liquid media without exogenous addition of copper salts (XLSX)
Supplementary Dataset D3: Mass spectrometry-based proteomic abundance for *Microbulbifer* sp. VAAF005 grown in liquid media with exogenous addition of 0.1 mM copper sulfate (XLSX)
Supplementary Dataset D4: Mass spectrometry-based proteomic abundance for *Microbulbifer* sp. VAAF005 grown in liquid media with exogenous addition of 0.2 mM copper sulfate (XLSX)
Detailed description of Materials and Methods, Supplementary Tables S1–S5, Supplementary Figures S1–S40, and Supplementary References (PDF)

## References

[1] de
Goeij J. M., van Oevelen D., Vermeij M. J. A., Osinga R., Middelburg J. J., de Goeij A. F. P. M., Admiraal W. (2013). Surviving in a marine
desert: the sponge loop retains resources within coral reefs. Science.

[2] Knowlton, N. ; Brainard, R. E. ; Fisher, R. ; Moews, M. ; Plaisance, L. ; Caley, M. J. Coral Reef Biodiversity. In Life in the World’s Oceans, Wiley, 2010; pp 65–78.

[3] McCauley E. P., Piña I. C., Thompson A. D., Bashir K., Weinberg M., Kurz S. L., Crews P. (2020). Highlights of marine natural products
having parallel scaffolds found from marine-derived bacteria, sponges,
and tunicates. J. Antibiot..

[4] Morita M., Schmidt E. W. (2018). Parallel lives of symbionts and hosts:
chemical mutualism
in marine animals. Nat. Prod. Rep..

[5] Lackner G., Peters E. E., Helfrich E. J., Piel J. (2017). Insights into the lifestyle
of uncultured bacterial natural product factories associated with
marine sponges. Proc. Natl. Acad. Sci. U.S.A..

[6] Wilson K., de Rond T., Burkhardt I., Steele T. S., Schäfer R. J.
B., Podell S., Allen E. E., Moore B. S. (2023). Terpene biosynthesis
in marine sponge animals. Proc. Natl. Acad.
Sci. U.S.A..

[7] Lin Z., Agarwal V., Cong Y., Pomponi S. A., Schmidt E. W. (2024). Short macrocyclic
peptides in sponge genomes. Proc. Natl. Acad.
Sci. U.S.A..

[8] Scesa P. D., Lin Z., Schmidt E. W. (2022). Ancient
defensive terpene biosynthetic gene clusters
in the soft corals. Nat. Chem. Biol..

[9] Grayson N. E., Scesa P. D., Moore M. L., Ledoux J.-B., Gomez-Garrido J., Alioto T., Michael T. P., Burkhardt I., Schmidt E. W., Moore B. S. (2025). A widespread metabolic
gene cluster
family in metazoans. Nat. Chem. Biol..

[10] Loh T.-L., Pawlik J. R. (2014). Chemical defenses and resource trade-offs structure
sponge communities on Caribbean coral reefs. Proc. Natl. Acad. Sci. U.S.A..

[11] Puglisi M. P., Sneed J. M., Sharp K. H., Ritson-Williams R., Paul V. J. (2014). Marine chemical ecology in benthic environments. Nat. Prod. Rep..

[12] Dat T. T.
H., Steinert G., Cuc N. T. K., Smidt H., Sipkema D. (2021). Bacteria Cultivated
From Sponges and Bacteria Not Yet Cultivated From Sponges—A
Review. Front. Microbiol..

[13] Dieterich C. L., Probst S. I., Ueoka R., Sandu I., Schäfle D., Molin M. D., Minas H. A., Costa R., Oxenius A., Sander P., Piel J. (2022). Aquimarins, Peptide Antibiotics with
Amino-Modified C-Termini from a Sponge-Derived *Aquimarina* sp. Bacterium. Angew. Chem., Int. Ed..

[14] Ióca L. P., Dai Y., Kunakom S., Diaz-Espinosa J., Krunic A., Crnkovic C. M., Orjala J., Sanchez L. M., Ferreira A. G., Berlinck R. G. S., Eustáquio A. S. (2021). A family
of nonribosomal peptides
modulate collective behavior in *Pseudovibrio* bacteria
isolated from marine sponges. Angew. Chem.,
Int. Ed..

[15] Lu S., Zhang Z., Sharma A. R., Nakajima-Shimada J., Harunari E., Oku N., Trianto A., Igarashi Y. (2023). Bulbiferamide,
an antitrypanosomal hexapeptide cyclized via an N-acylindole linkage
from a marine obligate *Microbulbifer*. J. Nat. Prod..

[16] Zhong W., Aiosa N., Deutsch J. M., Garg N., Agarwal V. (2023). Pseudobulbiferamides:
plasmid-encoded ureidopeptide natural products with biosynthetic gene
clusters shared among marine bacteria of different genera. J. Nat. Prod..

[17] Zhong W., Deutsch J. M., Yi D., Abrahamse N. H., Mohanty I., Moore S. G., McShan A. C., Garg N., Agarwal V. (2023). Discovery and biosynthesis of ureidopeptide natural
products macrocyclized via indole N-acylation in marine *Microbulbifer* spp. bacteria. ChemBioChem.

[18] Zhong W., Agarwal V. (2024). Polymer degrading marine *Microbulbifer* bacteria: an un­(der)­utilized source of chemical and biocatalytic
novelty. Beilstein J. Org. Chem..

[19] Zhong W., Budimir Z. L., Johnson L. O., Parkinson E. I., Agarwal V. (2024). Activity and Biocatalytic Potential of an Indolylamide
Generating Thioesterase. Org. Lett..

[20] Maruyama H., Yamada Y., Igarashi Y., Matsuda K., Wakimoto T. (2025). Enzymatic
peptide macrocyclization via indole-N-acylation. Chem. Sci..

[21] Deutsch J. M., Green M. O., Akavaram P., Davis A. C., Diskalkar S. S., Du Plessis I. A., Fallon H. A., Grason E. M., Kauf E. G., Kim Z. M., Miller J. R., Neal A. L., Riera T., Stroeva S.-E., Tran J., Tran V., Coronado A. V., Coronado V. V., Wall B. T., Yang C. M., Mohanty I., Abrahamse N. H., Freeman C. J., Easson C. G., Fiore C. L., Onstine A. E., Djeddar N., Biliya S., Bryksin A. V., Garg N., Agarwal V. (2023). Limited metabolomic overlap between
commensal bacteria and marine sponge holobionts revealed by large
scale culturing and mass spectrometry-based metabolomics: an undergraduate
laboratory pedagogical effort at Georgia Tech. Mar. Drugs.

[22] Becher P. G., Verschut V., Bibb M. J., Bush M. J., Molnár B. P., Barane E., Al-Bassam M. M., Chandra G., Song L., Challis G. L., Buttner M. J., Flärdh K. (2020). Developmentally
regulated volatiles geosmin and 2-methylisoborneol attract a soil
arthropod to *Streptomyces* bacteria promoting spore
dispersal. Nat. Microbiol..

[23] Shi Y.-M., Hirschmann M., Shi Y.-N., Ahmed S., Abebew D., Tobias N. J., Grün P., Crames J. J., Pöschel L., Kuttenlochner W., Richter C., Herrmann J., Müller R., Thanwisai A., Pidot S. J., Stinear T. P., Groll M., Kim Y., Bode H. B. (2022). Global analysis of biosynthetic gene clusters reveals
conserved and unique natural products in entomopathogenic nematode-symbiotic
bacteria. Nat. Chem..

[24] Salamzade R., Kalan L. R. (2025). Context matters: assessing the impacts
of genomic background
and ecology on microbial biosynthetic gene cluster evolution. mSystems.

[25] Meinwald J., Eisner T. (2008). Chemical ecology in retrospect and prospect. Proc. Natl. Acad. Sci. U.S.A..

[26] van
Bergeijk D. A., Terlouw B. R., Medema M. H., van Wezel G. P. (2020). Ecology
and genomics of Actinobacteria: new concepts for natural product discovery. Nat. Rev. Microbiol..

[27] Montalbán-López M., Scott T. A., Ramesh S., Rahman I. R., van Heel A. J., Viel J. H., Bandarian V., Dittmann E., Genilloud O., Goto Y., Grande Burgos M. J., Hill C., Kim S., Koehnke J., Latham J. A., Link A. J., Martínez B., Nair S. K., Nicolet Y., Rebuffat S., Sahl H.-G., Sareen D., Schmidt E. W., Schmitt L., Severinov K., Süssmuth R. D., Truman A. W., Wang H., Weng J.-K., van Wezel G. P., Zhang Q., Zhong J., Piel J., Mitchell D. A., Kuipers O. P., van der Donk W. A. (2021). New developments
in RiPP discovery, enzymology and engineering. Nat. Prod. Rep..

[28] Mohanty I., Tapadar S., Moore S. G., Biggs J. S., Freeman C. J., Gaul D. A., Garg N., Agarwal V. (2021). Presence of bromotyrosine
alkaloids in marine sponges is independent of metabolomic and microbiome
architectures. mSystems.

[29] Minh B. Q., Schmidt H. A., Chernomor O., Schrempf D., Woodhams M. D., von Haeseler A., Lanfear R. (2020). IQ-TREE 2: New Models and Efficient
Methods for Phylogenetic Inference in the Genomic Era. Mol. Biol. Evol..

[30] Katoh K., Rozewicki J., Yamada K. D. (2019). MAFFT online service: multiple sequence
alignment, interactive sequence choice and visualization. Briefings in Bioinformatics.

[31] Gevers D., Cohan F. M., Lawrence J. G., Spratt B. G., Coenye T., Feil E. J., Stackebrandt E., De Peer Y. V., Vandamme P., Thompson F. L., Swings J. (2005). Re-evaluating prokaryotic species. Nat. Rev. Microbiol..

[32] Guindon S., Dufayard J. F., Lefort V., Anisimova M., Hordijk W., Gascuel O. (2010). New algorithms and
methods to estimate
maximum-likelihood phylogenies: assessing the performance of PhyML
3.0. Syst. Biol..

[33] Minh B. Q., Nguyen M. A., von Haeseler A. (2013). Ultrafast
approximation for phylogenetic
bootstrap. Mol. Biol. Evol..

[34] Hoang D. T., Chernomor O., von Haeseler A., Minh B. Q., Vinh L. S. (2018). UFBoot2:
Improving the Ultrafast Bootstrap Approximation. Mol. Biol. Evol..

[35] Chan J. Z. M., Halachev M. R., Loman N. J., Constantinidou C., Pallen M. J. (2012). Defining bacterial species in the
genomic era: insights
from the genus *Acinetobacter*. BMC Microbiology.

[36] Nishijima M., Takadera T., Imamura N., Kasai H., An K.-D., Adachi K., Nagao T., Sano H., Yamasato K. (2009). *Microbulbifer
variabilis* sp. nov. and *Microbulbifer epialgicus* sp. nov., isolated from Pacific marine algae, possess a rod–coccus
cell cycle in association with the growth phase. Int. J. Syst. Evol. Microbiol..

[37] Goris J., Konstantinidis K. T., Klappenbach J. A., Coenye T., Vandamme P., Tiedje J. M. (2007). DNA–DNA hybridization values and their relationship
to whole-genome sequence similarities. Int.
J. Syst. Evol. Microbiol..

[38] Konstantinidis K. T., Tiedje J. M. (2005). Genomic insights
that advance the species definition
for prokaryotes. Proc. Natl. Acad. Sci. U.S.A..

[39] Jain C., Rodriguez-R L. M., Phillippy A. M., Konstantinidis K. T., Aluru S. (2018). High throughput ANI analysis of 90K
prokaryotic genomes reveals clear
species boundaries. Nat. Commun..

[40] Richter M., Rosselló-Móra R. (2009). Shifting the
genomic gold standard
for the prokaryotic species definition. Proc.
Natl. Acad. Sci. U.S.A..

[41] Blin K., Shaw S., Kloosterman A. M., Charlop-Powers Z., van Wezel G. P., Medema M., Weber T. (2021). antiSMASH 6.0: improving
cluster detection and comparison capabilities. Nucleic Acids Res..

[42] Naughton L. M., Romano S., O’Gara F., Dobson A. D. W. (2017). Identification
of Secondary Metabolite Gene Clusters in the *Pseudovibrio* Genus Reveals Encouraging Biosynthetic Potential toward the Production
of Novel Bioactive Compounds. Front. Microbiol..

[43] Buijs Y., Bech P. K., Vazquez-Albacete D., Bentzon-Tilia M., Sonnenschein E. C., Gram L., Zhang S.-D. (2019). Marine
Proteobacteria
as a source of natural products: advances in molecular tools and strategies. Nat. Prod. Rep..

[44] Wang J., Li P., Di X., Lu H., Wei H., Zhi S., Fewer D. P., He S., Liu L. (2024). Phylogenomic analysis
uncovers an unexpected capacity for the biosynthesis of secondary
metabolites in *Pseudoalteromonas*. Eur. J. Med. Chem..

[45] Wei B., Hu G.-A., Zhou Z.-Y., Yu W.-C., Du A.-Q., Yang C.-L., Yu Y.-L., Chen J.-W., Zhang H.-W., Wu Q., Xuan Q., Xu X.-W., Wang H. (2023). Global analysis of
the biosynthetic chemical space of marine prokaryotes. Microbiome.

[46] Navarro-Muñoz J. C., Selem-Mojica N., Mullowney M. W., Kautsar S. A., Tryon J. H., Parkinson E. I., De Los Santos E. L. C., Yeong M., Cruz-Morales P., Abubucker S., Roeters A., Lokhorst W., Fernandez-Guerra A., Cappelini L. T. D., Goering A. W., Thomson R. J., Metcalf W. W., Kelleher N. L., Barona-Gomez F., Medema M. H. (2020). A computational
framework to explore large-scale biosynthetic diversity. Nat. Chem. Biol..

[47] Mavrodi D. V., Peever T. L., Mavrodi O. V., Parejko J. A., Raaijmakers J. M., Lemanceau P., Mazurier S., Heide L., Blankenfeldt W., Weller D. M., Thomashow L. S. (2010). Diversity and Evolution of the Phenazine
Biosynthesis Pathway. Appl. Environ. Microbiol..

[48] Won N. I., Lee G. E., Ko K., Oh D. C., Na Y. H., Park J. S. (2017). Identification of a Bioactive Compound,
Violacein,
from *Microbulbifer* sp. Isolated from a Marine Sponge *Hymeniacidon sinapium* on the West Coast of Korea. Microbiol. Biotechnol. Lett..

[49] Enomoto, K. Violacein and Prodiginines from Marine Bacteria. In Encyclopedia of Marine Biotechnology, Wiley, 2020; pp 1689–1710.

[50] Schalk I. J. (2025). Bacterial
siderophores: diversity, uptake pathways and applications. Nat. Rev. Microbiol..

[51] Wang K., Cui B., Wang Y., Luo W. (2025). Microbial Production of Ectoine:
A Review. ACS Synth. Biol..

[52] Nguyen N. A., Lin Z., Mohanty I., Garg N., Schmidt E. W., Agarwal V. (2021). An obligate
peptidyl brominase underlies the discovery of highly distributed biosynthetic
gene clusters in marine sponge microbiomes. J. Am. Chem. Soc..

[53] Nowak V. V., Hou P., Owen J. G. (2024). Microbial
communities associated with marine sponges
from diverse geographic locations harbor biosynthetic novelty. Appl. Environ. Microbiol..

[54] Chen J. Y., van der Donk W. A. (2024). Multinuclear non-heme iron dependent oxidative enzymes
(MNIOs) involved in unusual peptide modifications. Curr. Opin. Chem. Biol..

[55] Kenney G. E., Dassama L. M. K., Pandelia M.-E., Gizzi A. S., Martinie R. J., Gao P., DeHart C. J., Schachner L. F., Skinner O. S., Ro S. Y., Zhu X., Sadek M., Thomas P. M., Almo S. C., Bollinger J. M., Krebs C., Kelleher N. L., Rosenzweig A. C. (2018). The biosynthesis
of methanobactin. Science.

[56] Dou C., Long Z., Li S., Zhou D., Jin Y., Zhang L., Zhang X., Zheng Y., Li L., Zhu X., Liu Z., He S., Yan W., Yang L., Xiong J., Fu X., Qi S., Ren H., Chen S., Dai L., Wang B., Cheng W. (2022). Crystal structure
and catalytic mechanism of the MbnBC holoenzyme required for methanobactin
biosynthesis. Cell Research.

[57] Park Y. J., Jodts R. J., Slater J. W., Reyes R. M., Winton V. J., Montaser R. A., Thomas P. M., Dowdle W. B., Ruiz A., Kelleher N. L., Bollinger J. M., Krebs C., Hoffman B. M., Rosenzweig A. C. (2022). A mixed-valent
Fe­(II)­Fe­(III) species converts cysteine
to an oxazolone/thioamide pair in methanobactin biosynthesis. Proc. Natl. Acad. Sci. U.S.A..

[58] Donia M. S., Ravel J., Schmidt E. W. (2008). A global assembly line for cyanobactins. Nat. Chem. Biol..

[59] Zhang Y., Li K., Yang G., McBride J. L., Bruner S. D., Ding Y. (2018). A distributive
peptide cyclase processes multiple microviridin core peptides within
a single polypeptide substrate. Nat. Commun..

[60] Lima S. T., Ampolini B. G., Underwood E. B., Graf T. N., Earp C. E., Khedi I. C., Pasquale M. A., Chekan J. R. (2023). A Widely Distributed
Biosynthetic Cassette Is Responsible for Diverse Plant Side Chain
Cross-Linked Cyclopeptides. Angew. Chem., Int.
Ed..

[61] Kenney G. E., Goering A. W., Ross M. O., DeHart C. J., Thomas P. M., Hoffman B. M., Kelleher N. L., Rosenzweig A. C. (2016). Characterization
of Methanobactin from Methylosinus sp. LW4. J. Am. Chem. Soc..

[62] Lewis J. K., Jochimsen A. S., Lefave S. J., Young A. P., Kincannon W. M., Roberts A. G., Kieber-Emmons M. T., Bandarian V. (2021). New Role for
Radical SAM Enzymes in the Biosynthesis of Thio­(seleno)­oxazole RiPP
Natural Products. Biochemistry.

[63] Chioti V. T., Clark K. A., Ganley J. G., Han E. J., Seyedsayamdost M. R. (2024). N-Calpha
Bond Cleavage Catalyzed by a Multinuclear Iron Oxygenase from a Divergent
Methanobactin-like RiPP Gene Cluster. J. Am.
Chem. Soc..

[64] Leprevost L., Jünger S., Lippens G., Guillaume C., Sicoli G., Oliveira L., Falcone E., de Santis E., Rivera-Millot A., Billon G., Stellato F., Henry C., Antoine R., Zirah S., Dubiley S., Li Y., Jacob-Dubuisson F. (2024). A widespread
family of ribosomal peptide metallophores
involved in bacterial adaptation to metal stress. Proc. Natl. Acad. Sci. U.S.A..

[65] Manley O. M., Shriver T. J., Xu T., Melendrez I. A., Palacios P., Robson S. A., Guo Y., Kelleher N. L., Ziarek J. J., Rosenzweig A. C. (2024). A multi-iron enzyme installs copper-binding
oxazolone/thioamide pairs on a nontypeable *Haemophilus influenzae* virulence factor. Proc. Natl. Acad. Sci. U.S.A..

[66] Behling L. A., Hartsel S. C., Lewis D. E., DiSpirito A. A., Choi D. W., Masterson L. R., Veglia G., Gallagher W. H. (2008). NMR, Mass
Spectrometry and Chemical Evidence Reveal a Different Chemical Structure
for Methanobactin That Contains Oxazolone Rings. J. Am. Chem. Soc..

[67] Kim H. J., Graham D. W., DiSpirito A. A., Alterman M. A., Galeva N., Larive C. K., Asunskis D., Sherwood P. M. (2004). Methanobactin, a
copper-acquisition compound from methane-oxidizing bacteria. Science.

[68] Reyes R. M., Rosenzweig A. C. (2024). Methanobactins:
Structures, Biosynthesis, and Microbial
Diversity. Annu. Rev. Microbiol..

[69] Aron A. T., Petras D., Schmid R., Gauglitz J. M., Büttel I., Antelo L., Zhi H., Nuccio S.-P., Saak C. C., Malarney K. P., Thines E., Dutton R. J., Aluwihare L. I., Raffatellu M., Dorrestein P. C. (2022). Native mass spectrometry-based metabolomics
identifies metal-binding compounds. Nat. Chem..

[70] Avalon N. E., Reis M. A., Thornburg C. C., Williamson R. T., Petras D., Aron A. T., Neuhaus G. F., Al-Hindy M., Mitrevska J., Ferreira L., Morais J., El Abiead Y., Glukhov E., Alexander K. L., Vulpanovici F. A., Bertin M. J., Whitner S., Choi H., Spengler G., Blinov K., Almohammadi A. M., Shaala L. A., Kew W. R., Paša-Tolić L., Youssef D. T. A., Dorrestein P. C., Vasconcelos V., Gerwick L., McPhail K. L., Gerwick W. H. (2024). Leptochelins
A–C, Cytotoxic Metallophores Produced by Geographically Dispersed *Leptothoe* Strains of Marine Cyanobacteria. J. Am. Chem. Soc..

[71] Vita N., Landolfi G., Baslé A., Platsaki S., Lee J., Waldron K. J., Dennison C. (2016). Bacterial cytosolic proteins with
a high capacity for Cu­(I) that protect against copper toxicity. Sci. Rep..

[72] Ahearn C. P., Kirkham C., Chaves L. D., Kong Y., Pettigrew M. M., Murphy T. F. (2019). Discovery and Contribution
of Nontypeable *Haemophilus
influenzae* NTHI1441 to Human Respiratory Epithelial Cell
Invasion. Infect. Immun..

[73] Han, S.-M. ; Park, J.-S. *Ruegeria spongiae* sp. nov., isolated from *Callyspongia elongata* . Int. J. Syst. Evol. Microbiol. 2023, 73. 10.1099/ijsem.0.006001. 37560994

[74] Li Y., Rebuffat S. (2020). The manifold roles
of microbial ribosomal peptide–based
natural products in physiology and ecology. J. Biol. Chem..

[75] Crowther T. W., Rappuoli R., Corinaldesi C., Danovaro R., Donohue T. J., Huisman J., Stein L. Y., Timmis J. K., Timmis K., Anderson M. Z., Bakken L. R., Baylis M., Behrenfeld M. J., Boyd P. W., Brettell I., Cavicchioli R., Delavaux C. S., Foreman C. M., Jansson J. K., Koskella B., Milligan-McClellan K., North J. A., Peterson D., Pizza M., Ramos J. L., Reay D., Remais J. V., Rich V. I., Ripple W. J., Singh B. K., Smith G. R., Stewart F. J., Sullivan M. B., van den Hoogen J., van Oppen M. J. H., Webster N. S., Zohner C. M., van Galen L. G. (2024). Scientists’
call to action: Microbes, planetary health, and the Sustainable Development
Goals. Cell.

[76] Moore B. S., Newman D. J. (2025). The Extraordinary Benefit of Nature’s
Chemistry
to Health, Society, and the Economy. J. Nat.
Prod..

