# Supplementary material for: Phylogenomic Identification of a Highly Conserved Copper-Binding RiPP Biosynthetic Gene Cluster in Marine Microbulbifer Bacteria
Source: ACS Chem Biol. 2025 Sep 19;20(10):2462–74. doi: 10.1021/acschembio.5c00507 (PMC12538549; doi:10.1021/acschembio.5c00507)
Supplement: Supplementary file 5 [file cb5c00507_si_005.pdf]

SUPPLEMENTARY INFORMATION FOR:

Phylogenomic identification of a highly conserved copper-binding RiPP biosynthetic gene cluster in marine *Microbulbifer* bacteria

Yifan Tang,[1,†] Weimao Zhong,[1,†] Longping Fu,[1] Emmanuel Asante,[2] Anastasiia Kostenko,[2] FNU Vidya,[1] Paige Mandelare-Ruiz,[3] Tamilore T. Adeogun,[4,§] Gabriel P. Anderson,[4,§] Benjamin E. Edmonds,[4,§] Oscar Fang,[4,§] Michelle Han,[4,§] Alia S. Hollingsworth,[4,§] Amna R. Ingham,[4,§] Carlyn R. Kirby,[4,§] Alice Landrum,[4,§] Connor R. Mack,[4,§] Nikki S. Nobari,[4,§] Emma J. Oswald,[4,§] Cecilia L. Polevoy,[4,§] Yasmin Sharifian,[4,§] Timothy J. So,[4,§] Joelee R. Stokes,[4,§] Reniya S. Thompson,[4,§] Rishabh Vuthamaraju,[4,§] Elaine C. Wang,[4,§] William H. Yang,[4,§] Alison E. Onstine,[4] Valerie J. Paul,[3] Ronghu Wu,[1] Allegra T. Aron,[2] Vinayak Agarwal[1,4,*]

[1] School of Chemistry and Biochemistry, Georgia Institute of Technology, Atlanta, GA 30332, USA

[2] Department of Chemistry and Biochemistry, University of Denver, Denver, CO 80210, USA

[3] Smithsonian Marine Station, Ft Pierce, FL 34949, USA

[4] School of Biological Sciences, Georgia Institute of Technology, Atlanta, GA 30332, USA

[†] Equal contribution first authors

[§] Equal contribution authors arranged per last name

* correspondence: vagarwal@gatech.edu

Supplementary Materials and Methods

General materials and instrumentation

All chemicals, solvents, and media components were obtained commercially from Sigma-Aldrich, Fisher Scientific, and VWR, and used without further purification. Phusion high-fidelity DNA polymerase and Gibson assembly Master Mix were purchased from New England Biolabs. PrimeSTAR DNA polymerase Master Mix was purchased from Takaro Bio. Mass spectra were recorded on an Agilent 6530C high resolution time of flight (ToF) mass spectrometer with an electrospray ionization (ESI) source coupled to an Agilent 1260 high-performance liquid chromatography system equipped with a diode array detector. Optical density at 600 nm wavelength (OD_{600}) was measured using Ultrospec 10 cell density meter.

Genomic DNA isolation and sequencing of *Microbulbifer* strains

From glycerol stocks of *Microbulbifer* sp. strains stored at −80 °C were inoculated 250 mL baffled shake flasks each containing 50 mL of half strength marine broth (MB½) liquid media and incubated at 30 °C for 3 d with shaking. The cell pellets were harvested by centrifugation and DNA isolation was performed using the Wizard Genomic DNA Purification kit using manufacturer's recommended protocol. Purified genomic DNA were stored at 4 °C prior to gel electrophoresis and Qubit concentration measurements. Samples were run on 0.5% agarose gels at 120 V for 50 min to check the integrity of the genomic DNA. Then, DNA concentration was measured using 1 µL of each sample using the Qubit High Resolution double stranded DNA modality. Samples were sequenced at Plasmidsaurus. Sequencing result files were inspected to ensure high quality sequencing quality. All genomic DNA sequenced had more than 30× coverage.

Phylogenetic tree construction

16S rRNA gene sequences were extracted with Barrnap 0.9[1] and aligned with Mafft version 7.[2] The phylogenetic trees were constructed with IQ-TREE2.[3] The analysis was run on 4 threads (*-nt 4*) with automatic ModelFinder[4] (*-m MFP*). Branch support was assessed through 1,000 ultrafast bootstrap replicates[5,6] (*-B 1000*) along with 1,000 replicates of the Shimodaira-Hasegawa approximate Likelihood Ratio Test (SH-aLRT)[7] (*-alrt 1000*). Outgroups[8] were specified as *Aeromonas hydrophila* ATCC 7966, *Escherichia coli* ATCC 11775, and *Vibrio cholerae* ATCC 14035 (*-o A_hydrophila_ATCC_7966, E_coli_ATCC_11775, V_cholerae_ATCC_14035*). The consensus phylogeny was visualized with FigTree v1.4.4.[9] An automated MLSA pipeline, automlsa2,[10] was employed to extract, align, and concatenate 15

housekeeping genes and generate a maximum likelihood tree. The consensus phylogeny was visualized with FigTree v1.4.4.[9]

Average Nucleotide Identity (ANI) heatmap representation

Pangenome was created using PanTools.[11] ANI similarity indices of the pangenome were calculated using FastANI[12] and MASH[13] to extract orthologous genes and produce a Rscript which constructed a neighborhood joining tree. Heatmap representation of the pairwise similarity index was visualized using seaborn.[14] The consensus phylogeny was visualized with FigTree v1.4.4.[9]

Gene Cluster Family (GCF) extraction

The *Microbulbifer* genomes were mined using antiSMASH 7.0.0 to predict genetic loci that encode production of secondary metabolites. The BGCs predicted from antiSMASH were networked into GCFs found across multiple strains using BiG-SCAPE.[15] All default settings were maintained during the BiG-SCAPE analysis, except that the cutoff value was adjusted to 0.5 instead of 0.3. To construct the GCF presence/absence matrix, three comma-separated values (CSV) files were prepared. The first file mapped the bacterial strains to their associated GCFs. This file contained two columns: column 1 listed the strain names, and column 2 listed the corresponding GCF numbers (1 through n) for each strain. Strains with multiple GCFs had their GCF numbers separated by commas. The second CSV file served as a blank matrix template with x columns and y rows. The first column listed the bacterial strains used in the MLSA and GCF analyses, ordered according to MLSA. The first row contained column headers and were labeled with numbers 1–n, representing gene cluster families (GCFs) 1–n. The remaining cells were initially blank. A Python script was then used to generate the third CSV file—the completed GCF presence/absence matrix. The script iterated over each strain in the second CSV file and populated the first CSV file by marking a '1' in the appropriate GCF columns if the strain possessed that GCF. Cells remained '0' if the GCF was absent. This process resulted in a matrix indicating the presence ('1') or absence ('0') of each GCF in each bacterial strain. Matplotlib was used to generate a dot plot from the presence/absence matrix, visually representing the distribution of GCFs across all bacterial strains.

Plasmid vector construction

Plasmids used in this study were constructed using standard molecular biology techniques. Amplification of target DNA fragments were carried out with either Phusion or PrimeSTAR high-fidelity DNA polymerases. Gibson assembly was used to subclone genes encoding for proteins of interest into the target vectors. Sequences of recombinant plasmids were confirmed by Sanger sequencing at Eton Bioscience or nanopore sequencing at Plasmidsaurus.

The DNA fragments encoding MmrA cassettes-1+2+3, MmrA cassettes-1+2, and MmrA cassette-1 were amplified from the genomic DNA of *Microbulbifer* sp. VAAF005 with Phusion high fidelity DNA polymerase. The amplified DNA fragments were inserted into the pET28(+) vector using Gibson assembly to furnish N-His$_6$ tag fused constructs. Plasmids for three mutants pET28_MmrA cassette-1 (C40A, C45A, and C40A/C45A) were generated by site-directed mutagenesis using standard procedures. The MmrA peptide sequences are available in Table S3.

The DNA fragments encoding MmrB and MmrC were amplified from the genomic DNA of *Microbulbifer* sp. VAAF005 with Phusion high fidelity DNA polymerase. The amplified DNA fragments were inserted into the MCS1 and MCS2 of pCDF-Duet vector, respectively, to furnish the expression plasmid.

Protein expression and purification

Plasmids pET28_*mmrA cassettes-1+2+3*, pET28_*mmrA cassettes-1+2*, and pET28_*mmrA cassette-1*, and three mutants pET28_*mmrA cassette-1* (C40A, C45A, and C40A/C45A) were co-transformed with pCDF-Duet_*mmrB/mmrC* plasmid into *Escherichia coli* BL21Gold(DE3) for protein expression. For furnishing unmodified peptide controls, the abovementioned *mmrA* expression plasmids were co-transformed with empty pCDF-Duet vector. 10 mL of overnight culture was inoculated into 1 L terrific broth supplemented with kanamycin (50 μg/L, final concentration) and streptomycin (50 μg/L). The cells were grown at 30 ˚C until OD$_{600}$ reached 0.6–0.7. Incubation temperature was then reduced to 18 ˚C. Protein expression was induced by the addition of 0.3 mM IPTG. Induced cultures were allowed to grow at 18 ˚C for an addition of 18 h before being harvested by centrifugation. Cell pellets were resuspended in 30 mL binding buffer (20 mM Tris-Cl (pH 7.9), 500 mM NaCl) and lysed by homogenization. The lysate was clarified by centrifugation (27,000×g, 45 min, 4 ℃), and then applied to a 5 mL HisTrap HP column. The column was washed with 10 column volumes of wash buffer (20 mM Tris-Cl (pH 7.9), 500 mM NaCl, 30 mM imidazole) and bound proteins were eluted using ÄKTAprime plus FPLC system with a linear gradient to 100% of elution buffer (20 mM Tris-Cl (pH 7.9), 500 mM NaCl, 250 mM imidazole) over 6

column volumes. Purity of eluent protein fractions was checked by denaturing gel electrophoresis. The fractions containing desired protein were pooled and desalted by PD-10 column chromatography into abovementioned binding buffer.

Proteolytic digestion of MmrA peptide and LC/MS-based detection

Enzymatic assays were performed at 30 ˚C for 3 h, in a reaction volume of 500 µL containing 50 mM Tris-Cl (pH 7.5), 10 µg/mL chymotrypsin or trypsin, and 50 µM MmrA peptides. Reactions were quenched by addition of equal volume of MeOH supplemented with 2% (v/v) formic acid (FA). Quenched aliquots were centrifuged at 18,000×g for 30 min at room temperature before analysis by LC/MS. Chromatographic separations were performed using Agilent Poroshell 120 EC-C_8 (for chymotrypsin-digested products) and 120 EC-C_{18} (for trypsin-digested products) reverse phase HPLC columns (100 × 4.6 mm, 5.0 µm particle size) at a flow rate of 0.3 mL/min. The mobile phase was composed of H_2O (A) and MeCN (B) both supplemented with 0.1% (v/v) FA. A flow rate of 0.3 mL/min was used with the following gradient: 0–3 min: 5% B, 3–15 min: linear gradient to 100% B, 15–18 min: 100% B, 18–20 min: linear gradient to 5% B, 20–22 min: 5% B. Data were acquired in the positive ionization mode within the range *m/z* 100–3000 Da.

TCEP Reduction of bulbicupramide

To a solution of 0.5 mg of bulbicupramide in 0.5 mL water in a 4 µL vial was added 5 µL of 100 mM TCEP. The mixture was stirred at room temperature for 14 h followed by LC/MS analysis as above.

Acid degradation of TCEP-reduced bulbicupramide

To a series of solutions each containing 100 µL of 1.0 mM TCEP-reduced bulbicupramide in water in an HPLC vial was added 100 mM HCl, 0.1% acetic acid, and 0.1% FA, respectively. These reactions were incubated at both room temperature and at 45 ˚C for 72 h. Samples were monitored by LCMS at different time points.

Mass spectrometry with metal infusion

A 20 mM stock of dithiothreitol (DTT) in LC/MS grade water was freshly prepared and used immediately. To a solution of 0.5 mg of bulbicupramide in 0.5 mL LC/MS grade water, 0.5 mL of 20 mM DTT was added, and the mixture was incubated at 37 °C for 1 h. Bulbicupramide was separated from DTT using solid phase extraction on HLB columns (MN Chromabond; 60 µm, 500 mg) prepared following manufacturer specifications. Briefly, columns were conditioned with LC/MS grade methanol, washed with LC/MS grade water then sample was added, washed with LC/MS grade water, and eluted in 80% methanol in water with 1 µM sulfamethazine internal standard.

For post-LC metal infusion analysis, 4 µL were injected into a Vanquish UHPLC system coupled to a Q-Exactive HF orbitrap mass spectrometer (Thermo Fisher Scientific, Bremen, Germany). A 2.6 µM pore, 100 Å particle, 150 mm length, 2.1 mm diameter Kinetex C_{18} column (Phenomenex) was used for chromatographic separation. For gradient elution, a high-pressure binary gradient system was used. The mobile phase consisted of solvent A H_2O + 0.1 % formic acid (FA) and solvent B acetonitrile (ACN) + 0.1 % FA. The flow rate was set to 0.5 mL/min. After injection, the samples were eluted with the following linear gradients: 0–3 min, 2% B, 3–9 min 2-50% B, 9–10 min 50–99% B, followed by a 3 min washout phase at 99% B and a 2.9 min re-equilibration phase at 2% B. Data dependent acquisition (DDA) of MS/MS spectra was performed in positive mode. Electrospray ionization (ESI) parameters were set to 50 L/min sheath gas flow, 15 L/min auxiliary gas flow, 1 L/min sweep gas flow and 350 °C auxiliary gas temperature; the spray voltage was set to 3.5 kV and the inlet capillary to 320 °C and 55 S-lens RF level was applied. MS scan range was set to 150–1500 *m/z* with a resolution at *m/z* 200 (Rm/z 200) of 120,000 with one micro-scan. The maximum ion injection time was set to 100 ms with an automated gain control (AGC) target of 1.0E6. Up to five MS/MS spectra per MS1 survey scan were recorded DDA mode with Rm/z 200 of 15,000 with one micro-scan. The maximum ion injection time for MS/MS scans was set to 200 ms with an AGC target of 5E5 ions. The MS/MS precursor isolation window was set to *m/z* 1. Normalized collision energy was set to a stepwise increase from 20 to 30 to 40%. MS/MS scans were triggered at the apex of chromatographic peaks within 2 to 15 s from their first occurrence. Dynamic precursor exclusion was set to 5 s. Ions with unassigned charge states were excluded from MS/MS acquisition as well as isotope peaks.

A 10 mM solution of each metal ($FeCl_3$ and $CuSO_4$) was prepared as the stock solution for metal infusion. $FeCl_3$ metal stock was diluted to a final concentration of 1 mM. $CuSO_4$ was diluted to a final concentration of 1 mM in the presence of 2 mM ascorbic acid (for reduction to Cu^+) immediately prior to infusion experiments. Excess ascorbic acid was added and attention to prepare this dilution immediately prior to infusion was taken. No infusion (control) and metal solutions were infused post-LC at a flow rate of 5 µL/min from an external syringe pump, following previously described protocols.[16]

Mass spectrometry with metal infusion competition assays

Bulbicupramide was reduced and analyzed by mass spectrometry as described in the section above (*Mass spectrometry with metal infusion*). Metal competition experiments were performed in the following way: 10 mM solutions of each metal ($CuSO_4$, $NiSO_4$, $ZnSO_4$, $MnSO_4$) were prepared as a stock solution in LC/MS grade water. A combined solution of 500 µM of each metal ($CuSO_4$, $NiSO_4$, $ZnSO_4$, $MnSO_4$) was prepared as the final concentration for the competitive metal infusion. Prior to mixing all metals, $CuSO_4$ was mixed with ascorbic acid in a ratio of 1:4 (for reduction to Cu^+) respectively. All metal solutions prepared were mixed and filtered using a syringe filter (0.45µm PTFEL/L VMR) prior to mass spectrometry analysis. Water (control) and the mixture of the metal solutions were infused post-LC at a flow rate of 5 µL/min from an external syringe pump.

***Microbulbifer* proteomic response to copper exposure**

The strain *Microbulbifer* sp. VAAF005 was cultivated in 250 mL shake flasks each containing 50 mL of half strength marine broth (MB½) liquid media and incubated at 30 °C for 1 d with shaking. From this liquid culture, 0.5 mL was inoculated into fresh 50 mL MB½ liquid media in 250 mL shake flasks containing 0.0, 0.1, and 0.2 mM $CuSO_4$ in triplicate. Cultures were incubated at 30 °C for 3 d with shaking. After 3 d, liquid cultures were transferred into 50 mL tubes and centrifuged (3700×g, 20 min, 4 °C) to harvest the cell pellets.

The cell pellets were resuspended in a buffer containing 50 mM HEPES-Na (pH 7.4), 150 mM NaCl, 0.5% sodium deoxycholate (SDC), and protease inhibitor cocktail (one tablet (complete mini, Roche) per 10 mL of lysis buffer) at 4 °C. Cells were lysed using the MiniBeadbeater (Biospec) at maximum speed, three cycles of 30 s each, with 2 min pauses between cycles to avoid overheating the lysates. After centrifugation, lysates were transferred to new tubes.

The cell lysates were reduced by addition of 10 mM dithiothreitol (DTT) (56 °C, 30 min), followed by alkylation with 20 mM iodoacetamide (room temperature, 30 min, in dark). Proteins were purified by the methanol–chloroform precipitation method. The purified proteins were digested with trypsin (Promega) at a protein:enzyme ratio of ~100:1 in 50 mM HEPES-Na (pH 8.0) buffer at 37 °C overnight. Digestion was quenched by the addition of TFA to a final concentration of 0.1%, and the precipitate was removed by centrifugation at 5,000×g for 10 min. The supernatant was collected, and peptides were purified using a Sep-Pak tC18 cartridge (Waters).

For LC/MS/MS analysis, samples were dissolved in 5% MeCN supplemented with 4% (v/v) formic acid, and 4 µL of the solution were loaded by a Dionex WPS-3000TPLRS autosampler (UltiMate 3000 thermostatted Rapid Separation Pulled Loop Wellplate Sampler) onto a microcapillary column packed with C_{18} beads (ReproSil-Pur 120 C18-AQ, 1.9 µm, Dr. Maisch). Peptides were separated using a nanoflow reversed phase HPLC (Ultimate 3000 RSLCnano, Dionex). The buffer A comprised of 0.1% (v/v) formic acid in water, and the buffer B contained 0.1% (v/v) formic acid in MeCN. The microcapillary column was directly coupled for MS analysis using a Nanospray Flex ion source. The peptides were analyzed using an Orbitrap Exploris 480 mass spectrometer (Thermo). The samples were separated using a 67 min gradient of 2–20% buffer B at 0.3 µL/min. The full MS spectra were recorded using the following parameters: MS scan range: 350–1400 *m/z*; resolution: 60,000; Maximum injection time: Auto; AGC target: Standard. The precursor ions were selected for fragmentation by data-dependent acquisition (DDA). Most abundant precursor ions in each full MS were sequentially selected for fragmentation for 2 s at maximum. The following parameters were used for tandem MS scanning: isolation width: 1.2 *m/z*; resolution: 15,000; normalized collision energy: 36%; AGC target: standard; max injection time: 100 ms. The selected ions were excluded for 45 s. These procedures are well established in our laboratories.[17-19]

Raw files were converted to mzML format using msConvert from Proteowizard, with peak picking and zero samples from the vendor algorithm.[20] Converted files were searched using MSFragger (version 4.1) in Fragpipe (version 22.0) against the *Microbulbifer* sp. VAAF005 annotated open reading frames (ORFs) (4,673 entries).[21] The enzyme name was set to trypsin and up to two missed cleavages were allowed. Peptide length was set to 7–50 and peptide mass range was set to 500–5,000 Da. Oxidation at methionine (+15.9949 Da) was searched as variable modification and cysteine carbamidomethylation (+57.02146 Da) was searched as a fixed modification.

Solid and liquid culture copper toxicity assays

On day 1, *E*. coli co-transformed with MmrA cassettes-1+2+3, MmrB, and MmrC and *E*. coli co-transformed with MmrA cassettes-1+2+3 and empty pCDF-Duet vector were cultured in 10 mL terrific broth (LB) supplemented with kanamycin (50 µg/mL, final concentration) and streptomycin (50 µg/mL, final concentration). The cells were grown at 37 ℃ overnight with shaking. On day 2, 0.5 mL of each culture was inoculated into 10 mL fresh LB supplemented with kanamycin (50 µg/mL, final concentration) and streptomycin (50 µg/mL, final concentration) and grown at 37 °C with shaking until OD_{600} reached 0.6–0.7. Then 5 mL of serially diluted samples (10×, 10^2×, 10^3×, 10^4×) were prepared from these two cultures.

For solid culture assay, a 1 μL inoculum of each diluted sample was spotted on LB agar plate supplemented with kanamycin (50 μg/mL, final concentration), streptomycin (50 μg/mL, final concentration), IPTG (0.3 mM, final concentration), and different $CuSO_4$ concentrations (0 mM, 1 mM, 3 mM, 5 mM, final concentrations). They were incubated at 30 °C for 2 d. Additionally, the 10^3× diluted samples were streaked on LB agar plate supplemented with 1 mM $CuSO_4$.

For liquid culture assay, 0.6 mL of the abovementioned 10^2× diluted samples were inoculated into 50 mL LB media supplemented with kanamycin (50 μg/mL, final concentration), and streptomycin (50 μg/mL, final concentration), IPTG (0.3 mM, final concentration), and $CuSO_4$ (1 mM, final concentration) in triplicates, respectively. They were inoculated at 37 ℃ with shaking. Their OD_{600} were measured every hour.

Supplementary Tables

Table S1: *Microbulbifer* strains used in this study

Strain name	Source	Genome Size	GC Content (%)	References
**Sponge-derived *Microbulbifer* strains with genomes sequenced in this study**				
Microbulbifer sp. ANSA001	*Smenospongia aurea*	5.0 Mb	48.83	This study
Microbulbifer sp. ANSA002	*Smenospongia aurea*	4.8 Mb	49.27	This study
Microbulbifer sp. ANSA003	*Smenospongia aurea*	5.6 Mb	48.83	This study
Microbulbifer sp. ANSA004	*Smenospongia aurea*	4.8 Mb	49.27	This study
Microbulbifer sp. ANSA005	*Smenospongia aurea*	5.2 Mb	48.69	This study
Microbulbifer sp. AVAC002	*Aiolochroia crassa*	5.2 Mb	48.71	This study
Microbulbifer sp. EKSA005	*Smenospongia aurea*	5.3 Mb	48.56	This study
Microbulbifer sp. EKSA006	*Smenospongia aurea*	5.2 Mb	48.62	This study
Microbulbifer sp. EKSA007	*Smenospongia aurea*	5.3 Mb	48.63	This study
Microbulbifer sp. EKSA008	*Smenospongia aurea*	5.2 Mb	48.71	This study
Microbulbifer sp. JMSA002	*Smenospongia aurea*	4.8 Mb	49.33	This study
Microbulbifer sp. JMSA003	*Smenospongia aurea*	5.2 Mb	48.62	This study
Microbulbifer sp. JMSA004	*Smenospongia aurea*	5.2 Mb	48.62	This study
Microbulbifer sp. JMSA006	*Smenospongia aurea*	5.0 Mb	48.73	This study
Microbulbifer sp. JMSA007	*Smenospongia aurea*	5.2 Mb	48.53	This study
Microbulbifer sp. JMSA008	*Smenospongia aurea*	4.8 Mb	49.34	This study
Microbulbifer sp. JTAC008	*Aiolochroia crassa*	5.3 Mb	48.64	This study
Microbulbifer sp. PAAF003	*Aplysina fulva*	5.2 Mb	48.71	This study
Microbulbifer sp. SSSA002	*Smenospongia aurea*	5.1 Mb	51.45	This study
Microbulbifer sp. SSSA003	*Smenospongia aurea*	4.8 Mb	49.34	This study
Microbulbifer sp. SSSA005	*Smenospongia aurea*	5.4 Mb	48.63	This study
Microbulbifer sp. SSSA007	*Smenospongia aurea*	5.0 Mb	48.73	This study
Microbulbifer sp. SSSA008	*Smenospongia aurea*	5.2 Mb	48.81	This study
Microbulbifer sp. TRSA001	*Smenospongia aurea*	5.3 Mb	48.70	This study
Microbulbifer sp. TRSA002	*Smenospongia aurea*	5.5 Mb	48.61	This study
Microbulbifer sp. TRSA005	*Smenospongia aurea*	4.8 Mb	49.25	This study
Microbulbifer sp. TRSA007	*Smenospongia aurea*	5.1 Mb	48.82	This study
Microbulbifer sp. VAAC004	*Aiolochroia crassa*	4.8 Mb	49.34	This study
Microbulbifer sp. VTAC004	*Aiolochroia crassa*	4.9 Mb	48.87	This study
Microbulbifer sp. VVAC002	*Aiolochroia crassa*	5.3 Mb	48.71	This study
Microbulbifer sp. ZKSA002	*Smenospongia aurea*	5.2 Mb	48.72	This study
Microbulbifer sp. ZKSA004	*Smenospongia aurea*	5.2 Mb	48.62	This study
Microbulbifer sp. ZKSA006	*Smenospongia aurea*	4.9 Mb	51.32	This study
Microbulbifer sp. MLAF003	*Aplysina fulva*	4.9 Mb	49.00	This study

Microbulbifer sp. VAAF005	*Aplysina fulva*	5.4 Mb	48.50	This study
Microbulbifer sp. MKSA007	*Smenospongia aurea*	5.8 Mb	49.00	This study
**Coral-derived *Microbulbifer* strains with genomes sequenced in this study**				
Microbulbifer sp. CnH-101-E	*Colpophyllia natans*	5.0 Mb	49.24	This study
Microbulbifer sp. CnH-101-F	*Colpophyllia natans*	5.0 Mb	49.24	This study
Microbulbifer sp. CnH-101-G	*Colpophyllia natans*	4.6 Mb	50.21	This study
Microbulbifer sp. DLAB2-AA	*Diploria labyrinthiformis*	4.9 Mb	49.34	This study
Microbulbifer sp. DLAB2-AF	*Diploria labyrinthiformis*	4.8 Mb	49.36	This study
Microbulbifer sp. PSTR4-B	*Pseudodiploria* (formerly *Diploria*) *strigosa*	5.0 Mb	49.42	This study
***Microbulbifer* type strains with available draft genomes**				
Microbulbifer aestuariivivens NBRC 112533^T	Tidal flat sediment	3.4 Mb	59.5	GenBank[22]
Microbulbifer aggregans CBB-MM1^T	Mangrove sediment	3.9 Mb	59.00	GenBank[23]
Microbulbifer bruguierae H12^T	Mangrove plant	4.5 Mb	56.50	GenBank[24]
Microbulbifer celer KCTC 12973^T	Marine solar saltern	4.3 Mb	57.00	GenBank[25]
Microbulbifer donghaiensis CGMCC 1.7063^T	Marine sediment	4.3 Mb	59.50	GenBank[26]
Microbulbifer echini JCM 30400^T	Sea urchin	4.4 Mb	51.00	GenBank[27]
Microbulbifer elongatus DSM 6810^T	Sea water	4.2 Mb	57.50	GenBank[28]
Microbulbifer epialgicus DSM 18651^T	Marine algae	5.7 Mb	49.00	GenBank[29]
Microbulbifer flavimaris WRN-8^T	Marine sediment	3.6 Mb	60.00	GenBank[30]
Microbulbifer guangxiensis L3^T	Tidal flat sediment	3.7 Mb	60.00	GenBank[31]
Microbulbifer halophilus KCTC 12848^T	Saline soil	4.7 Mb	61.50	GenBank[32]
Microbulbifer harenosus HB161719^T	Coastal sand	4.7 Mb	58.00	GenBank[33]
Microbulbifer hydrolyticus IRE-31^T	Black liquor	4.2 Mb	57.50	GenBank[34]
Microbulbifer magnicolonia GG15^T	Tidal flat sediment	4.3 Mb	61.50	GenBank[35]
Microbulbifer mangrovi DD-13^T	Mangrove water	4.5 Mb	57.00	GenBank[36]
Microbulbifer marinus CGMCC 1.10657^T	Marine sediment	4.0 Mb	60.00	GenBank[37]
Microbulbifer pacificus SPO729^T	Marine sponge	4.2 Mb	58.50	GenBank[38]
Microbulbifer rhizosphaerae CECT 8799 ^{T}	Plant	5.3 Mb	60.00	GenBank[39]
Microbulbifer sediminum TT37^T	Tidal flat sediment	3.9 Mb	61.00	GenBank[31]
Microbulbifer spongiae MI-G^T	Marine sponge	4.5 Mb	53.50	GenBank[40]
Microbulbifer taiwanensis LMG 26125^T	Coastal soil	4.8 Mb	60.00	GenBank[41]
Microbulbifer thermotolerans DSM 19189^T	Deep-sea sediment	3.9 Mb	56.90	GenBank[42]
Microbulbifer variabilis ATCC 700307^T	Marine algae	4.8 Mb	49.00	GenBank[29]
Microbulbifer yueqingensis CGMCC 1.10658^T	Marine sediment	3.7 Mb	62.00	GenBank[37]
Microbulbifer zhoushanensis TT30^T	Tidal flat sediment	4.1 Mb	61.50	GenBank[31]
***Microbulbifer* type strains with only 16S rRNA gene sequences available**				
Microbulbifer agarlyticus JAMB A3^T	Deep-sea sediment	n/a	n/a	GenBank[42]
Microbulbifer chitinlyticus JCM 15148^T	Mangrove forests	n/a	n/a	GenBank[43]
Microbulbifer gwangyangensis GY2^T	Tidal flat	n/a	n/a	GenBank[38]
Microbulbifer maritimus TF-17^T	Intertidal sediment	n/a	n/a	GenBank[44]

Microbulbifer okhotskensis KMM 9862^T	Okhotsk Sea sediment	n/a	n/a	GenBank[45]
Microbulbifer okinawensis ABABA23^T	Mangrove forests	n/a	n/a	GenBank[43]
**Outgroup strains with available draft genomes**				
Aeromonas hydrophila ATCC 7966^T		4.7 Mb	61.50	GenBank[46]
Escherichia coli ATCC 11775^T		5.0 Mb	50.50	GenBank[47]
Vibrio cholerae ATCC 14035^T		4.0 Mb	47.50	GenBank[48]

Table S2: Housekeeping genes used to construct the MLSA tree

Gene	Function
dnaB	replicative DNA helicase
rpoA	alpha subunit of the RNA polymerase
pheS	alpha subunit of PheRS
atpA	alpha subunit of ATP synthase
aspS	aspartyl-tRNA ligase
groEL	chaperone GroEL
gltA	citrate synthase
odhB	2-oxoglutarate dehydrogenase complex
sucC	beta subunit of succinyl-CoA synthetase
sucD	alpha subunit of succinyl-CoA synthetase
tpiA	triosephosphate isomerase
ffh	homolog of the eukaryotic signal recognition particle
asnS	asparagine synthase
atpD	beta subunit of ATP synthase
ldpA	involved in circadian clock/electron transport

Table S3: Amino acid sequences of the MmrA substrate peptides used in this study

Gene names	Amino acid sequences
MmrA cassette-1+2+3	MNVSAANSNANPFAAADLSSGYNVALADHDGDDKKKEGKCGEGKCGEGKKKEGKCGEGKCGEGKKKEGKCGEGKCGG
MmrA cassette-1+2	MNVSAANSNANPFAAADLSSGYNVALADHDGDDKKKEGKCGEGKCGEGKKKEGKCGEGKCGEG
MmrA cassette-1	MNVSAANSNANPFAAADLSSGYNVALADHDGDDKKKEGKCGEGKCGEG
MmrA cassette-1 C40A	MNVSAANSNANPFAAADLSSGYNVALADHDGDDKKKEGKAGEGKCGEG
MmrA cassette-1 C45A	MNVSAANSNANPFAAADLSSGYNVALADHDGDDKKKEGKCGEGKAGEG
MmrA cassette-1 C40A/C45A	MNVSAANSNANPFAAADLSSGYNVALADHDGDDKKKEGKAGEGKAGEG

Table S4: Theoretical and experimentally observed peptide ions described this study

Molecules	Theoretical mass	Observed mass	Mass error (ppm)
cassette-1 unmodified	532.8472	not observed	not applicable
cassette-1 modified	531.2346	531.2356	1.8
cassette-1+2 unmodified	836.3961	not observed	not applicable
cassette-1+2 modified	833.1709	833.1741	3.8
cassette-1+2+3 unmodified	1114.5300	not observed	not applicable
cassette-1+2+3 modified	1109.6923	1109.7009	7.7
cassette-1 C40A unmodified	526.4528	526.4556	5.3
cassette-1 C40A modified	525.6466	525.6505	7.4
cassette-1 C45A unmodified	526.4528	526.4567	7.4
cassette-1 C45A modified	525.6466	525.6490	4.5
cassette-1 C40A/C45A unmodified	520.0584	520.0618	6.5
cassette-1 C40A/C45A modified	520.0584	520.0618	6.5
reduced bulbicupramide	573.2030	573.2029	0.2
bulbicupramide–Cu^{1+} complex	604.1639	604.1648	1.5
bulbicupramide–Fe^{3+} complex	599.6584	599.6589	0.8

Table S5: ^{1}H (700 MHz) and ^{13}C (176 MHz) NMR chemical shifts of oxidized bulbicupramide in DMSO-d_6 (J in Hz, δ in ppm)

Oxidized bulbicupramide						
Residue	No.	δ_H (J, Hz)[a]	δ_C, type[b]	HMBC	COSY	ROESY
Glu^{37}	1		168.6, C			
	2	3.87, overlap	52.5, CH	1, 3, 4	3	Gly^{38}-NH
	3	1.98, m<br>1.94, overlap	26.4, CH_2	1, 2, 4, 5	2, 4	
	4	2.39, m	29.1, CH_2	2, 3, 5	3	
	5		173.4, C			
	NH_2	nd				
Gly^{38}	1		168.6, C			
	2	3.97, dd, (16.7, 5.9)<br>3.83, dd, (16.7, 5.4)	41.8, CH_2	1, Glu^{37}-1	NH	Glu^{37}-2
	NH	8.92, t, (5.5)		1, 2, Glu^{37}-1	2	
Lys^{39}	1		165.4, C			
	2	5.09, dt (8.1, 5.2)	46.8, CH	1, 3, 4, Gly^{38}-1	3, NH	
	3	1.86, m<br>1.80, m	30.5, CH_2		2, 4	
	4	1.30, m<br>1.26, m	20.8, CH_2		3, 5	
	5	1.57, overlap	26.3, CH_2	4, 6	4, 6	
	6	2.77, overlap	38.5, CH_2	4, 5	5	
	NH	7.67, d (7.3)		Gly^{38}-1	2	
	NH_2	nd				
Cys^{40}	1		159.2, C			
	2		nd			
	3		nd			
Gly^{41}	1		169.7, C			
	2	4.04, overlap<br>3.74, overlap	41.4, CH_2	Cys^{40}-1	NH	Glu^{42}-NH
	NH	8.51, overlap		Cys^{40}-1	2	
Glu^{42}	1		172.2, C			
	2	4.06, m	53.2, CH	1, 3	3, NH	Gly^{43}-NH
	3	1.91, overlap<br>1.85, overlap	25.4, CH_2	2, 4	2, 4	
	4	2.22, m	30.0, CH_2	2, 3, 5	3	
	5		173.7, C			
	NH	8.74, d, (5.7)		2, 3, Cys^{41}-1	2	Gly^{41}-2
Gly^{43}	1		168.5, C			
	2	3.87, overlap<br>3.56, dd, (17.5, 5.3)	41.8, CH_2	1, Glu^{42}-1	NH	Lys^{44}-NH
	NH	8.78, br s			2	Glu^{42}-2
Lys^{44}	1		166.4, C			
	2	5.00, dt (7.5, 7.4)	47.2, CH	1, 3, 4	3, NH	
	3	1.93, overlap	31.4, CH_2	2, 4	2, 4	
	4	1.47, m<br>1.39, m	22.1, CH_2	2, 3, 5, 6	3, 5	
	5	1.62, m	26.4, CH_2	3, 4, 6	4, 6	
	6	2.77, overlap	38.4, CH_2	4, 5	5	
	NH	8.89, d (7.6)		2, 3, Gly^{43}-1	2	Gly^{43}-2
	NH_2	nd				
Cys^{45}	1		159.0, C			
	2		nd			
	3		nd			
Gly^{46}	1		168.5, C			
	2	3.92, overlap<br>3.88,overlap	42.0, CH_2	Cys^{45}-1	NH	Glu^{47}-NH

	NH	8.50, overlap		Cys[45]-1	2	
Glu[47]	1		171.5, C			
	2	4.31, dt (8.0, 5.6)	51.9, CH	3, 4	3, NH	Gly[48]-NH
	3	1.93, overlap<br>1.79, overlap	27.5, CH_2	2	2, 4	
	4	2.28, m	30.1, CH_2	2, 3, 5	3, 5	
	5		174.0, CH_2			
	NH	8.31, d (8.0)		2, 3, Gly[46]-1	2	Gly[46]-2
Gly[48]	1		171.0, C			
	2	3.76, d (6.0)<br>3.72, d (5.7)	40.7, CH_2	1	NH	
	NH	8.41, t (6.0)		2, Glu[47]-1	2	Glu[47]-2

[a]Recorded at 700 MHz. [b]Recorded at 176 MHz. [nd]Not detected.

Table S6: Summary of peptidic molecules with thiooxazole or oxazolone-thioamide motifs.

Molecules	Core sequences	Structures	λ_{max} UV absorbance	Susceptible to acid degradation	Ref.
Bulbicupramide	KXG<br><br>KXG	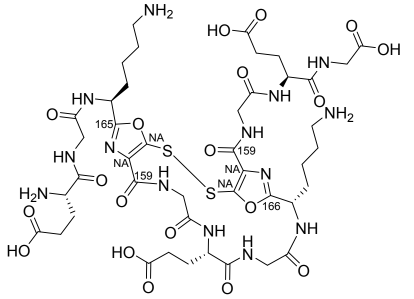<br>Thiooxazole	302 nm	No	This study
Bufferin	DXK<br><br>SXA	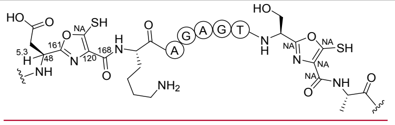<br>Thiooxazole	305 nm	No	49
SbtMa peptide	GXG	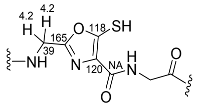<br>Thiooxazole	304 nm	Not tested	50
Oxazolin	KXG<br><br>KXG	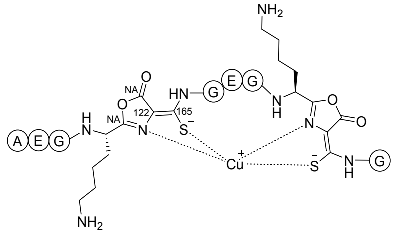<br>Oxazolone-thioamide	302 nm	No	51
Mov XBC	TXG	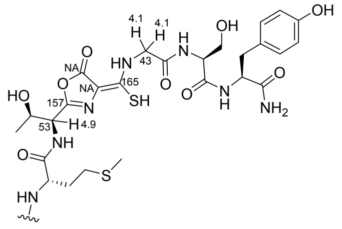<br>Oxazolone-thioamide	302 nm	Yes	52
Methanobactin from *Methylosinus*. sp. LW4	MXA<br><br>WXG	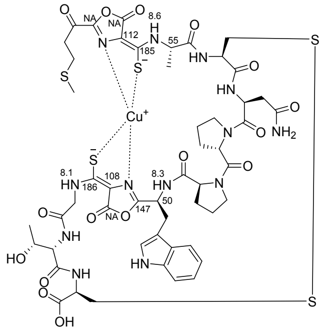<br>Oxazolone-thioamide	342, 394 nm	Yes	53

Methanobactin from *Methylosinus trichosporium* OB3b	LXG PXS	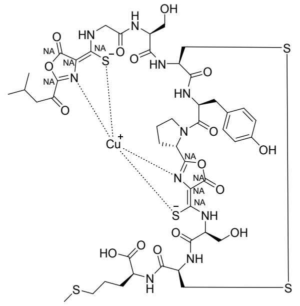 Oxazolone-thioamide	340, 394 nm	Yes	54, 55

X: modified amino acid residues

NA: Not assigned.

SUPPLEMENTARY FIGURES

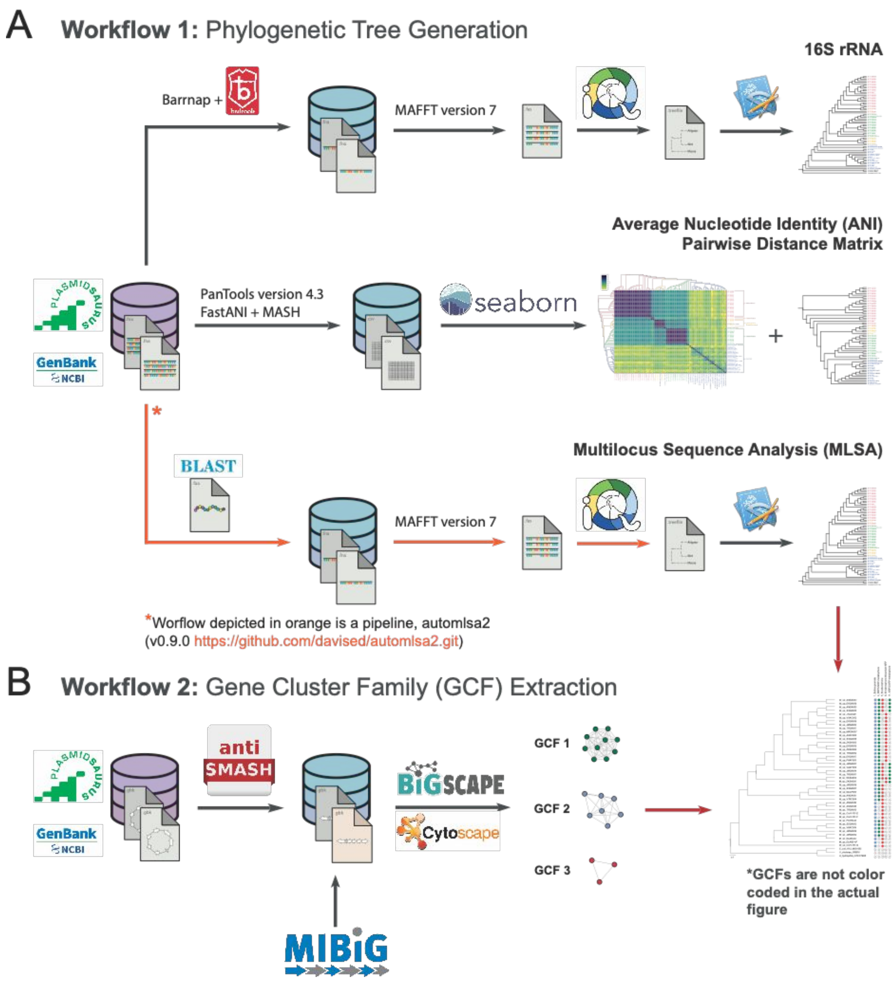

Figure S1: Cartoon depicting the phylogenomic workflows. (**A**) Barrnap 0.9,[1] Mafft version 7,[2] and IQ-TREE2[3] were employed to extract 16S rRNA sequences and generate a maximum likelihood tree. PanTools,[11] FastANI,[12] and MASH[13] were employed to extract orthologous genes and generate a neighborhood joining tree and heatmap representation of pairwise distances. An automated MLSA pipeline, automlsa2,[10] was employed to extract, align, and concatenate 15 housekeeping genes and generate a maximum likelihood tree. (**B**) Workflow diagram depicting the annotation, networking, and extraction of GCFs using antiSMASH,[56] MIBiG,[57] Big-SCAPE 1.0.[15] The MLSA tree and GCFs were connected via the GCF presence/absence matrix. Construction of the GCF presence/absence matrix is described in the Supplementary Materials and Methods.

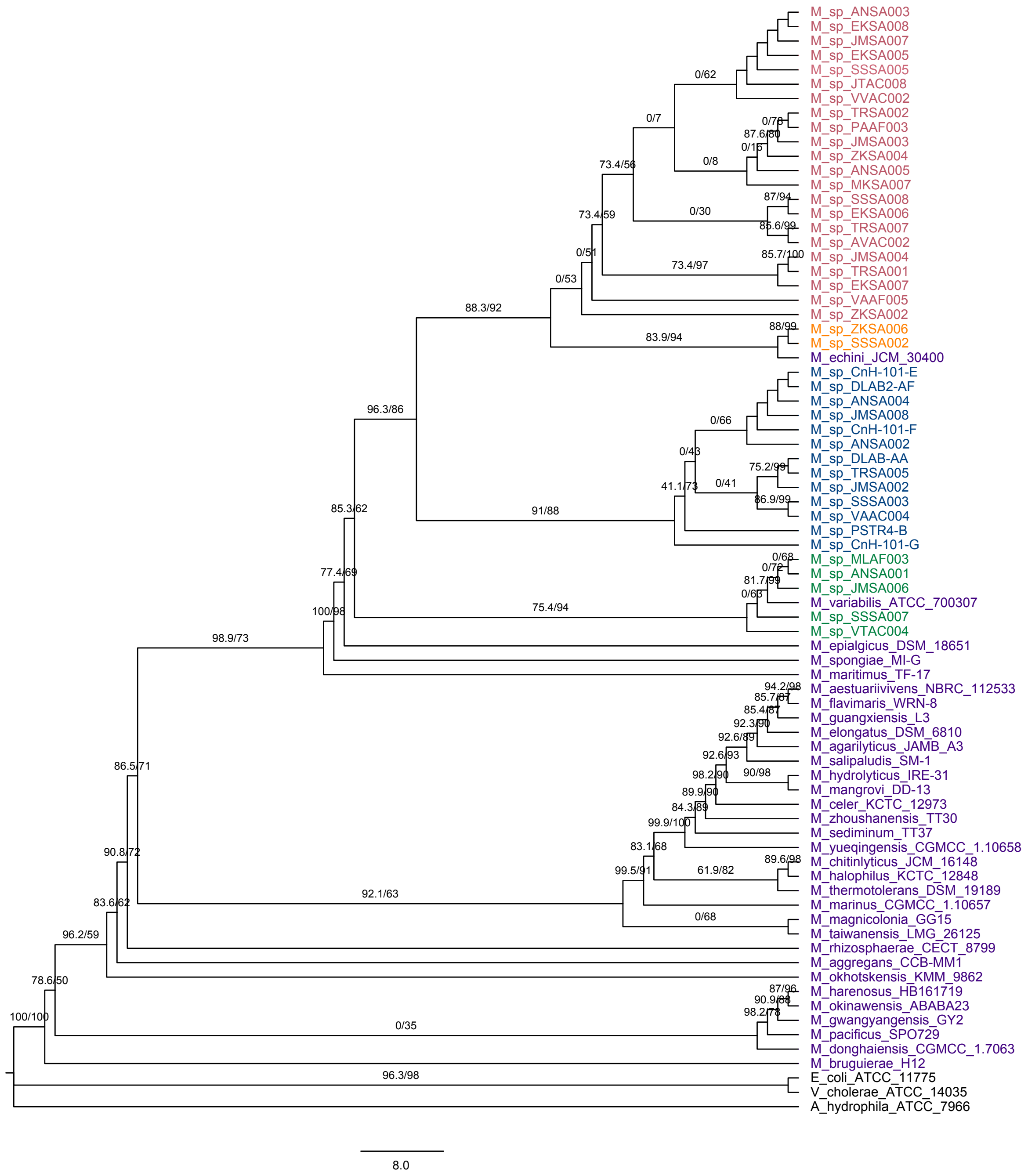

Figure S2: Phylogenetic tree reconstructed by the maximum-likelihood method using 16S rRNA gene sequences. Subclades are highlighted to effectively compare phylogenetic estimations across three methods of phylogenetic tree construction. Three strains—*Escherichia coli* ATCC 11775, *Vibrio cholerae* ATCC 14035, and *Aeromonas hydrophilia* ATCC 7966—are selected as outgroups to root the tree. Shimodaira-Hasegawa approximate Likelihood Ratio Test (SH-aLRT)[7]/ultrafast bootstrap values[5,6] are both displayed.

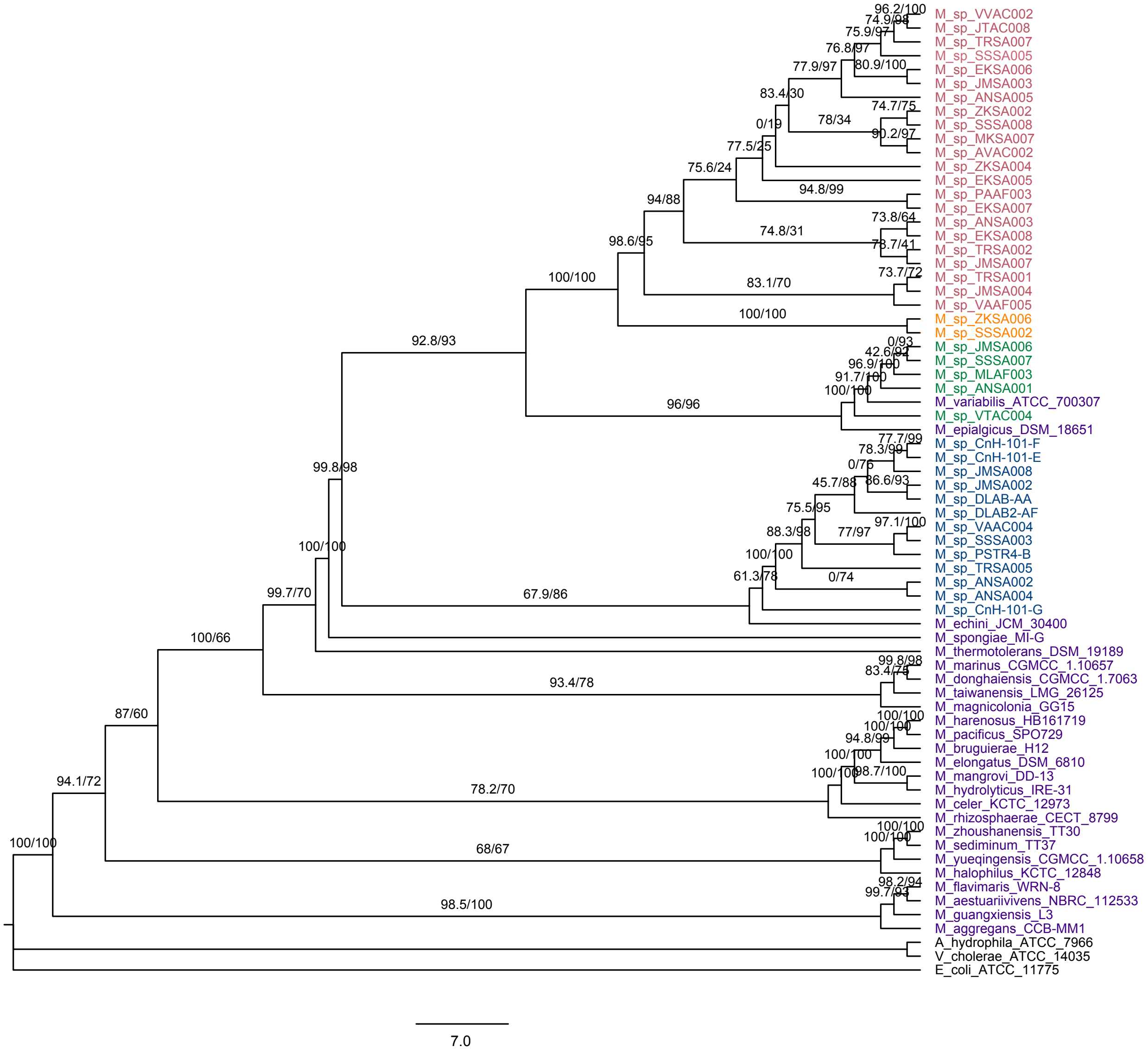

Figure S3: Phylogenetic tree reconstructed by the maximum-likelihood method based on concatenating 15 gene sequences (Table S2). The 25 *Microbulbifer* type strains are included in this analysis. Subclades are highlighted to effectively compare phylogenetic estimations across three methods of phylogenetic tree construction. Three strains—*E. coli* ATCC 11775*, V. cholerae* ATCC 14035*,* and *A. hydrophilia* ATCC 7966—are selected as outgroups to root the tree. Shimodaira-Hasegawa approximate Likelihood Ratio Test (SH-aLRT)[7]/ultrafast bootstrap values[5,6] are both displayed.

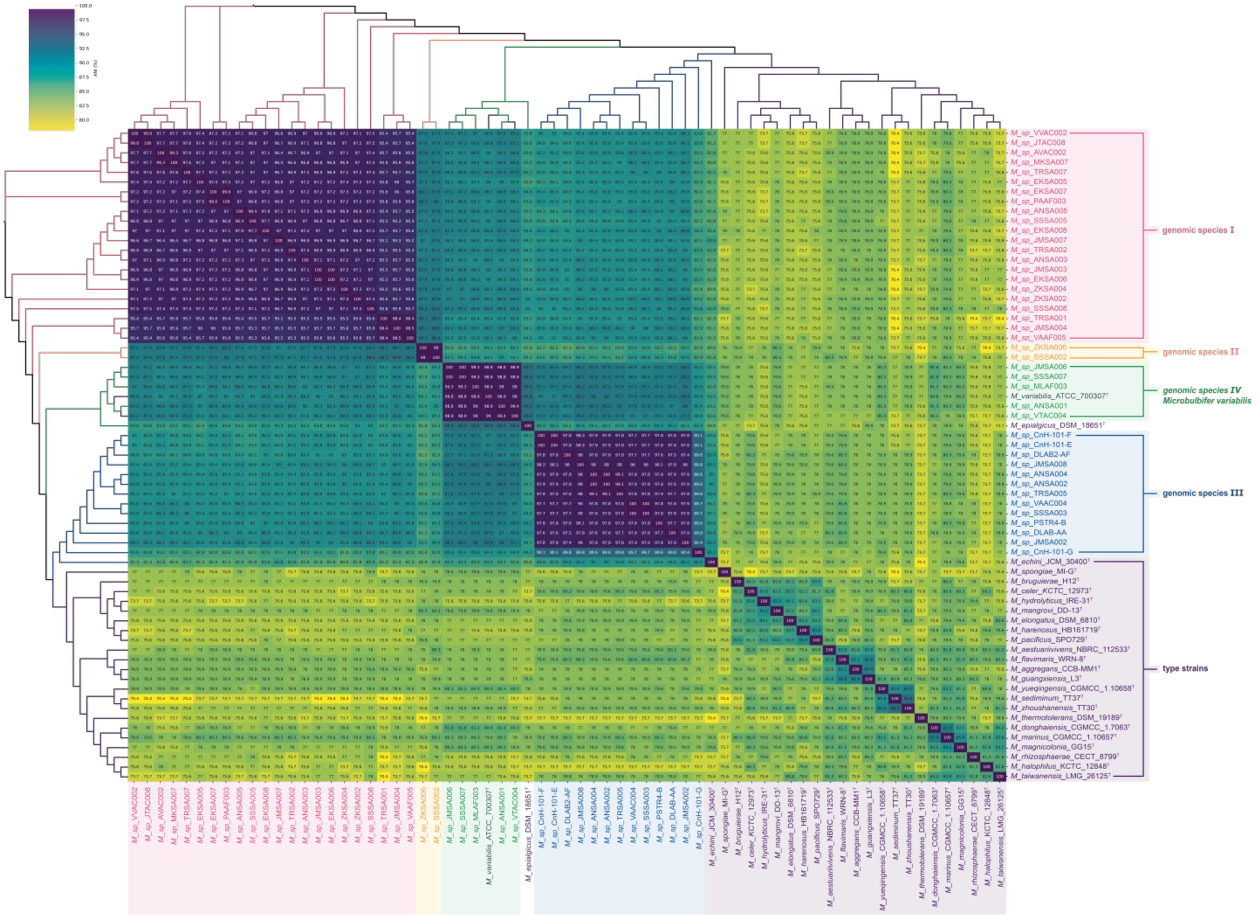

Figure S4: Heatmap representation of pairwise distances in a hierarchal clustering analysis derived from ANI based calculations. Clades with ANI similarity scores ≥95% formed genomic species (GS- I–IV). Note an exception with this grouping can be found within GS-III. *Microbulbifer* sp. CnH-101-G has ANI scores that are between 89% and 95% relative to other strains found in GS-III. Although an ANI similarly score of ≤95% denotes that strain CnH-101-G is likely not the same species as the rest of GS-III, GS-III is the closest species group to this strain.

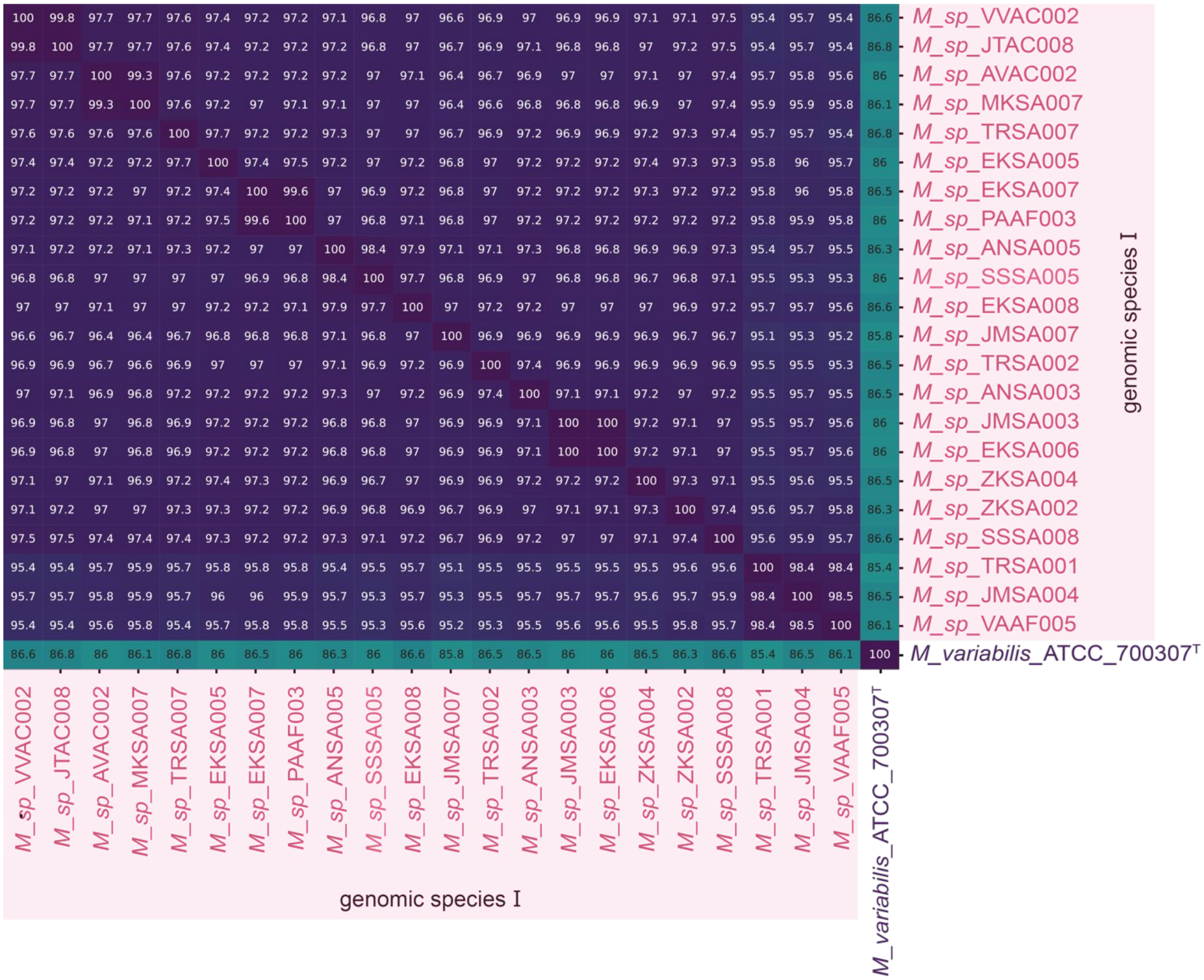

Figure S5: The ANI matrix for GS-I expanded to highlight the pairwise ANI scores. A cutoff of 95% in ANI similarity score is a well-accepted metric species assignement.[58] The 22 GS-I strains demonstrated ANI similarity scores of less than 86.8% to the closest type strain *Microbulbifer variabilis,*[59] thus supporting the classification of this clade as part of a novel genomic species.

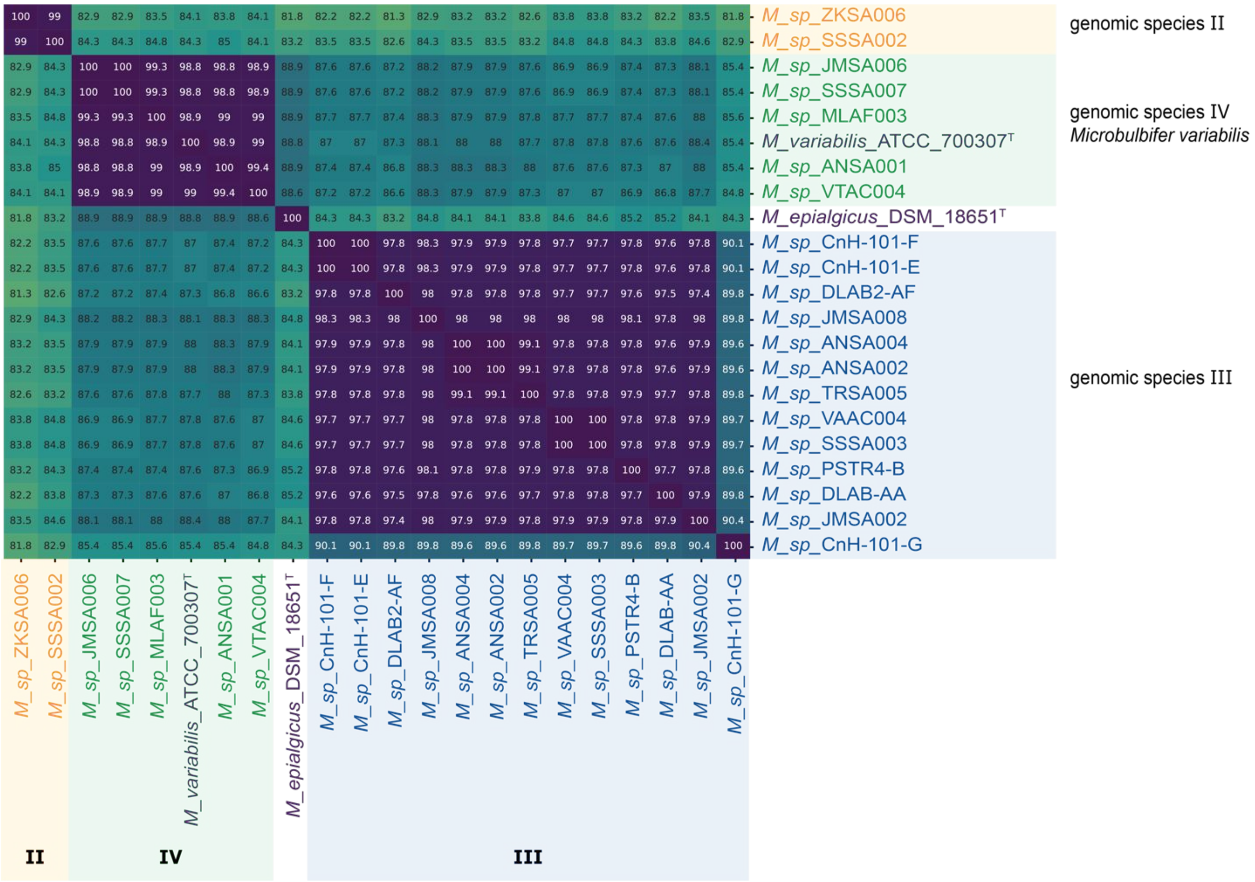

Figure S6: The ANI matrix for GS-II–IV expanded to highlight the pairwise comparison scores. The two GS-II strains displayed ANI values less than 84.8% similarity to the closest recognized species, *M. variabilis,*[59] supporting the classification of this clade as part of a novel genomic species. The five strains in GS-IV display ANI values greater than 98.8% similarity to *M. variabilis,*[59] classifying these five strains as *M. variabilis.*[59] One strain, CnH-101-G, was an outlier. The ANI value of strain CnH-101-G to the closest clade—strains in GS-III—is between 89.4%–90.4%. Therefore, strain CnH-101-G was not classified as a novel genomic species. Instead, it was classified as part of GS-III. The remaining 12 strains in GS-III displayed ANI values less than 88.4% to the closest recognized species, *M. variabilis,*[59] classifying this clade as part of a novel genomic species.

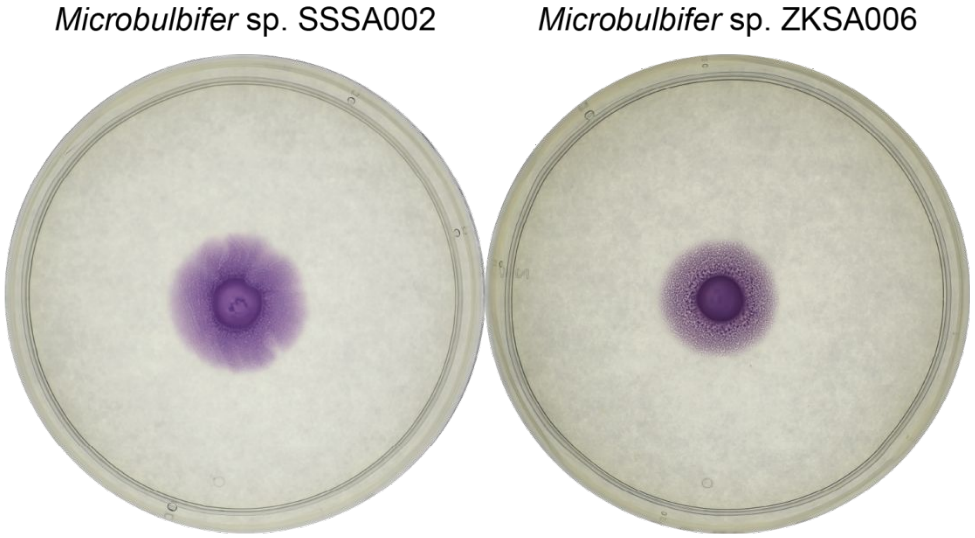

Figure S7: Colony morphology of strains *Microbulbifer* sp. SSSA002 and *Microbulbifer* sp. ZKSA006. Note that the purple coloration is indicative of violacein production.

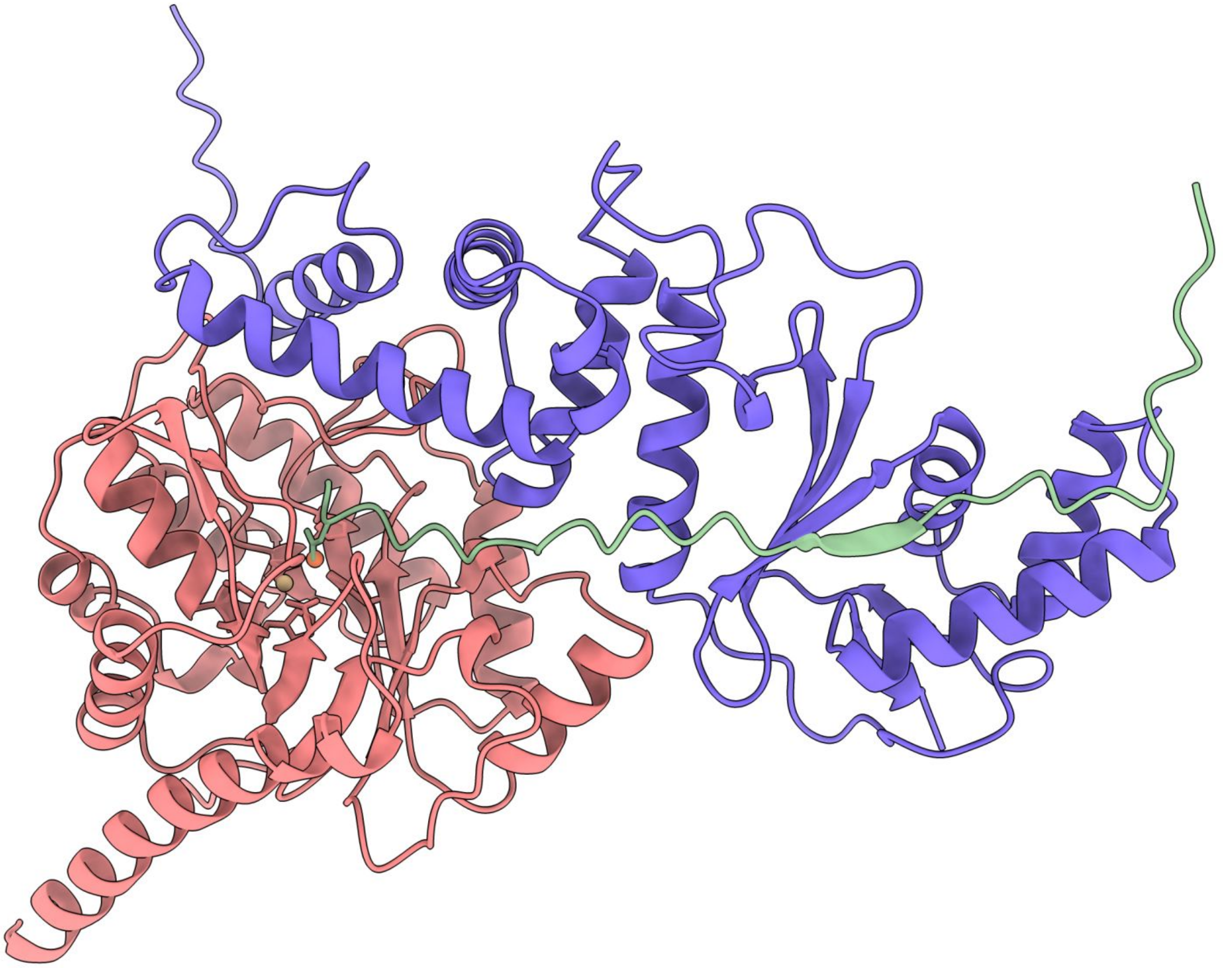

Figure S8: AlphaFold3-predicted model of MmrB (in brown) with MmrC (in blue) together with the MmrA leader peptide (in green). Note that the MmrA leader peptide adopts an isolated β-strand to extend the antiparallel β-sheet of the MmrC RRE domain. Two Fe(III) atoms were included in the AlphaFold3-generated model and they were positioned in the predicted MNIO active site.

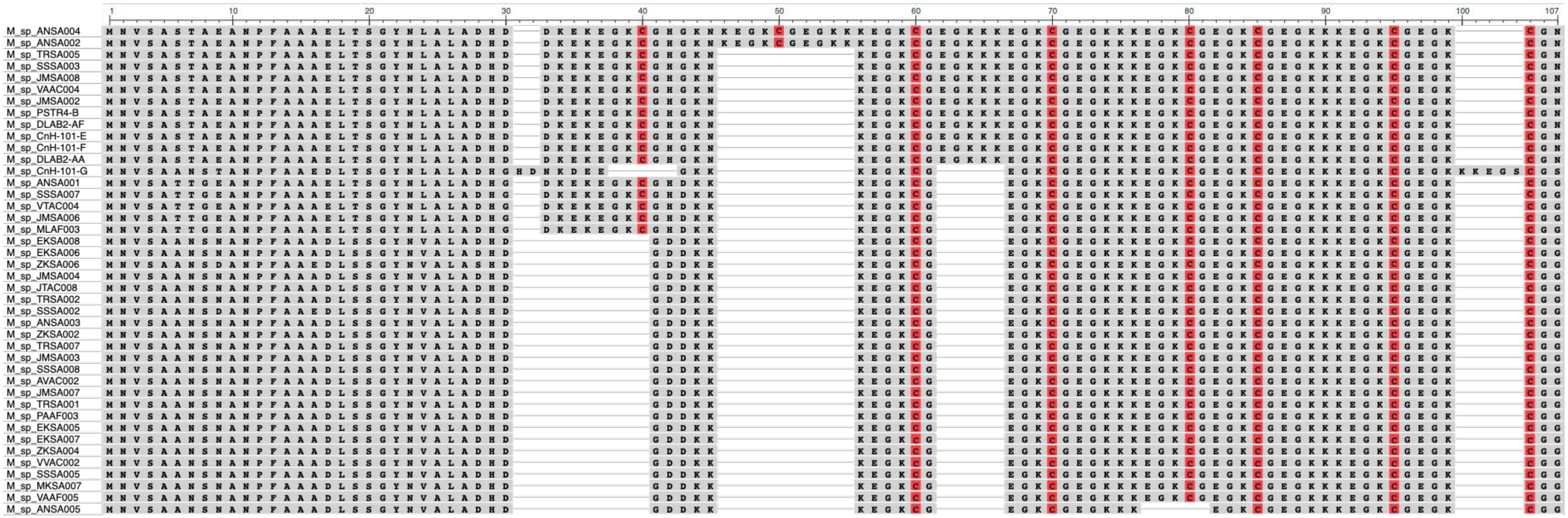

Figure S9: Sequence alignment of MmrA precursor peptides identified in this study. The Cys residues are highlighted in red. Note that the N-terminal leader regions are highly conserved, but the organization of the repeating units in the C-terminal core region is variable. The number of Cys residues (highlighted in red) in the core regions varies from five to eight.

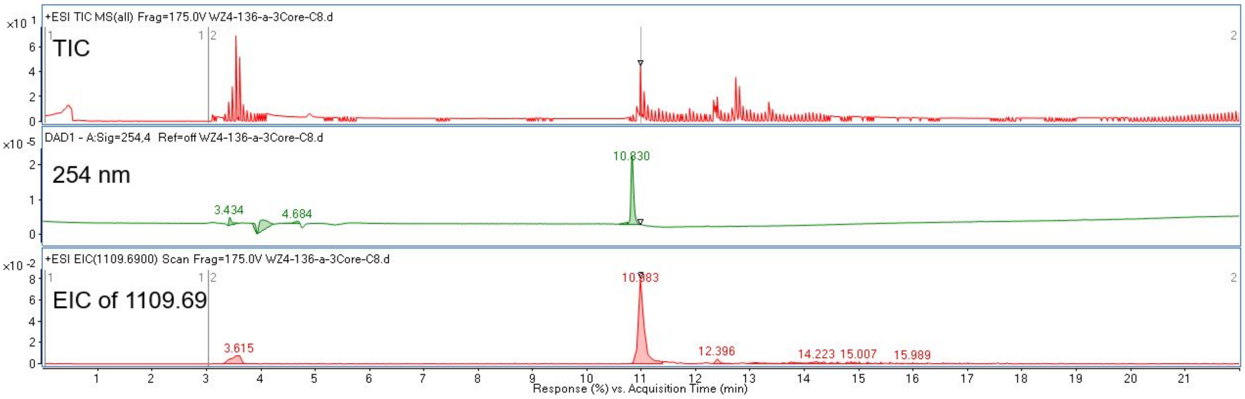

Figure S10: LC/MS analysis of the chymotrypsin-digested modified MmrA peptide bearing cassettes-1+2+3 obtained after coexpression with *mmrB* and *mmrC* genes in *E. coli*. (top) Total ion chromatogram (TIC). (middle) UV-absorbance chromatogram recorded at 254 nm wavelength. (bottom) Extracted ion chromatogram (EIC) for the $[M+5H]^{5+}$ ion corresponding to the modified peptide.

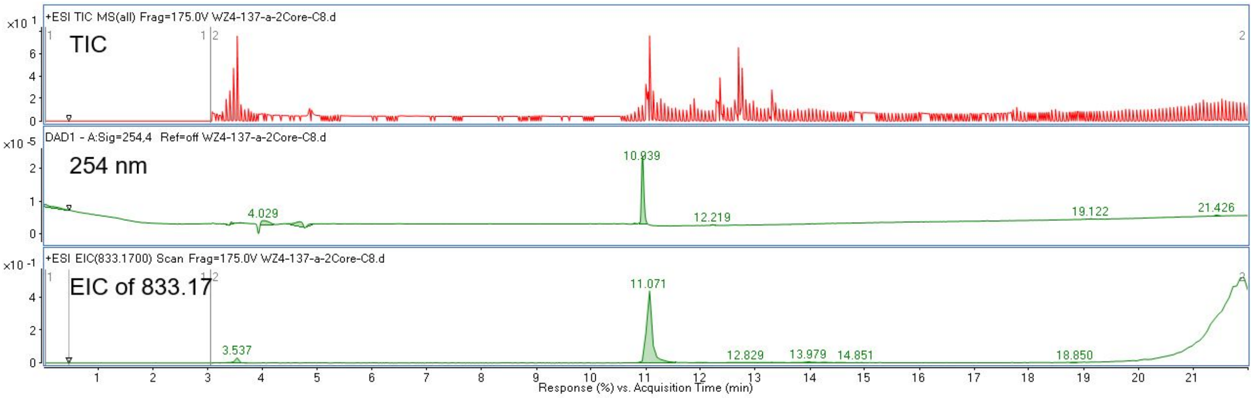

Figure S11: LC/MS analysis of the chymotrypsin-digested modified MmrA peptide bearing cassettes-1+2 obtained after coexpression with *mmrB* and *mmrC* genes in *E. coli*. (top) TIC. (middle) UV-absorbance chromatogram recorded at 254 nm wavelength. (bottom) EIC for the $[M+5H]^{5+}$ ion corresponding to the modified peptide.

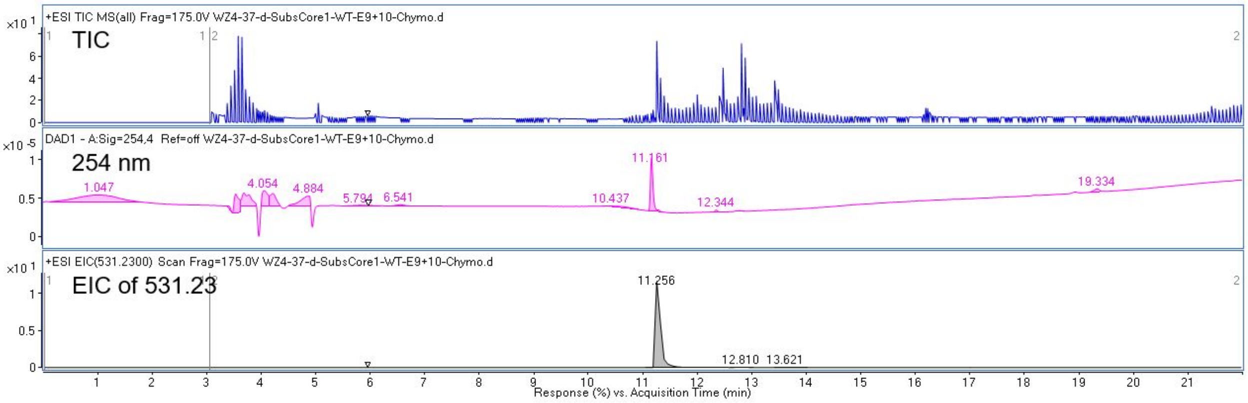

Figure S12: LC/MS analysis of the chymotrypsin-digested modified MmrA peptide bearing cassette-1 only obtained after coexpression with *mmrB* and *mmrC* genes in *E. coli*. (top) TIC. (middle) UV-absorbance chromatogram recorded at 254 nm wavelength. (bottom) EIC for the $[M+5H]^{5+}$ ion corresponding to the modified peptide.

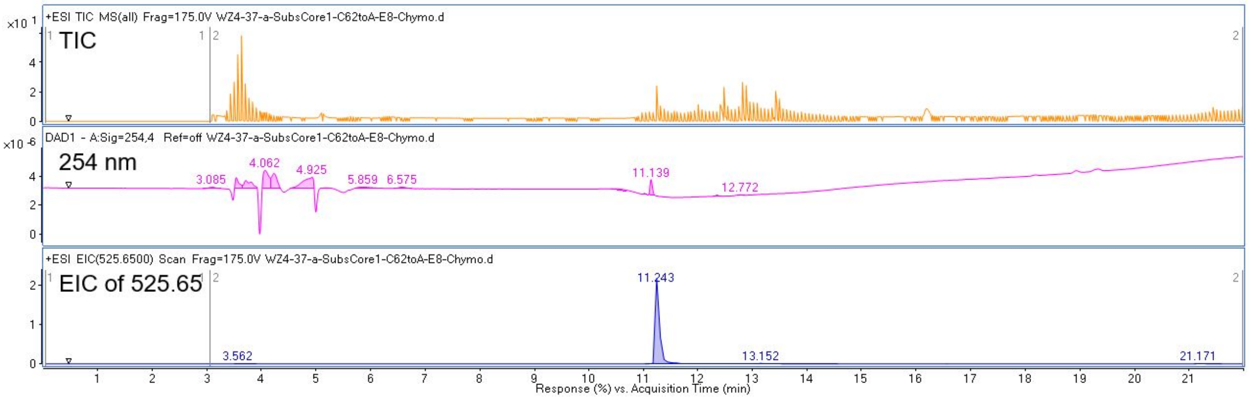

Figure S13: LC/MS analysis of the chymotrypsin-digested modified MmrA peptide bearing cassette-1 C40A mutant obtained after coexpression with *mmrB* and *mmrC* genes in *E. coli*. (top) TIC. (middle) UV-absorbance chromatogram recorded at 254 nm wavelength. (bottom) EIC for the $[M+5H]^{5+}$ ion corresponding to the modified peptide.

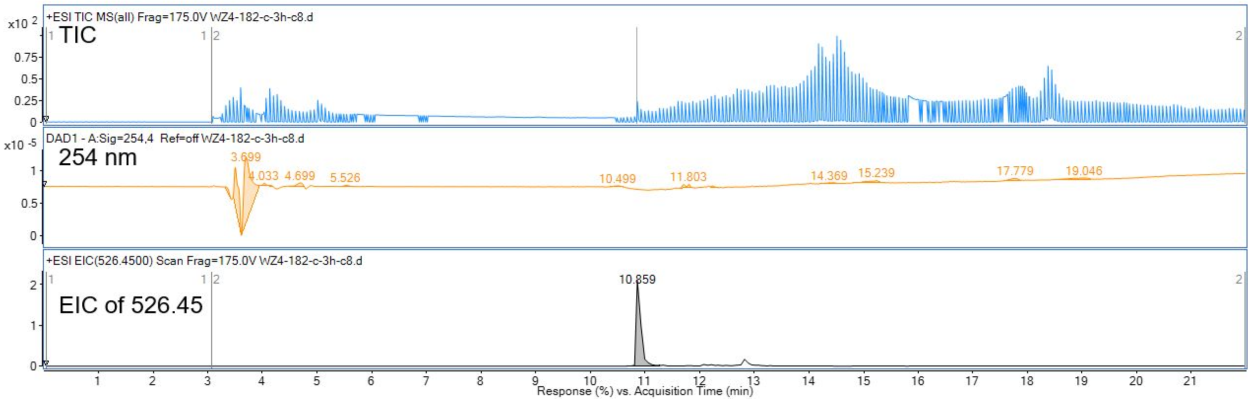

Figure S14: LC/MS analysis of the chymotrypsin-digested unmodified MmrA peptide bearing cassette-1 C40A mutant. (top) TIC. (middle) UV-absorbance chromatogram recorded at 254 nm wavelength. (bottom) EIC for the $[M+5H]^{5+}$ ion corresponding to the unmodified peptide. Note that in the absence of *mmrB* and *mmrC* genes, the abundance of the unmodified MmrA peptide recovered from *E. coli* cultures was vastly reduced.

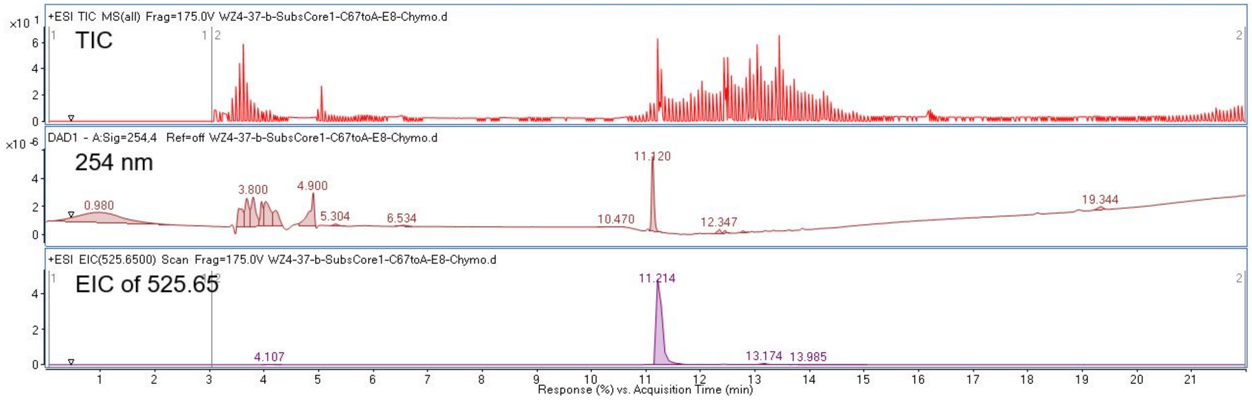

Figure S15: LC/MS analysis of the chymotrypsin-digested modified MmrA peptide bearing <u>cassette-1 C45A mutant</u> obtained after coexpression with *mmrB* and *mmrC* genes in *E. coli*. (top) TIC. (middle) UV-absorbance chromatogram recorded at 254 nm wavelength. (bottom) EIC for the $[M+5H]^{5+}$ ion corresponding to the modified peptide. The MS^1 spectrum of the peptide is illustrated in Figure 2C.

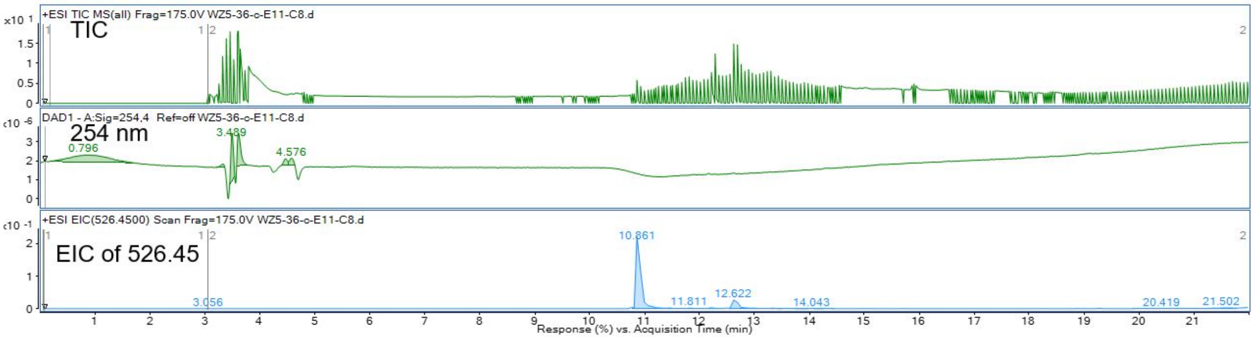

Figure S16: LC/MS analysis of the chymotrypsin-digested unmodified MmrA peptide bearing cassette-1 C45A mutant. (top) TIC. (middle) UV-absorbance chromatogram recorded at 254 nm wavelength. (bottom) EIC for the $[M+5H]^{5+}$ ion corresponding to the unmodified peptide. The MS^1 spectrum of the peptide is illustrated in Figure 2C. As before, in the absence of *mmrB* and *mmrC* genes, the abundance of the unmodified MmrA peptide recovered from *E. coli* cultures was vastly reduced.

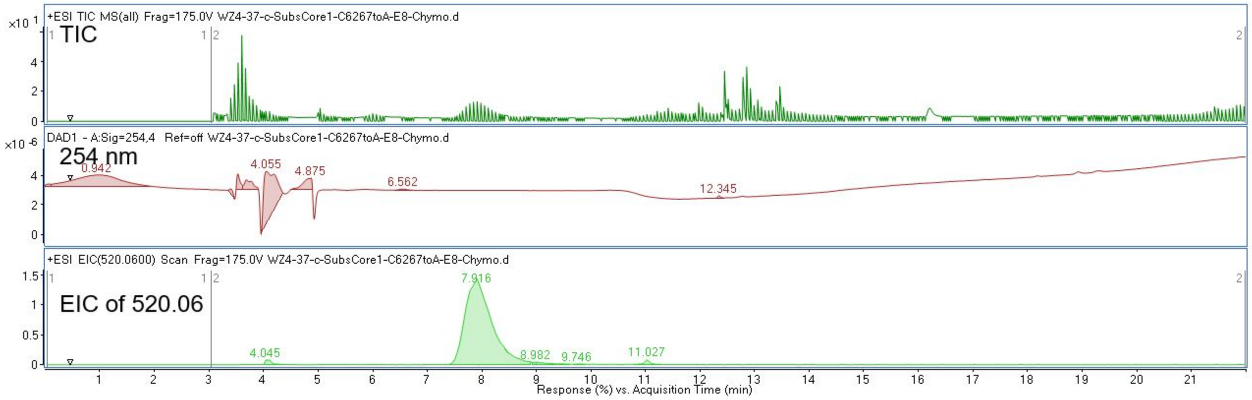

Figure S17: LC/MS analysis of the chymotrypsin-digested modified MmrA peptide bearing cassette-1 C40A/C45A mutant obtained after coexpression with *mmrB* and *mmrC* genes in *E. coli*. (top) TIC. (middle) UV-absorbance chromatogram recorded at 254 nm wavelength. (bottom) EIC for the $[M+5H]^{5+}$ ion corresponding to the modified peptide.

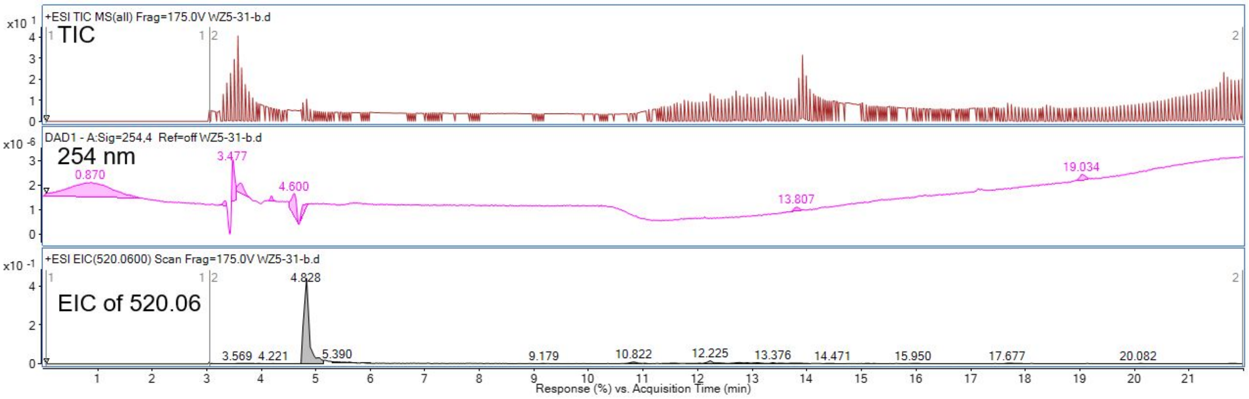

Figure S18: LC/MS analysis of the chymotrypsin-digested unmodified MmrA peptide bearing cassette-1 C40A/C45A mutant. (top) TIC. (middle) UV-absorbance chromatogram recorded at 254 nm wavelength. (bottom) EIC for the $[M+5H]^{5+}$ ion corresponding to the unmodified peptide. As before, in the absence of *mmrB* and *mmrC* genes, the abundance of the unmodified MmrA peptide recovered from *E. coli* cultures was vastly reduced.

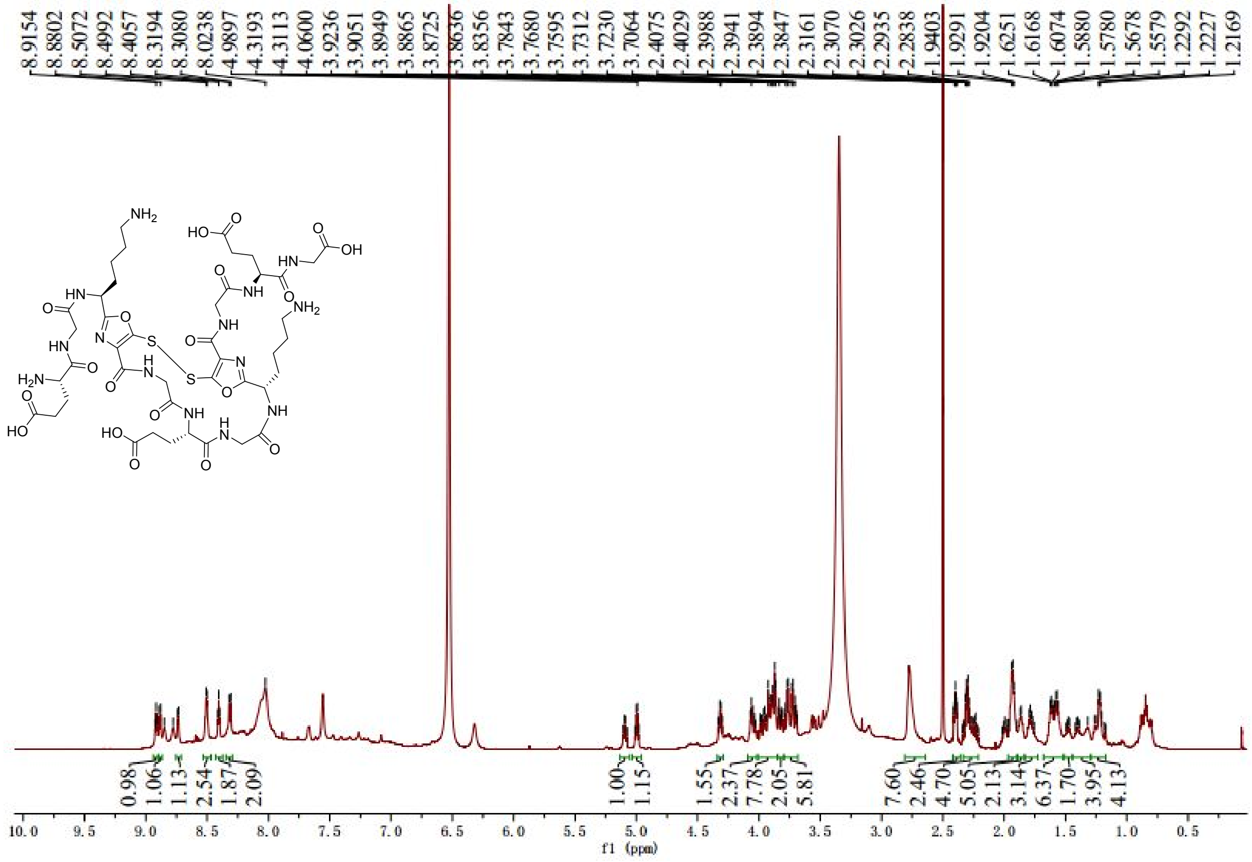

Figure S19: The ^{1}H NMR spectrum of oxidized bulbicupramide (700 MHz, DMSO-d_6).

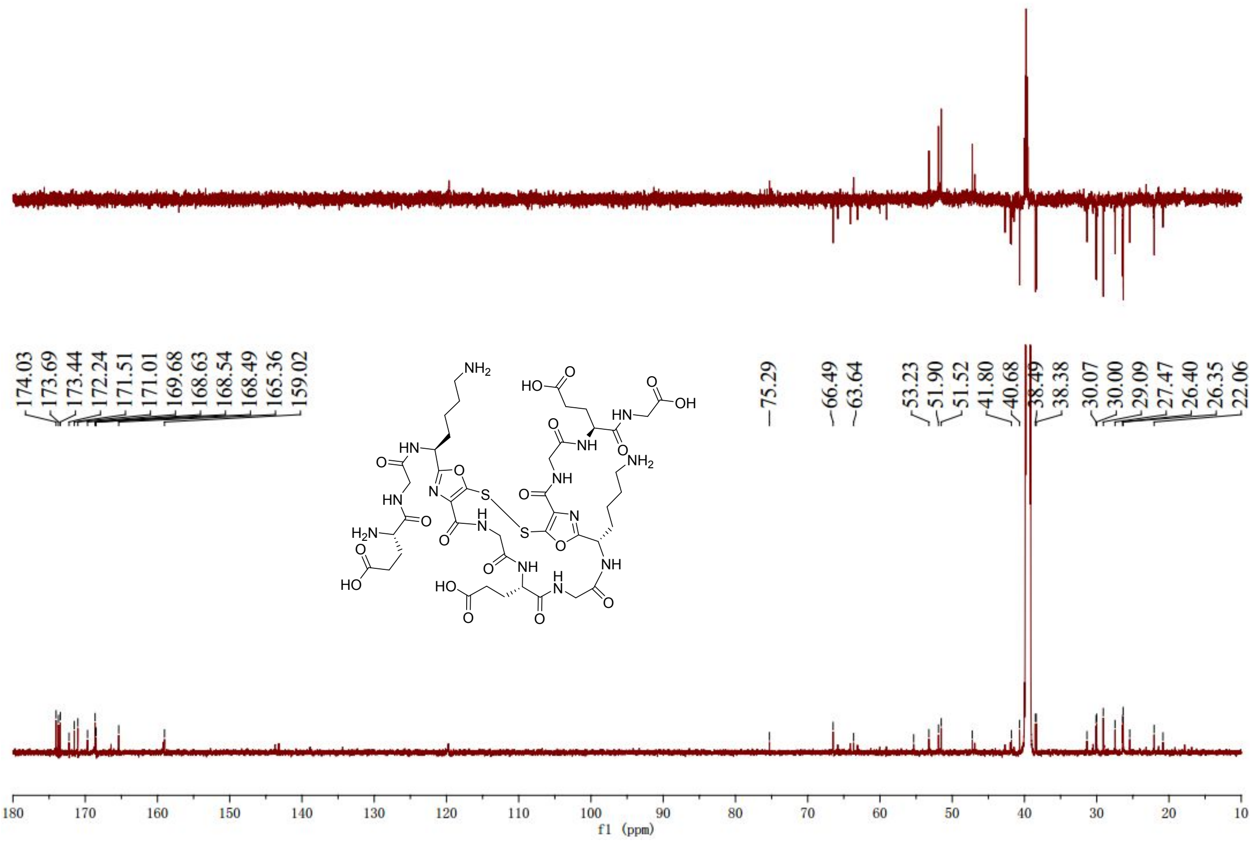

Figure S20: The ^{13}C NMR and DEPT spectra of oxidized bulbicupramide (176 MHz, DMSO-d_6).

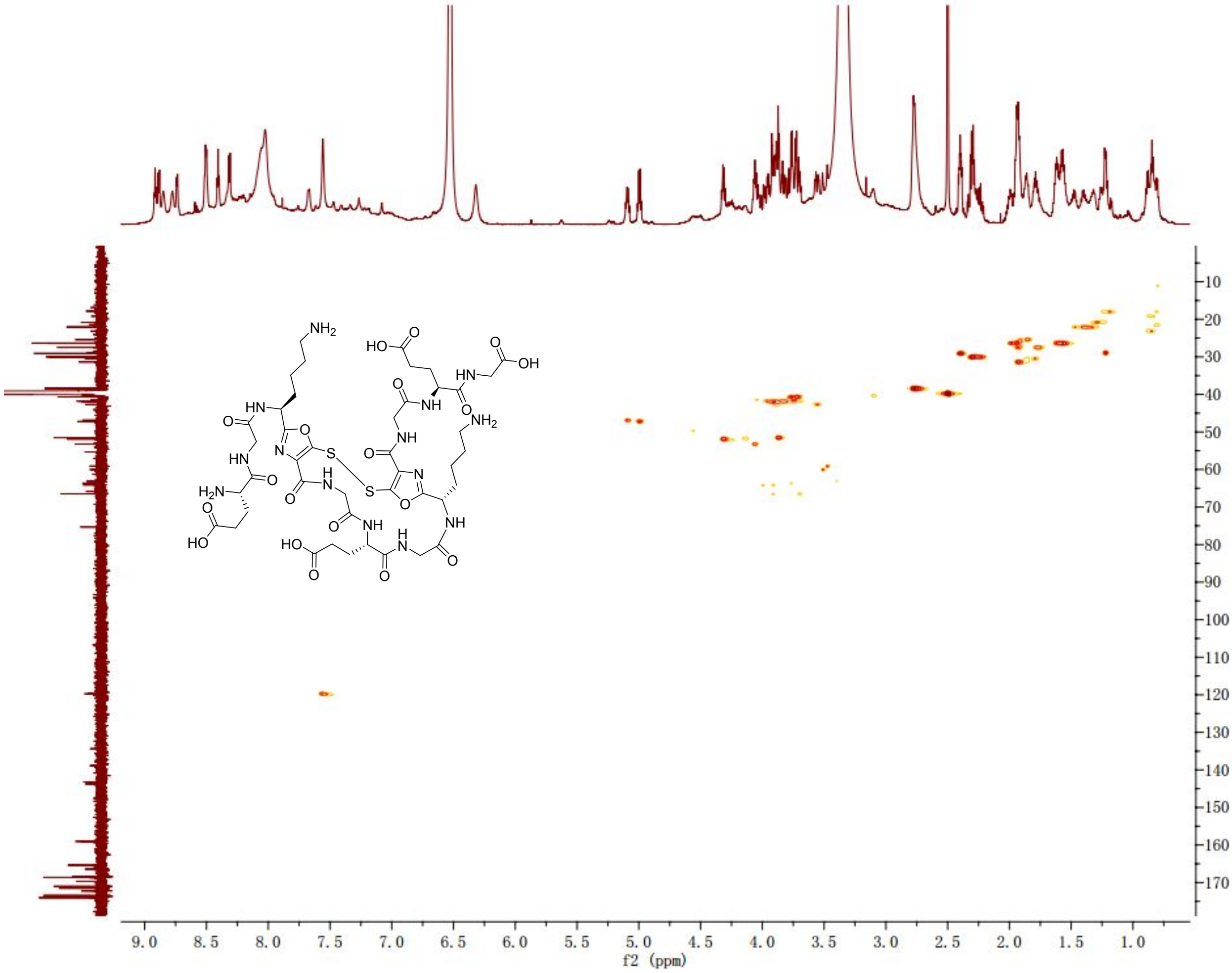

Figure S21: 2D ^{1}H-^{13}C HSQC spectrum of oxidized bulbicupramide (700 MHz, DMSO-d_6).

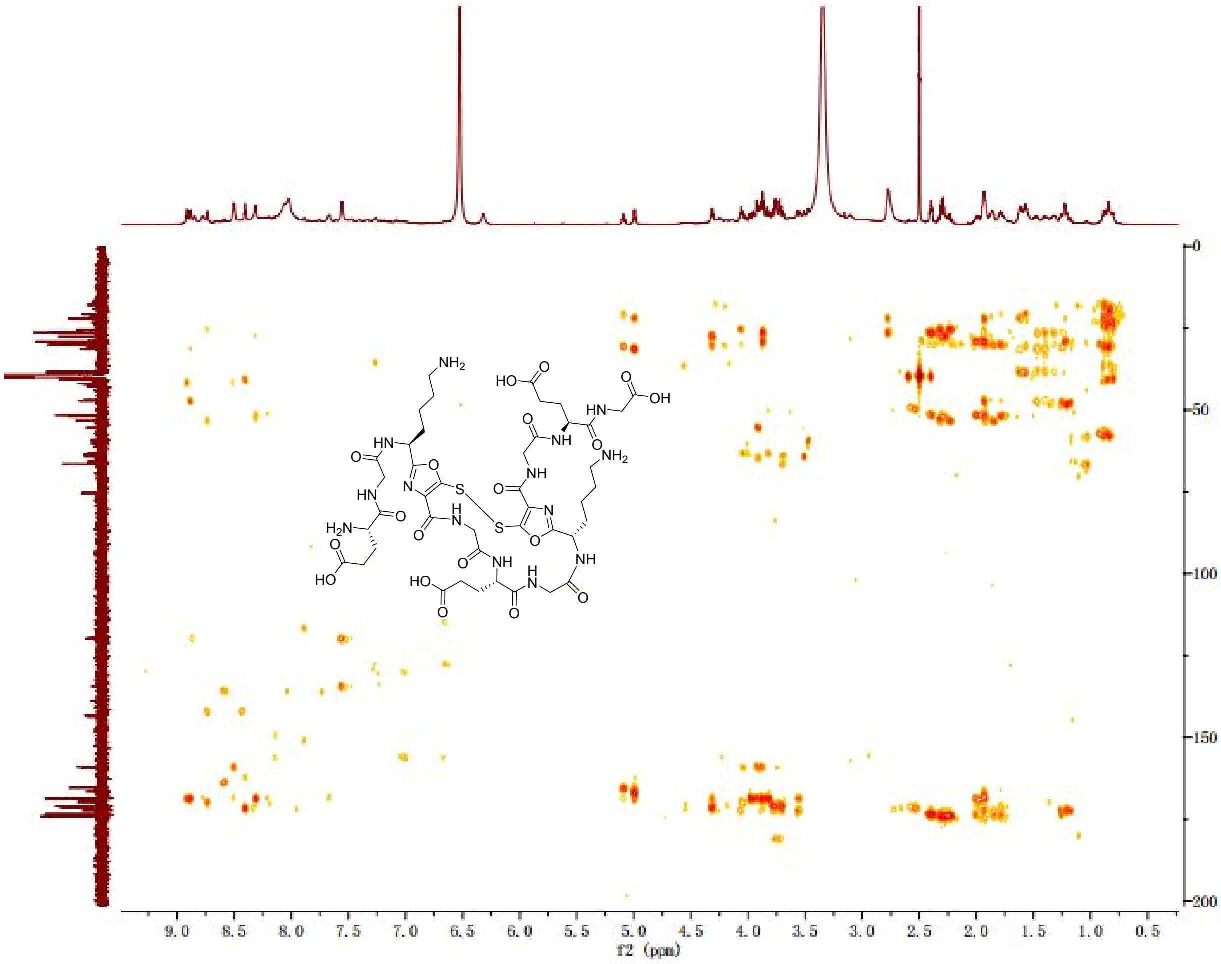

Figure S22: 2D ^{1}H-^{13}C HMBC spectrum of oxidized bulbicupramide (700 MHz, DMSO-d_6).

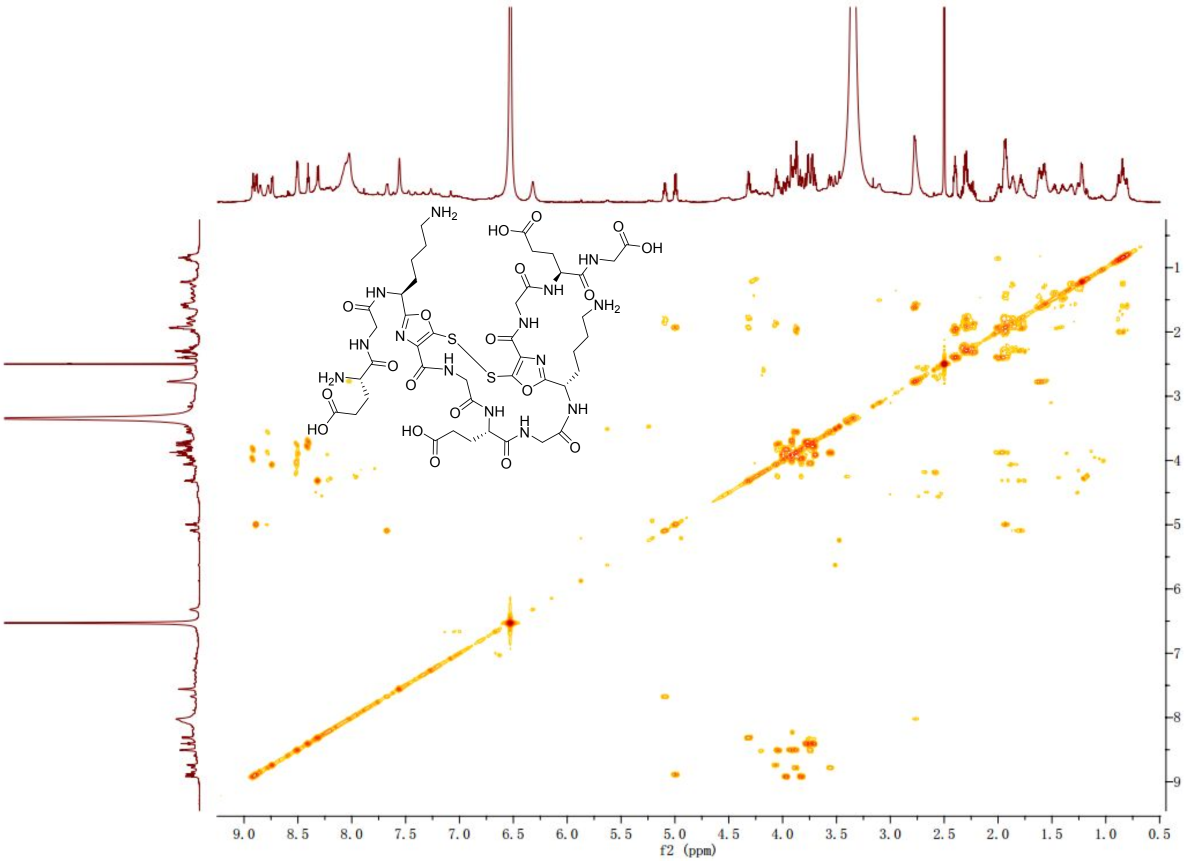

Figure S23: 2D ^{1}H-^{1}H COSY spectrum of oxidized bulbicupramide (700 MHz, DMSO-d_6).

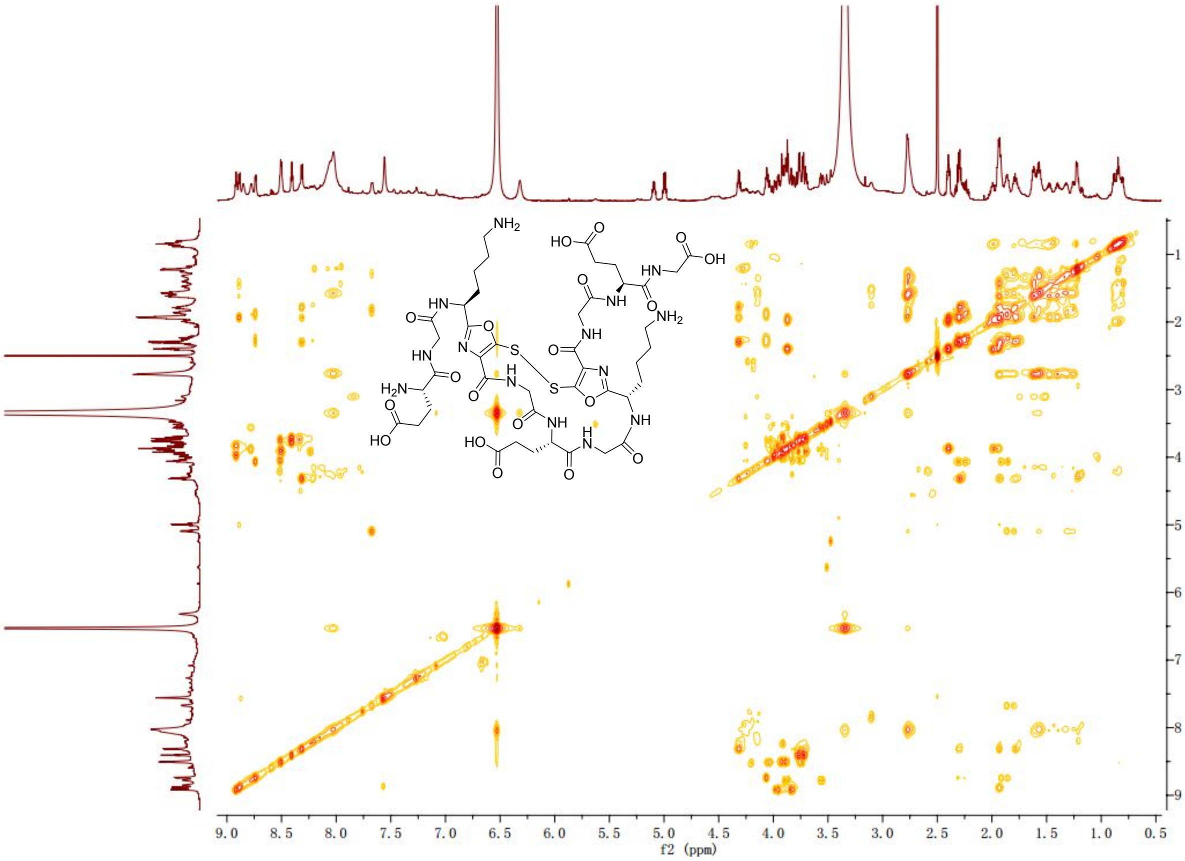

Figure S24: 2D ^{1}H-^{1}H TOCSY spectrum of oxidized bulbicupramide (700 MHz, DMSO-d_6).

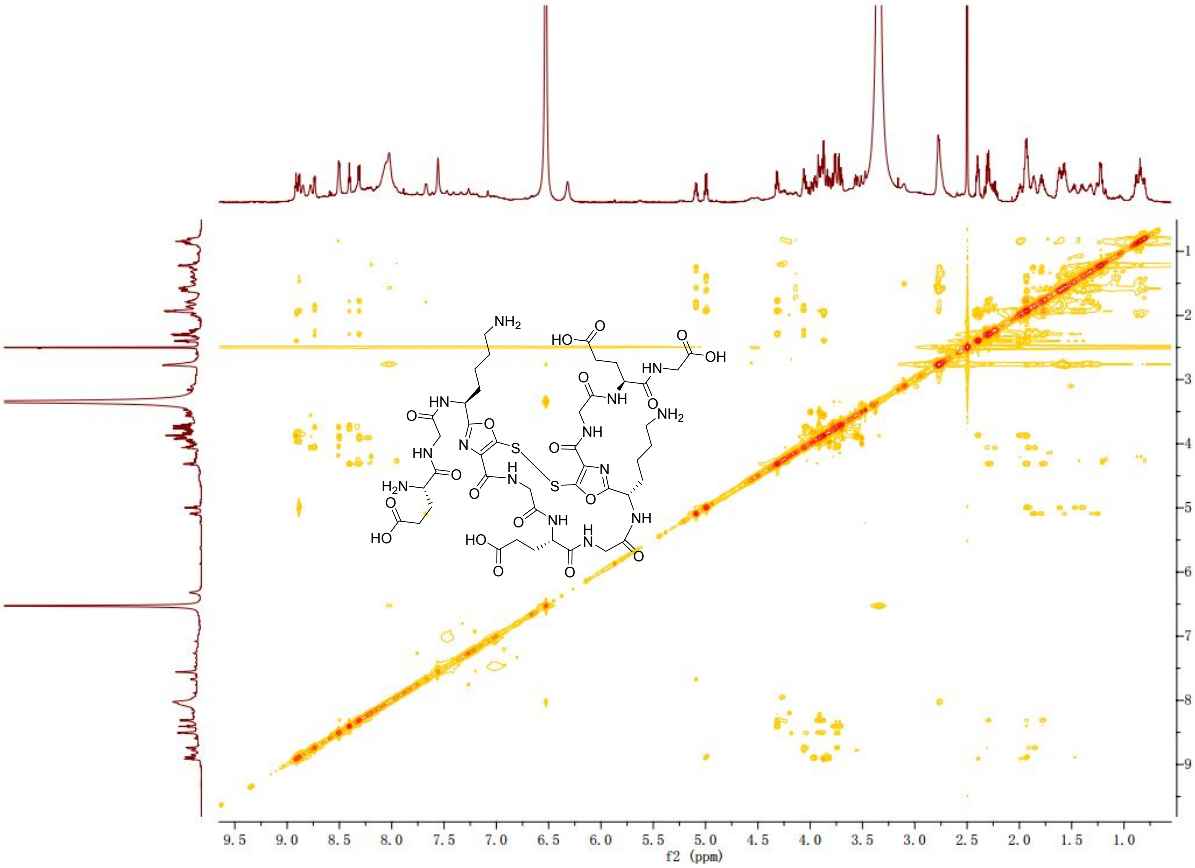

Figure S25: 2D ^1H-^1H ROESY spectrum of oxidized bulbicupramide (700 MHz, DMSO-d_6).

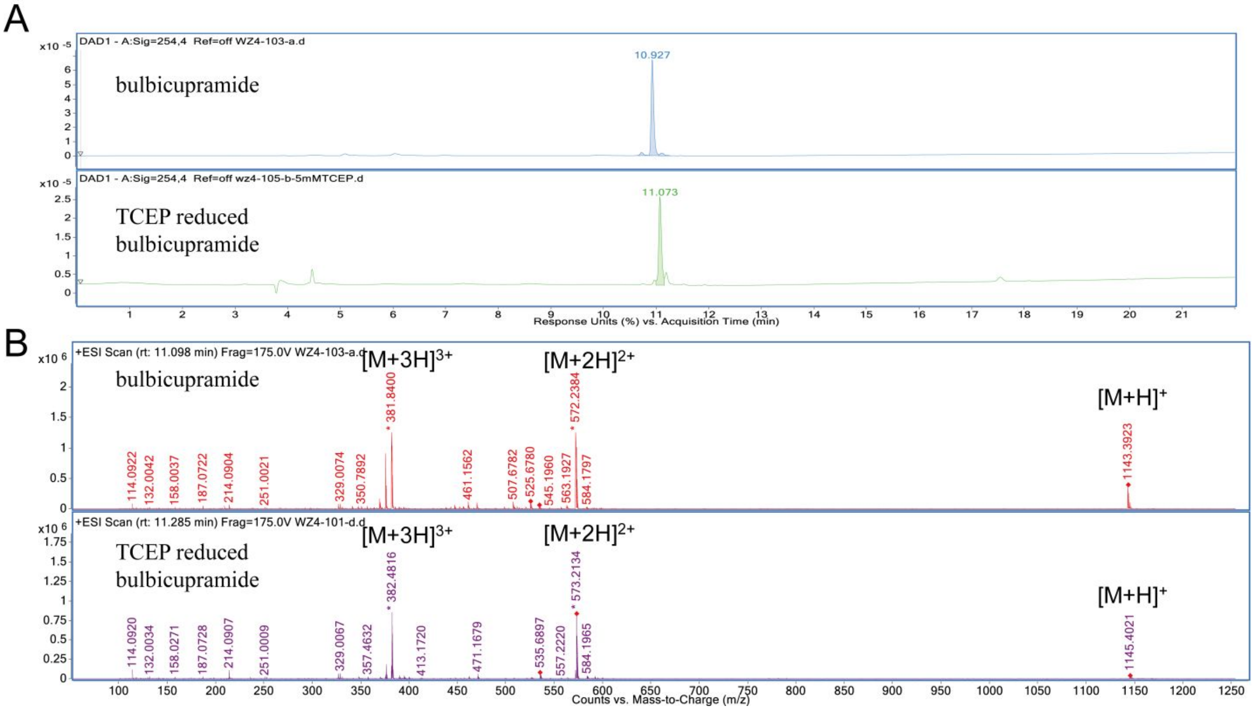

Figure S26: (A) Comparison of retention times of oxidized bulbicupramide and TCEP-reduced bulbicupramide. UV absorbance chromatograms were recorded at 254 nm wavelength. **(B)** Comparison of MS^1 spectra of oxidized bulbicupramide and TCEP-reduced bulbicupramide.

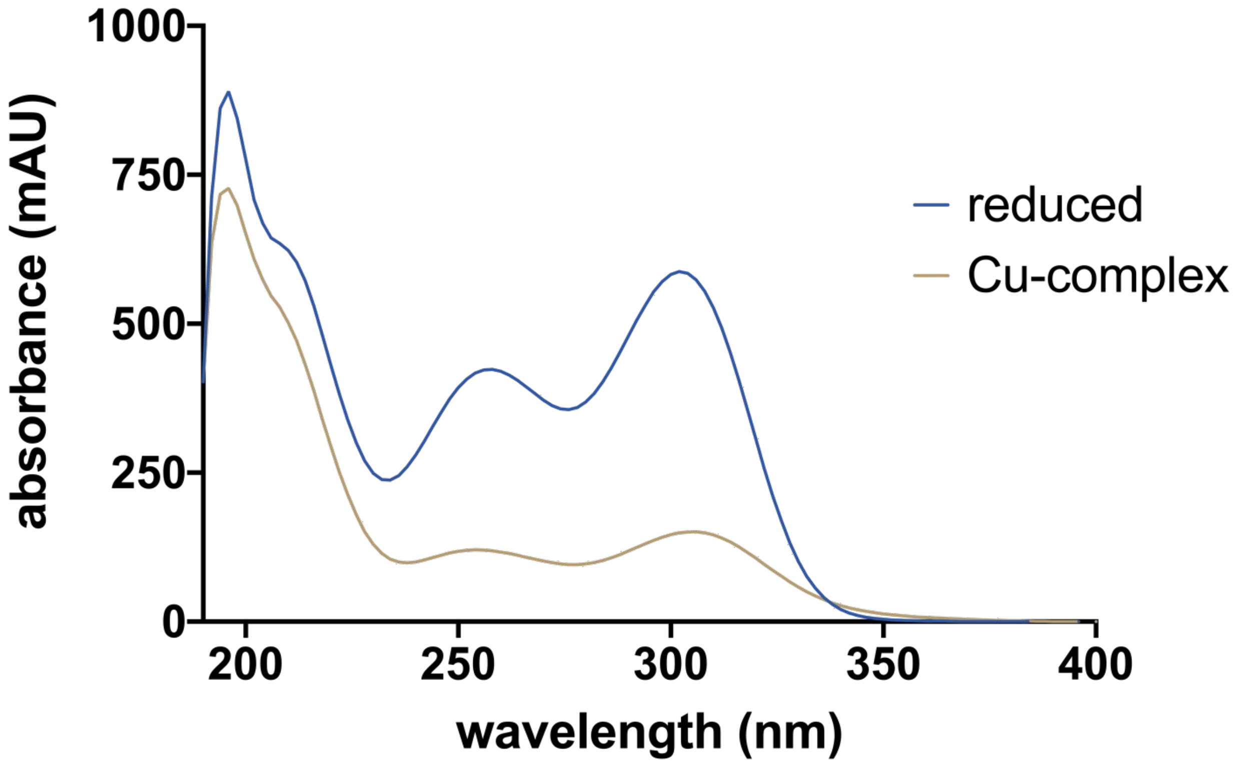

Figure S27: UV-Vis absorbance spectra for reduced form of bulbicupramide (blue curve) and reduced bulbicupramide when complexed with Cu(I) (gold curve). To generate the Cu(I)-complex, TCEP-reduced bulbicupramide was incubated with excess ascorbate and CuSO4, followed by chromatographic removal of excess ascorbate and CuSO4 by passage through a C18 stationary phase prior to UV-Vis absorbance data acquisition.

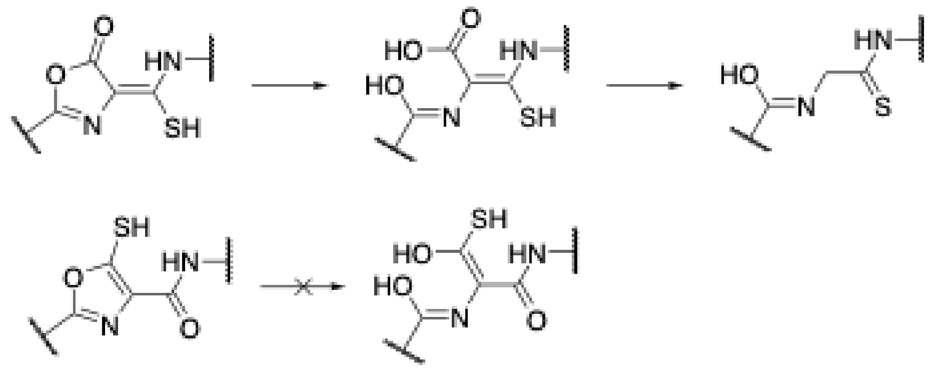

Figure S28: Proposed mechanism for decarboxylation of oxazolone-thioamide RiPPs. The thiooxazoles likely do not undergo the same degradation.

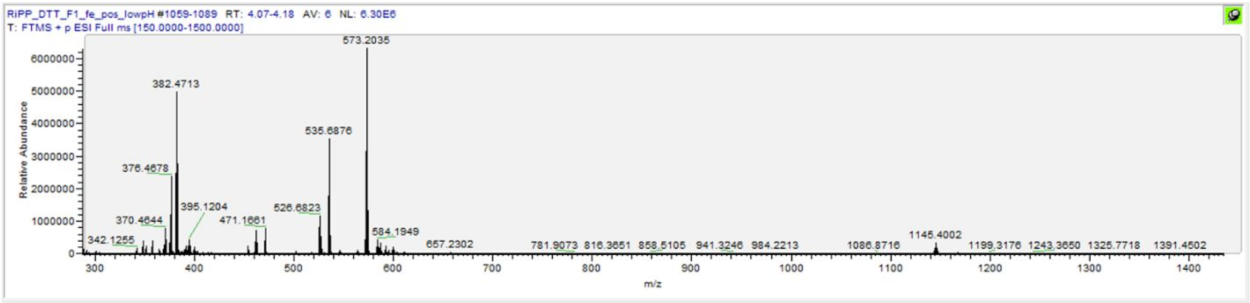

Figure S29: High resolution MS[1] spectra for reduced bulbicupramide recorded under no metal-infusion conditions. Raw data can be found here.

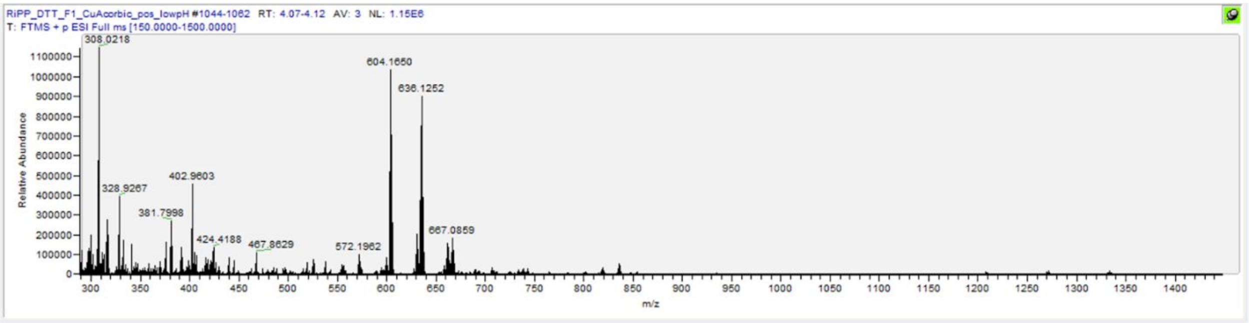

Figure S30: High resolution MS[1] spectra for reduced bulbicupramide recorded in a mass spectrometry experiments under conditions of Cu(I) infusion. Raw data can be found here.

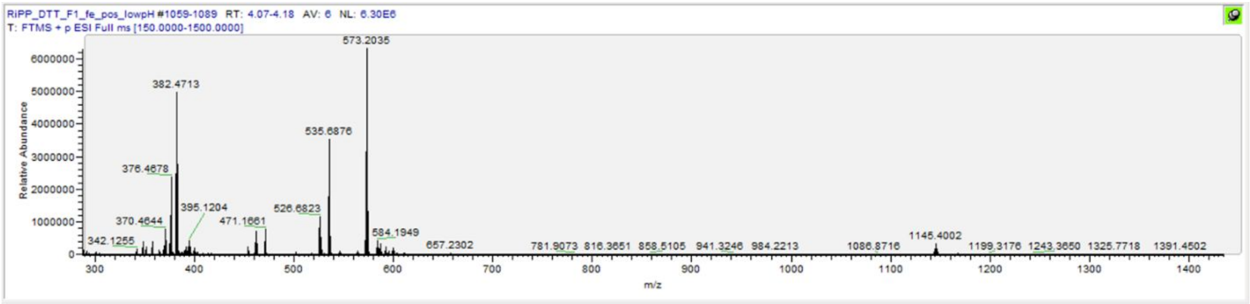

Figure S31: High resolution MS1 spectra for reduced bulbicupramide recorded in a mass spectrometry experiments under conditions of Fe(III) infusion. Raw data can be found here.

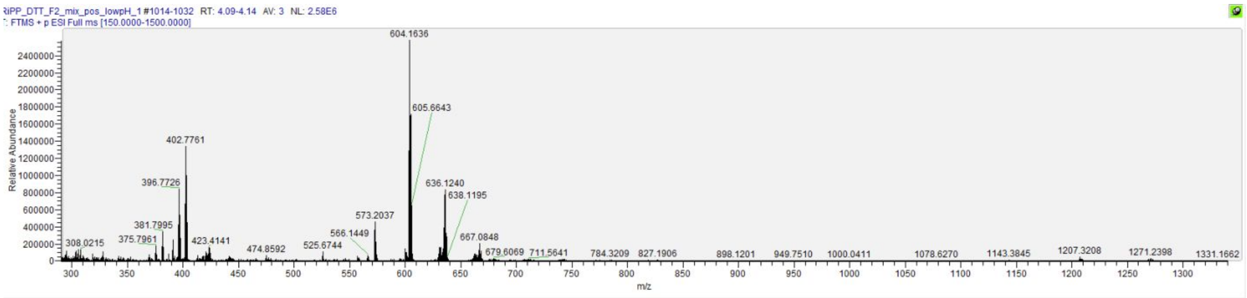

Figure S32: Metal-infusion competition experiment between Cu(I) and Ni(II), Zn(II), and Mn(II) reveals that bulbicupramide binds Cu(I) while not appreciably binding the other metals tested. High resolution MS^1 spectra for reduced bulbicupramide recorded in a mass spectrometry experiment under conditions of mixed metal infusion in one technical replicate. The MS^1 integrated over retention time 4.09–4.14 sec.

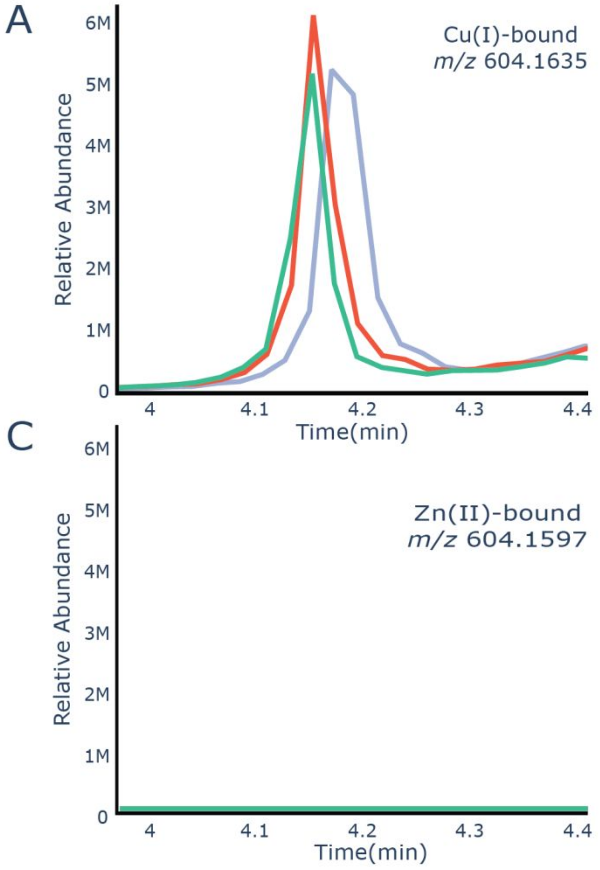

Figure S33: EICs resulting from three technical replicates (replicates shown in red, blue, and green) of a metal-infusion competition experiment between Cu(I) and Ni(II), Zn(II), and Mn(II), plotted with a 5 ppm tolerance. (**A**) EIC for reduced bulbicupramide and in complex with Cu(I), (**B**) in complex with Mn(II), (**C**) in complex with Zn(II), and (**D**) in complex with Ni(II). In the metal competition experiment, only Cu(I)-binding is observed (panel A), while adducts with other three metal ions are not detected in comparative high abundance (panels B–C). Interactive data can be visualized here.

	[CuSO4]		
	0 mM	0.1 mM	0.2 mM
**RecA**	1.E+09	1.E+08	1.E+08
**GAPDH**	6.E+09	7.E+09	3.E+09
**MreB**	1.E+08	2.E+07	3.E+07
**MmrC**	1.E+07	0.E+00	0.E+00
**MmrB**	0.E+00	0.E+00	0.E+00
**MmrA**	0.E+00	0.E+00	0.E+00

Figure S34: Intensities of housekeeping proteins RecA, GAPDH, and MreB, and of MmrA–C detected in proteomes of *Microbulbifer* sp. VAAF005 when exposed to 0 mM, 0.1 mM, and 0.2 mM $CuSO_4$.

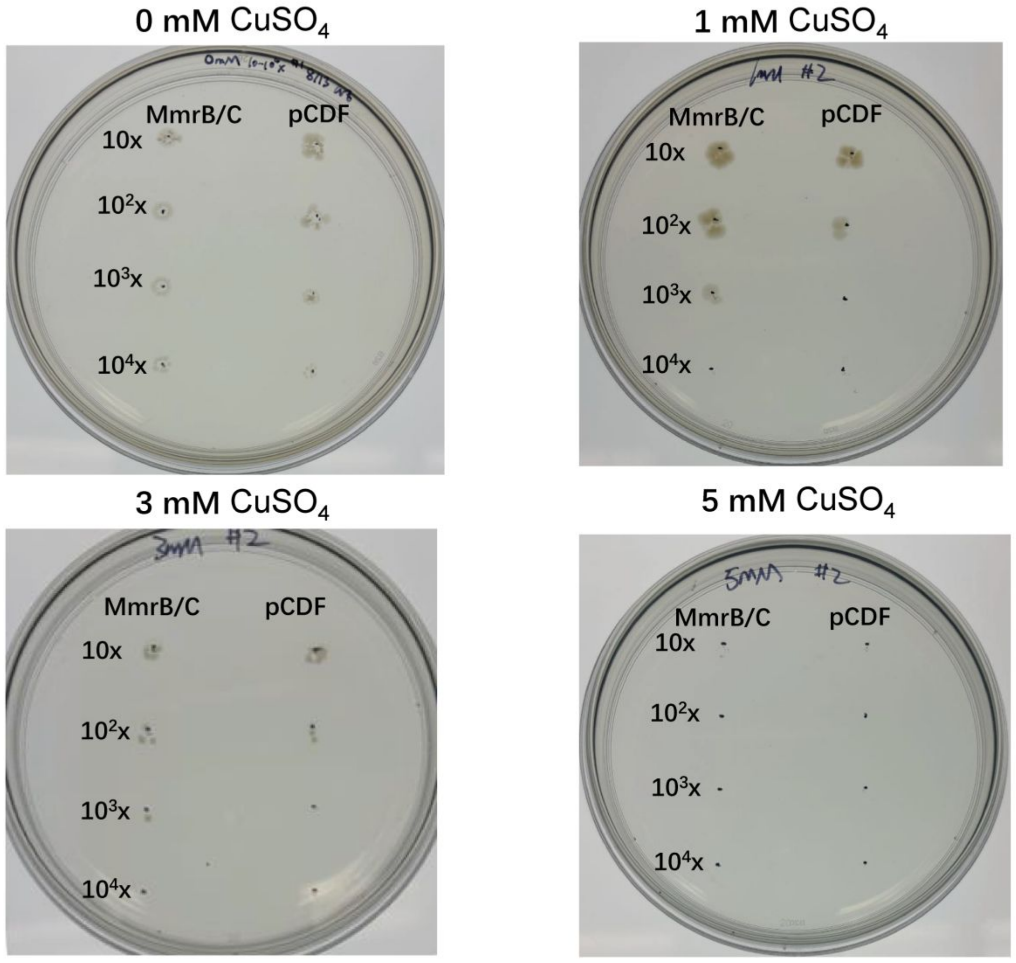

Figure S35: Solid copper toxicity assay. Spot tests using serially diluted *E*. coli cultures when *mmrA* was coexpressed with *mmrB* and *mmrC* (vertical columns marked 'MmrB/C') or when *mmrA* was expressed alone (vertical columns marked 'pCDF'; pCDF-Duet was the plasmid backbone used to express *mmrB* and *mmrC* and in this column, the wild type unmodified pCDF-Duet plasmid was included to provide a proper control) in presence of different Cu(II) concentrations.

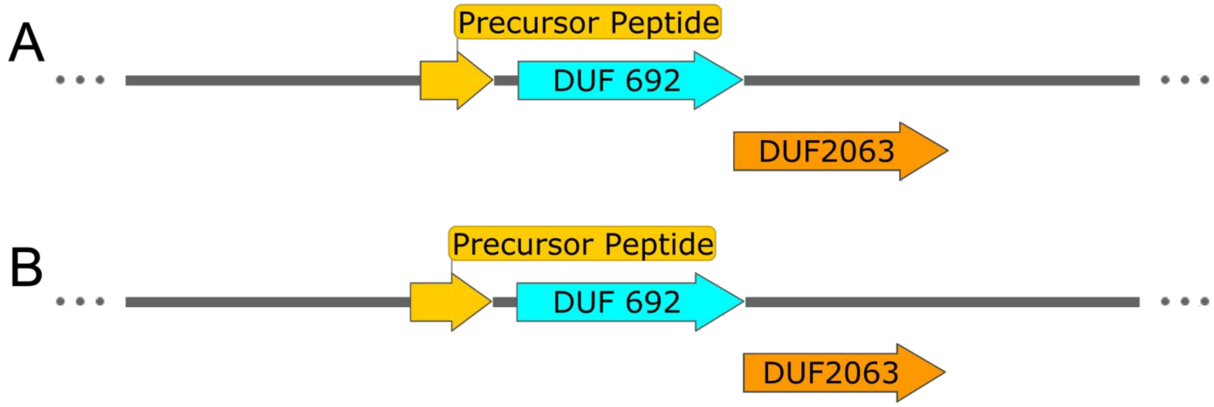

Figure S36: Putative chalkophore encoding BGCs identified in genomes of (**A**) *Pseudovibrio brasiliensis* strain Ab134, and (**B**) *Ruegeria spongiae* strain 2012CJ41-6. Both these strains were isolated from marine sponges.[60, 61] The RiPP precursor peptides do not bear the three-cassette architecture.

Sequence of the *Pseudovibrio brasiliensis* strain Ab134 precursor peptide:

MSKKTLSAAVLATAVTAAVAGANITVAEAASKEKCYGVSLAGENDCKAGAGTTCAGTSTVDY
QGNAWTLVPAGTCESIEVPGDRKGSLTELSRDLPK

Sequence of the *Ruegeria spongiae* strain 2012CJ41-6 precursor peptide:

MSNSAKTLAVAGAVAAALTASVTTTAHAQAKEKCYGVSLAGQNDCAAGPGTTCAGTSTVDYQ
GNAWTLVDAGTCDKIELPKTADGGARAGSLEPLDRDLPA

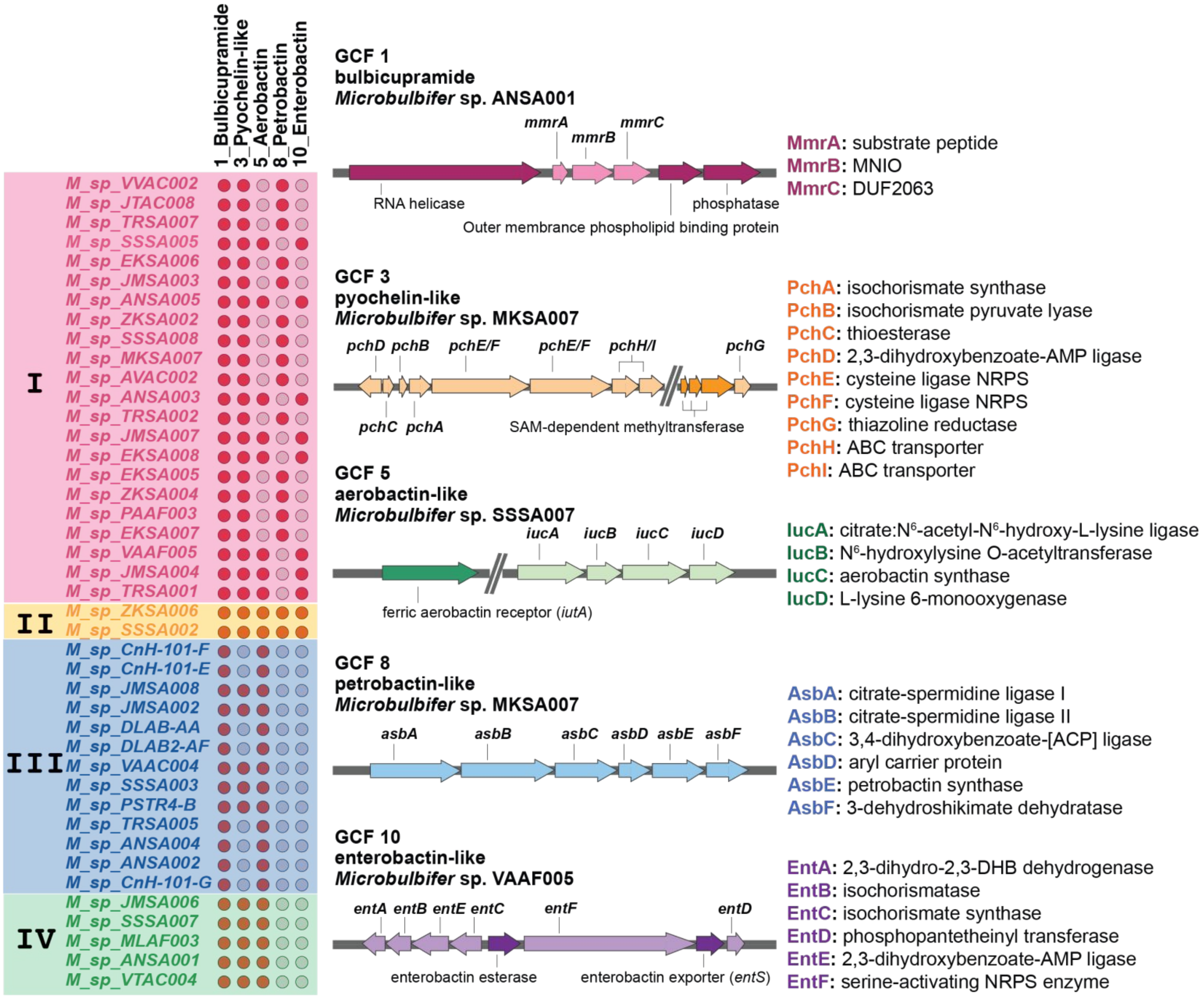

Figure S37: Species-specific inventory of metallophore BGCs encoded in 42 *Microbulbifer* genomes sequenced in this study illustrated as a GCF presence/absence matrix. The GCF numbering, assigned by BiG-SCAPE, is preserved from Figure 3.

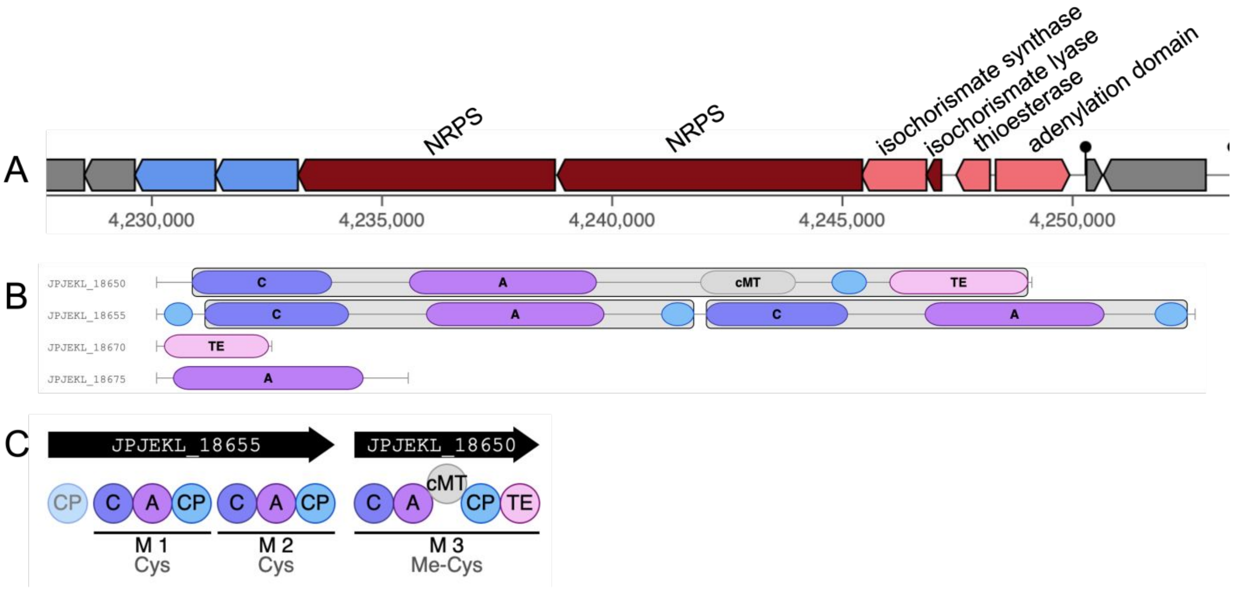

Figure S38: (**A**) The gene organization of a NRPS-encoding BGC identified by antiSMASH in the genome of *Microbulbifer* sp. VAAC004. The annotation of the core biosynthetic genes is denoted. The standalone adenylation domain is annotated by antiSMASH to activate hydroxybenzoic acids, such as salicylic acid, that is likely to be derived from chorismite by the collective activity of isochorismate synthase and isochrismate lyase encoded in this BGC. (**B**) The NRPS domain organization as predicted by antiSMASH. Each of the three condensation domains are predicted to be heterocyclizing condensation domains. (**C**) The three collinear adenylation domains are each predicted to activate Cys. In addition to the thioesterase domain embedded within the NRPS assembly line, there is an additional thioesterase encoded in the BGC (annotated in panel A). The standalone thioesterase may perform a proofreading role.

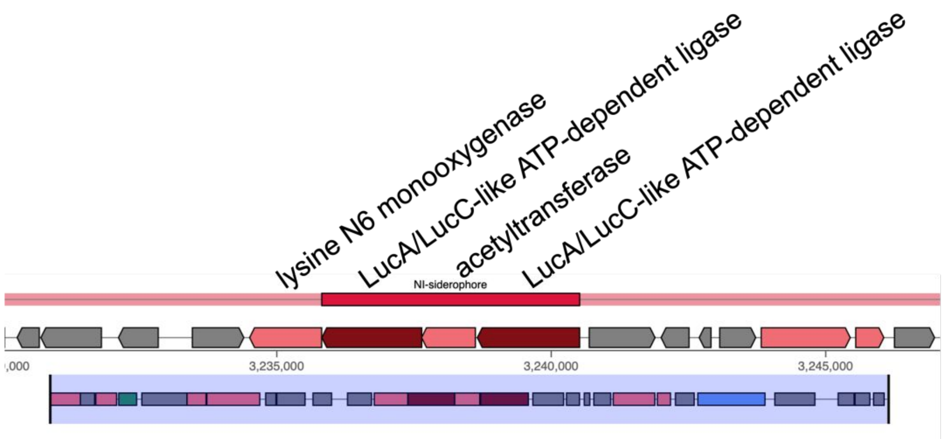

Figure S39: Illustrative hydroxamate siderophore encoding BGC identified in the genome of *Microbulbifer* sp. ANSA001. The annotations of key siderophore biosynthetic genes are illustrated.

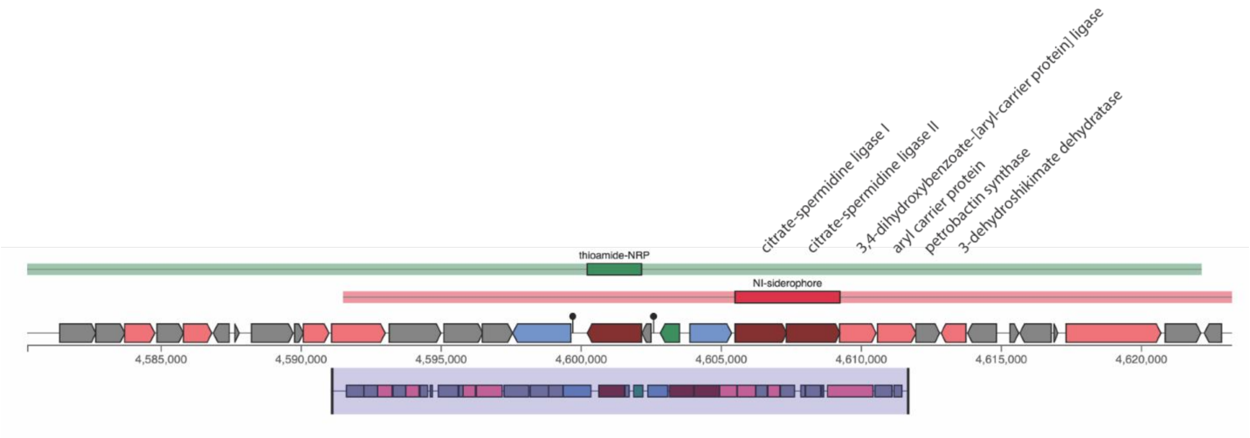

Figure S40: Annotation of the petrobactin-like siderophore encoding BGC identified in the genome of *Microbulbifer* sp. MKSA007. The annotation of the key siderophore genes (*asbABCDEF*) are illustrated.

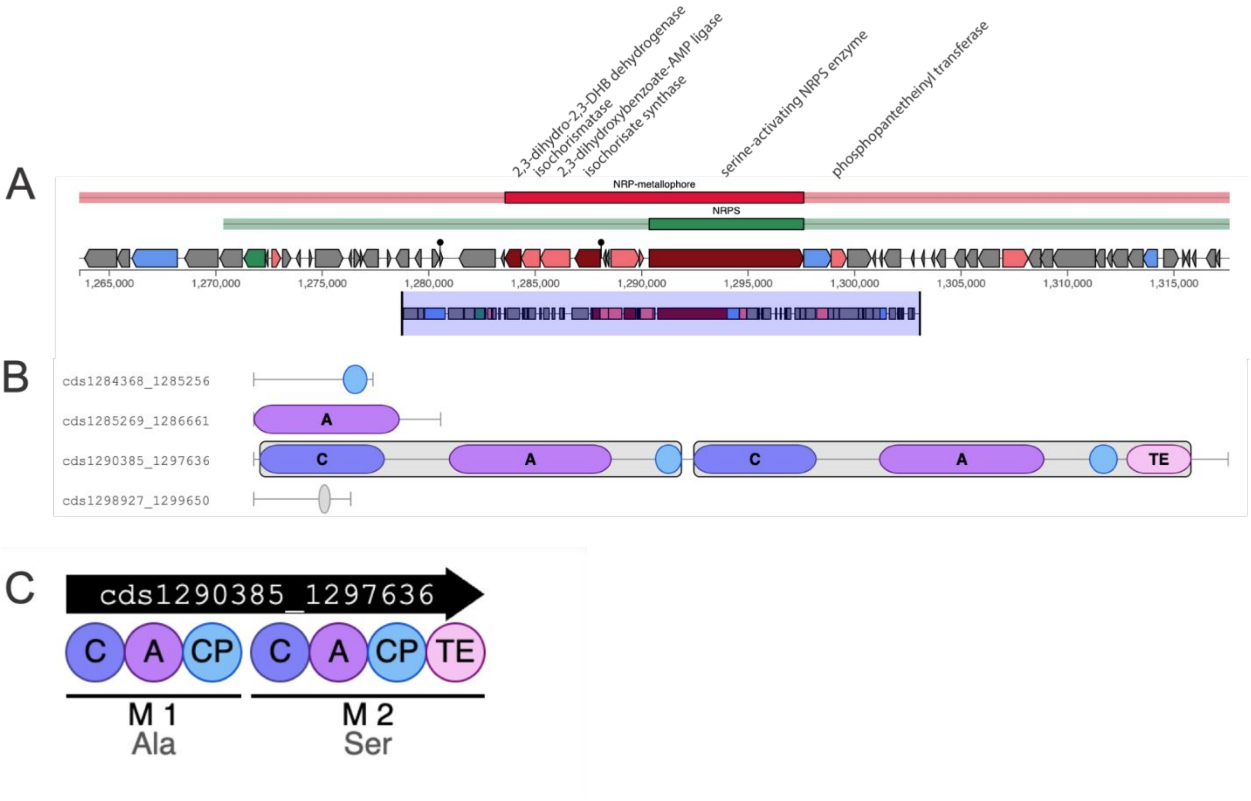

Figure S41: (A) Annotation of the enterobactin-like siderophore encoding BGC identified in the genome of *Microbulbifer* sp. VAAF005. The annotation of the key siderophore genes (*entABCDEF*) are illustrated. (**B**) The NRPS domain organization as predicted by antiSMASH. (**C**) The adenylation domain specificities as predicted by antiSMASH.

SUPPLEMENTARY REFERENCES

1. Seemann, T. barrnap 0.9 : rapid ribosomal RNA prediction. https://github.com/tseemann/barrnap

2. Katoh, K.; Standley, D. M., MAFFT multiple sequence alignment software version 7: improvements in performance and usability. *Mol Biol Evol* **2013,** *30*, 772-80.

3. Minh, B. Q.; Schmidt, H. A.; Chernomor, O.; Schrempf, D.; Woodhams, M. D.; von Haeseler, A.; Lanfear, R., IQ-TREE 2: New Models and Efficient Methods for Phylogenetic Inference in the Genomic Era. *Molecular Biology and Evolution* **2020,** *37*, 1530-1534.

4. Kalyaanamoorthy, S.; Minh, B. Q.; Wong, T. K. F.; Von Haeseler, A.; Jermiin, L. S., ModelFinder: fast model selection for accurate phylogenetic estimates. *Nature Methods* **2017,** *14*, 587-589.

5. Minh, B. Q.; Nguyen, M. A.; von Haeseler, A., Ultrafast approximation for phylogenetic bootstrap. *Mol Biol Evol* **2013,** *30*, 1188-95.

6. Hoang, D. T.; Chernomor, O.; von Haeseler, A.; Minh, B. Q.; Vinh, L. S., UFBoot2: Improving the Ultrafast Bootstrap Approximation. *Mol Biol Evol* **2018,** *35*, 518-522.

7. Guindon, S.; Dufayard, J. F.; Lefort, V.; Anisimova, M.; Hordijk, W.; Gascuel, O., New algorithms and methods to estimate maximum-likelihood phylogenies: assessing the performance of PhyML 3.0. *Syst Biol* **2010,** *59*, 307-21.

8. Naser-Khdour, S.; Minh, B. Q.; Lanfear, R., Assessing Confidence in Root Placement on Phylogenies: An Empirical Study Using Non-Reversible Models for Mammals. Cold Spring Harbor Laboratory2020.

9. Rambaut, A. FigTree v1.4.4. http://tree.bio.ed.ac.uk/software/figtree/

10. Davis, E. W.; Okrent, R. A.; Manning, V. A.; Trippe, K. M., Unexpected distribution of the 4-formylaminooxyvinylglycine (FVG) biosynthetic pathway in Pseudomonas and beyond. *PLOS ONE* **2021,** *16*, e0247348.

11. Jonkheer, E. M.; Van Workum, D.-J. M.; Sheikhizadeh Anari, S.; Brankovics, B.; De Haan, J. R.; Berke, L.; Van Der Lee, T. A. J.; De Ridder, D.; Smit, S., PanTools v3: functional annotation, classification and phylogenomics. *Bioinformatics* **2022,** *38*, 4403-4405.

12. Jain, C.; Rodriguez-R, L. M.; Phillippy, A. M.; Konstantinidis, K. T.; Aluru, S., High throughput ANI analysis of 90K prokaryotic genomes reveals clear species boundaries. *Nature Communications* **2018,** *9*.

13. Ondov, B. D.; Treangen, T. J.; Melsted, P.; Mallonee, A. B.; Bergman, N. H.; Koren, S.; Phillippy, A. M., Mash: fast genome and metagenome distance estimation using MinHash. *Genome Biology* **2016,** *17*.

14. Waskom, M., seaborn: statistical data visualization. *Journal of Open Source Software* **2021,** *6*, 3021.

15. Navarro-Muñoz, J. C.; Selem-Mojica, N.; Mullowney, M. W.; Kautsar, S. A.; Tryon, J. H.; Parkinson, E. I.; De Los Santos, E. L. C.; Yeong, M.; Cruz-Morales, P.; Abubucker, S.; Roeters, A.; Lokhorst, W.; Fernandez-Guerra, A.; Cappelini, L. T. D.; Goering, A. W.; Thomson, R. J.; Metcalf, W. W.; Kelleher, N. L.; Barona-Gomez, F.; Medema, M. H., A computational framework to explore large-scale biosynthetic diversity. *Nature Chemical Biology* **2020,** *16*, 60-68.

16. Aron, A. T.; Petras, D.; Schmid, R.; Gauglitz, J. M.; Büttel, I.; Antelo, L.; Zhi, H.; Nuccio, S.-P.; Saak, C. C.; Malarney, K. P.; Thines, E.; Dutton, R. J.; Aluwihare, L. I.; Raffatellu, M.; Dorrestein, P. C., Native mass spectrometry-based metabolomics identifies metal-binding compounds. *Nature Chemistry* **2022,** *14*, 100-109.

17. Xiao, H.; Chen, W.; Smeekens, J. M.; Wu, R., An enrichment method based on synergistic and reversible covalent interactions for large-scale analysis of glycoproteins. *Nat. Commun.* **2018,** *9*, 1692.

18. Smeekens, J. M.; Xiao, H.; Wu, R., Global Analysis of Secreted Proteins and Glycoproteins in Saccharomyces cerevisiae. *J. Proteome Res.* **2017,** *16*, 1039-1049.

19. Xu, S.; Yin, K.; Xu, X.; Fu, L.; Wu, R., O-GlcNAcylation reduces proteome solubility and regulates the formation of biomolecular condensates in human cells. *Nat. Commun.* **2025,** *16*, 4068.

20. Chambers, M. C.; Maclean, B.; Burke, R.; Amodei, D.; Ruderman, D. L.; Neumann, S.; Gatto, L.; Fischer, B.; Pratt, B.; Egertson, J.; Hoff, K.; Kessner, D.; Tasman, N.; Shulman, N.; Frewen, B.; Baker, T. A.; Brusniak, M. Y.; Paulse, C.; Creasy, D.; Flashner, L.; Kani, K.; Moulding, C.; Seymour, S. L.; Nuwaysir, L. M.; Lefebvre, B.; Kuhlmann, F.; Roark, J.; Rainer, P.; Detlev, S.; Hemenway, T.; Huhmer, A.; Langridge, J.; Connolly, B.; Chadick, T.; Holly, K.; Eckels, J.; Deutsch, E. W.; Moritz, R. L.; Katz, J. E.; Agus, D. B.; MacCoss, M.; Tabb, D. L.; Mallick, P., A cross-platform toolkit for mass spectrometry and proteomics. *Nat. Biotechnol.* **2012,** *30*, 918-20.

21. Kong, A. T.; Leprevost, F. V.; Avtonomov, D. M.; Mellacheruvu, D.; Nesvizhskii, A. I., MSFragger: ultrafast and comprehensive peptide identification in mass spectrometry-based proteomics. *Nat. Methods* **2017,** *14*, 513-520.

22. Park, S.; Yoon, S. Y.; Ha, M.-J.; Yoon, J.-H., Microbulbifer aestuariivivens sp. nov., isolated from a tidal flat. *International journal of systematic and evolutionary microbiology.* **2017,** *67*, 1436-1441.

23. Moh, T. H.; Lau, N.-S.; Furusawa, G.; Amirul, A.-A. A., Complete genome sequence of Microbulbifer sp. CCB-MM1, a halophile isolated from Matang Mangrove Forest, Malaysia. *Standards in Genomic Sciences* **2017,** *12*, 36.

24. Long, M.; Chen, D.; Fan, H.; Tang, S.; Gan, Z.; Xia, H.; Lu, Y., Microbulbifer bruguierae sp. nov., isolated from sediment of mangrove plant Bruguiera sexangula, and comparative genomic analyses of the genus Microbulbifer. *International Journal of Systematic and Evolutionary Microbiology* **2024,** *74*, 006209.

25. Yoon, J.-H.; Jung, S.-Y.; Kang, S.-J.; Oh, T.-K., Microbulbifer celer sp. nov., isolated from a marine solar saltern of the Yellow Sea in Korea. *International Journal of Systematic and Evolutionary Microbiology* **2007,** *57*, 2365-2369.

26. Wang, C. S.; Wang, Y.; Xu, X. W.; Zhang, D. S.; Wu, Y. H.; Wu, M., Microbulbifer donghaiensis sp. nov., isolated from marine sediment of the East China Sea. *International journal of systematic and evolutionary microbiology.* **2009,** *59*, 545-549.

27. Lee, J.-Y.; Kim, P. S.; Hyun, D.-W.; Kim, H. S.; Shin, N.-R.; Jung, M.-J.; Yun, J.-H.; Kim, M.-S.; Whon, T. W.; Bae, J.-W., Microbulbifer echini sp. nov., isolated from the gastrointestinal tract of a purple sea urchin, Heliocidaris crassispina. *International journal of systematic and evolutionary microbiology.* **2017,** *67*, 998-1004.

28. Yoon, J.-H.; Kim, H.; Kang, K. H.; Oh, T.-K.; Park, Y.-H., Transfer of Pseudomonas elongata Humm 1946 to the genus Microbulbifer as Microbulbifer elongatus comb. nov. *International journal of systematic and evolutionary microbiology.* **2003,** *53*, 1357-1361.

29. Nishijima, M.; Takadera, T.; Imamura, N.; Kasai, H.; An, K. D.; Adachi, K.; Nagao, T.; Sano, H.; Yamasato, K., Microbulbifer variabilis sp. nov. and Microbulbifer epialgicus sp. nov., isolated from Pacific marine algae, possess a rod-coccus cell cycle in association with the growth phase. *International journal of systematic and evolutionary microbiology.* **2009,** *59*, 1696-1707.

30. Xiong, Q.; Wang, D.; Dong, X.; Liu, D.; Liu, Y.; Li, P.; Wu, G.; Luo, Y.; Zhang, R.; Liu, S.; Zhang, G., Microbulbifer flavimaris sp. nov., a halophilic Gammaproteobacteria isolated from marine sediment of the Yellow Sea, China. *International journal of systematic and evolutionary microbiology.* **2019,** *69*, 1135-1141.

31. Fu, Y.-H.; Wu, Z.-C.; Kong, Y.-H.; Xu, X.-W.; Sun, C.; Wu, Y.-H., Microbulbifer zhoushanensis sp. nov., Microbulbifer sediminum sp. nov. and Microbulbifer guangxiensis sp. nov., three marine bacteria isolated from a tidal flat. *International journal of systematic and evolutionary microbiology.* **2023,** *73*.

32. Tang, S. K.; Wang, Y.; Cai, M.; Lou, K.; Mao, P. H.; Jin, X.; Jiang, C. L.; Xu, L. H.; Li, W. J., Microbulbifer halophilus sp. nov., a moderately halophilic bacterium from north-west China. *International journal of systematic and evolutionary microbiology.* **2008,** *58*, 2036-2040.

33. Huang, H.; Mo, K.; Hu, Y.; Liu, M.; Zhu, J.; Zou, X.; Bao, S., Microbulbifer harenosus sp. nov., an alginate-degrading bacterium isolated from coastal sand. *International journal of systematic and evolutionary microbiology.* **2020,** *70*, 1639-1643.

34. Li, Z.; Wei, R.; Gao, M.; Ren, Y.; Yu, B.; Nie, K.; Xu, H.; Liu, L., Biodegradation of low-density polyethylene by *Microbulbifer hydrolyticus* IRE-31. *Journal of Environmental Management* **2020,** *263*, 110402.

35. Tao, L.-C.; Gao, J.-W.; Su, Y.; Zhang, W.-W.; Cao, K.; Yu, X.-Y.; Ma, K.-J.; Xu, L.; Xia, X.-M.; Sun, C., Microbulbifer magnicolonia sp. nov., isolated from sediment of tidal flat located in Zhoushan, China. *International journal of systematic and evolutionary microbiology.* **2024,** *74*.

36. Vashist, P.; Nogi, Y.; Ghadi, S. C.; Verma, P.; Shouche, Y. S., Microbulbifer mangrovi sp. nov., a polysaccharide-degrading bacterium isolated from an Indian mangrove. *International journal of systematic and evolutionary microbiology.* **2013,** *63*, 2532-2537.

37. Zhang, D.-S.; Huo, Y.-Y.; Xu, X.-W.; Wu, Y.-H.; Wang, C.-S.; Xu, X.-F.; Wu, M., Microbulbifer marinus sp. nov. and Microbulbifer yueqingensis sp. nov., isolated from marine sediment. *International journal of systematic and evolutionary microbiology.* **2012,** *62*, 505-510.

38. Jeong, S. H.; Yang, S.-H.; Jin, H. M.; Kim, J. M.; Kwon, K. K.; Jeon, C. O., Microbulbifer gwangyangensis sp. nov. and Microbulbifer pacificus sp. nov., isolated from marine environments. *International journal of systematic and evolutionary microbiology.* **2013,** *63*, 1335-1341.

39. Camacho, M.; del Carmen Montero-Calasanz, M.; Redondo-Gómez, S.; Rodríguez-Llorente, I.; Schumann, P.; Klenk, H.-P., Microbulbifer rhizosphaerae sp. nov., isolated from the rhizosphere of the halophyte Arthrocnemum macrostachyum. *International journal of systematic and evolutionary microbiology.* **2016,** *66*, 1844-1850.

40. Ishaq, N.; Zhang, M.; Gao, L.; Ilan, M.; Li, Z., Microbulbifer spongiae sp. nov., isolated from marine sponge Diacarnus erythraeanus. *International journal of systematic and evolutionary microbiology.* **2024,** *74*.

41. Kämpfer, P.; Arun, A. B.; Young, C.-C.; Rekha, P. D.; Martin, K.; Busse, H. J.; Chen, W.-M., Microbulbifer taiwanensis sp. nov., isolated from coastal soil. *International journal of systematic and evolutionary microbiology.* **2012,** *62*, 2485-2489.

42. Miyazaki, M.; Nogi, Y.; Ohta, Y.; Hatada, Y.; Fujiwara, Y.; Ito, S.; Horikoshi, K., Microbulbifer agarilyticus sp. nov. and Microbulbifer thermotolerans sp. nov., agar-degrading bacteria isolated from deep-sea sediment. *International journal of systematic and evolutionary microbiology.* **2008,** *58*, 1128-1133.

43. Baba, A.; Miyazaki, M.; Nagahama, T.; Nogi, Y., Microbulbifer chitinilyticus sp. nov. and Microbulbifer okinawensis sp. nov., chitin-degrading bacteria isolated from mangrove forests. *International journal of systematic and evolutionary microbiology.* **2011,** *61*, 2215-2220.

44. Yoon, J.-H.; Kim, I.-G.; Oh, T.-K.; Park, Y.-H., Microbulbifer maritimus sp. nov., isolated from an intertidal sediment from the Yellow Sea, Korea. *International Journal of Systematic and Evolutionary Microbiology* **2004,** *54*, 1111-1116.

45. Romanenko, L.; Kurilenko, V.; Otstavnykh, N.; Velansky, P.; Isaeva, M.; Mikhailov, V., Microbulbifer okhotskensis sp. nov., isolated from a deep bottom sediment of the Okhotsk Sea. *Archives of Microbiology* **2022,** *204*.

46. Stanier, R. Y., A Note on the Taxonomy of Proteus Hydrophilus. *Journal of Bacteriology* **1943,** *46*, 213-214.

47. Castellani, A.; Chalmers, A. J., *Manual of tropical medicine*. 3d ed.; Baillière, Tindall and Cox: London, 1919.

48. Pacini, F., *Osservazioni microscopiche e deduzioni patologiche sul cholera asiatico*. Firenze : Tipografia di Federigo Bencini: Gazette Medicale de Italiana Toscano Firenze 1854; Vol. 6.

49. Leprevost, L.; Jünger, S.; Lippens, G.; Guillaume, C.; Sicoli, G.; Oliveira, L.; Falcone, E.; de Santis, E.; Rivera-Millot, A.; Billon, G.; Stellato, F.; Henry, C.; Antoine, R.; Zirah, S.; Dubiley, S.; Li, Y.; Jacob-Dubuisson, F., A widespread family of ribosomal peptide metallophores involved in bacterial adaptation to metal stress. *Proceedings of the National Academy of Sciences* **2024,** *121*, e2408304121.

50. Lewis, J. K.; Jochimsen, A. S.; Lefave, S. J.; Young, A. P.; Kincannon, W. M.; Roberts, A. G.; Kieber-Emmons, M. T.; Bandarian, V., New Role for Radical SAM Enzymes in the Biosynthesis of Thio(seleno)oxazole RiPP Natural Products. *Biochemistry* **2021,** *60*, 3347-3361.

51. Manley, O. M.; Shriver, T. J.; Xu, T.; Melendrez, I. A.; Palacios, P.; Robson, S. A.; Guo, Y.; Kelleher, N. L.; Ziarek, J. J.; Rosenzweig, A. C., A multi-iron enzyme installs copper-binding oxazolone/thioamide pairs on a nontypeable Haemophilus influenzae virulence factor. *Proceedings of the National Academy of Sciences* **2024,** *121*, e2408092121.

52. Chioti, V. T.; Clark, K. A.; Ganley, J. G.; Han, E. J.; Seyedsayamdost, M. R., N-Calpha Bond Cleavage Catalyzed by a Multinuclear Iron Oxygenase from a Divergent Methanobactin-like RiPP Gene Cluster. *Journal of the American Chemical Society* **2024,** *146*, 7313-7323.

53. Kenney, G. E.; Goering, A. W.; Ross, M. O.; DeHart, C. J.; Thomas, P. M.; Hoffman, B. M.; Kelleher, N. L.; Rosenzweig, A. C., Characterization of Methanobactin from Methylosinus sp. LW4. *Journal of the American Chemical Society* **2016,** *138*, 11124-7.

54. Kim, H. J.; Graham, D. W.; DiSpirito, A. A.; Alterman, M. A.; Galeva, N.; Larive, C. K.; Asunskis, D.; Sherwood, P. M., Methanobactin, a copper-acquisition compound from methane-oxidizing bacteria. *Science* **2004,** *305*, 1612-5.

55. Behling, L. A.; Hartsel, S. C.; Lewis, D. E.; DiSpirito, A. A.; Choi, D. W.; Masterson, L. R.; Veglia, G.; Gallagher, W. H., NMR, Mass Spectrometry and Chemical Evidence Reveal a Different Chemical Structure for Methanobactin That Contains Oxazolone Rings. *Journal of the American Chemical Society* **2008,** *130*, 12604-12605.

56. Blin, K.; Shaw, S.; Augustijn, H. E.; Reitz, Z. L.; Biermann, F.; Alanjary, M.; Fetter, A.; Terlouw, B. R.; Metcalf, W. W.; Helfrich, E. J. N.; Gilles, P.; Medema, M. H.; Weber, T., antiSMASH 7.0: new and improved predictions for detection, regulation, chemical structures and visualisation. *Nucleic Acids Research* **2023,** *51*, W46-W50.

57. Terlouw, B. R.; Blin, K.; Navarro-Muñoz, J. C.; Avalon, N. E.; Chevrette, M. G.; Egbert, S.; Lee, S.; Meijer, D.; Michael; Zachary; Jeffrey; Selem-Mojica, N.; Tørring, T.; Zaroubi, L.; Alanjary, M.; Aleti, G.; Aguilar, C.; Suhad; Hannah; Luis; Barona-Gómez, F.; Bernaldo-Agüero, J.; Bielinski, V. A.; Biermann, F.; Thomas; Victor; Castelo-Branco, R.; Fernanda; Cruz-Morales, P.; Du, C.; Katherine; Gavriilidou, A.; Gayrard, D.; Gutiérrez-García, K.; Haslinger, K.; Eric; Justin; Afif; Kalkreuter, E.; Kalyvas, N.; Kyo; Kautsar, S.; Kim, W.; Aditya; Li, Y.-X.; Lin, G.-M.; Loureiro, C.; Joris; Nico; Lund, G.; Parra, J.; Philmus, B.; Pourmohsenin, B.; Lotte; Rego, A.; Devasahayam; Robinson, S.; Eve; Michelle; Darren; Kumar; Sokolova, N.; Tang, X.; Udwary, D.; Vigneshwari, A.; Vind, K.; Sophie; Waschulin, V.; Sam; Jaclyn; Thomas; Xie, H.; Yang, D.; Yu, J.; Zdouc, M.; Zhong, Z.; Collemare, J.; Roger; Weber, T.; Marnix, MIBiG 3.0: a community-driven effort to annotate experimentally validated biosynthetic gene clusters. *Nucleic Acids Research* **2023,** *51*, D603-D610.

58. Goris, J.; Konstantinidis, K. T.; Klappenbach, J. A.; Coenye, T.; Vandamme, P.; Tiedje, J. M., DNA–DNA hybridization values and their relationship to whole-genome sequence similarities. *International journal of systematic and evolutionary microbiology.* **2007,** *57*, 81-91.

59. Nishijima, M.; Takadera, T.; Imamura, N.; Kasai, H.; An, K. D.; Adachi, K.; Nagao, T.; Sano, H.; Yamasato, K., Microbulbifer variabilis sp. nov. and Microbulbifer epialgicus sp. nov., isolated from Pacific marine algae, possess a rod-coccus cell cycle in association with the growth phase. *Int J Syst Evol Microbiol* **2009,** *59*, 1696-707.

60. Ióca, L. P.; Dai, Y.; Kunakom, S.; Diaz-Espinosa, J.; Krunic, A.; Crnkovic, C. M.; Orjala, J.; Sanchez, L. M.; Ferreira, A. G.; Berlinck, R. G. S.; Eustáquio, A. S., A family of nonribosomal peptides modulate collective behavior in *Pseudovibrio* bacteria isolated from marine sponges. *Angewandte Chemie International Edition* **2021,** *60*, 15891-15898.

61. Han, S.-M.; Park, J.-S., *Ruegeria spongiae* sp. nov., isolated from *Callyspongia elongata*. *International Journal of Systematic and Evolutionary Microbiology* **2023,** *73*.
